# Bibliometric and clinical trial registry analysis of the University of Split medical research from 1997 to 2022

**DOI:** 10.3325/cmj.2025.66.153

**Published:** 2025-04

**Authors:** Ana Marušić, Igor Jerković, Petra Jelić, Dragan Ljutić

**Affiliations:** 1 University of Split School of Medicine, Split, Croatia; 2 Science Department, University Hospital of Split, Split, Croatia; 3 Rectors Office, University of Split, Split, Croatia; 4 University of Split Mediterranean Institute for Life Sciences, Split, Croatia

## Abstract

**Aim:**

To analyze the most influential publications from researchers in medicine at the University of Split in Croatia, as well as their participation in publicly registered clinical trials, to gain insight into medical research excellence in the second largest university in Croatia.

**Methods:**

For articles published in the top 5% of journals in 47 medical categories in the Web of Science Core Collection (WoSCC) from 1997, when the School of Medicine became an independent University constituent, to 2022, we analyzed the number of articles in different discipline categories, citations to these articles, and geographical and institutional collaborations. From the public ClinicalTrials.gov registry, we collected the information on the number of clinical trials at the University of Split School of Medicine and/or the University Hospital of Split.

**Results:**

Authors with a UNIST affiliation published 106 articles in the top 5% journals in 29 out of 59 WoSCC journal categories. The annual publication output significantly increased over the years. Over 80% of the articles were the result of collaboration with the global research community, involving 82 different countries. The total number of citations across these articles was 21 171, with a median of 187.4 citations per article. The first registered clinical trial in the ClinicalTrials.gov registry with a location in Split School of Medicine or the University Hospital of Split was in 1993, with a statistically significant increasing trend over the years. These trials represented 20.6% of all registered trials from Croatia.

**Conclusion:**

The University of Split has been continually increasing its impact in medical research. Bibliometric analysis should be regularly performed to follow the development of medical research at the University, identify new strategic areas for research excellence, support researchers, and attract new researchers and research teams.

Medical research plays a fundamental role in advancing medical knowledge, improving health care practices, and enhancing patient outcomes. It is essential for the discovery and refinement of novel treatments for a wide range of diseases, from rare genetic disorders to widespread chronic conditions like cancer and cardiovascular disease (1). Medical research is also the cornerstone of evidence-based medicine (EBM), which uses the best available scientific evidence to guide medical decision-making (2). Advances in clinical research have fueled the rise of personalized medicine, an approach that tailors medical treatment to individual patient characteristics, genetic makeup, and lifestyle (3). Medical research also plays a crucial role in responding to emerging public health threats, such as infectious disease outbreaks or environmental health hazards (4). It drives innovation, supports evidence-based practices, enhances patient safety, and leads to better health outcomes for individuals and populations.

Medical research is also a generator of research publications with important impact on health, and areas of medical research receiving most attention and funding are identified to reflect priorities of the scientific community and health care systems. Additionally, identifying key contributors and institutions may help facilitate collaborations and indicate potential gaps in expertise needing to be addressed at the academic level. The study of bibliometric output has emerged as an important approach to understanding the evolution, trends, and impact of scientific research, particularly in clinical sciences (5).

In Croatia, bibliometric assessment of research has been performed for different disciplines, including a 2000-2007 study focusing on public health and preventive medicine (6), a study on scientific productivity of early-career researchers from the largest Croatian medical school at the University of Zagreb in 2005 (7), as well as a 2000-2006 study of clinical and life sciences publication output from research institutions in Split (8). University of Split (UNIST), with its School of Medicine (USSM), plays a significant role in medical research and education, contributing to the advancement of medical knowledge and health care practices. The USSM was established as an independent university constituent in 1997, enabling the UNIST to develop its research capabilities in the area of medicine (9). As a key academic institution in the field of health, the USSM, with its clinical base at the University Hospital of Split, has been actively engaged in research, publishing scientific articles, and participating in clinical trials. Currently, there are 162 researchers with academic positions of assistant professor and higher at the USSM. The USSM has been involved in numerous medical research projects across different disciplines, focusing on addressing critical health challenges and contributing to evidence-based medical practices (9). On the occasion of the 50th anniversary of the UNIST, we analyzed most influential publications from UNIST researchers in medicine, as well as the participation in publicly registered clinical trials, in order to gain insight into the development of medical research in the second largest university in Croatia and to identify research priorities in medicine for the university.

## Methods

### Data sources

In this cross-sectional study, we used the InCites analytical tool (Clarivate, Graz, Austria) to retrieve articles and reviews published by UNIST researchers in the top 5% journals in medical categories of the Web of Science Core Collection (WoSCC), including the Emerging Science Citation Index (ESCI), from 1997 until 2022. 1997 was chosen because it was the time when the USSM was established as an independent university unit, and could be searched as such in the database. The inclusion criterion for being a UNIST medical researcher was to be affiliated with the USSM. Document types other than “Article” and “Review” were not included in the analysis.

The data for the analysis were retrieved based on the categories under the clinical medicine group of journals indexed in WoSCC (Clinical, Pre-Clinical & Health section). Specifically, we used Global Institutional Profiles Project Categories (https://incites.zendesk.com/hc/en-gb/articles/22586976355217-Global-Institution-Profiles-Project-Research-Areas-GIPP), and the filter “Research area,” Web of Science schema. In order to address the annual changes (mainly increase) in journals’ impact factors within decades, the selection of the top 5% papers included the consideration of annual journal impact factor position (%) with respect to the overall annual number of journals listed according to the annual impact factor within the corresponding subcategory of WoSCC medical sciences. The rank for papers was established based on the year of publication, using data sourced from the Journal Citation Reports database (Rank by Journal Impact Factor) for each subcategory, with subsequent computation of the percentage. The journals in which these papers were published have been often listed in other WoSCC categories and corresponding subcategories, but it is out of scope of present paper to discuss the journals performance in other WoSCC categories. The articles with more than 50 authors were not considered because it was difficult to evaluate reliable contribution from UNIST authors to the study.

To assess the activity of UNIST researchers related to clinical trials, we searched the largest public trials registry, ClinicalTrials.gov from the National Institutes of Health, USA, established in 1997. We used the country filter to retrieve all records of clinical trials with a site in Croatia until the end of 2023. We collected the information on the number of clinical trials where the USSM and/or the UNIST were the sponsor, collaborator, or location of a clinical trial. Time filters were not used.

### Data analysis

The results are presented as frequencies for categorical variables and as medians and interquartile ranges (IQR) for continuous variables. Time trends were assessed using Spearman’s rank correlation. Statistical analysis was performed using MedCalc version 23.1.5 (MedCalc, Ostend, Belgium).

## Results

### Bibliographical analysis

Overall, 717 articles were published in the 1st quartile of the Clinical, Pre-clinical & Health WoSCC section by authors with a USSM affiliation. Of those, 106 articles (15%) were in the top 5% journals in journal categories. Among these, the Document Type was “Article” for 87 (82%) and “Review” for 19 (18%) articles. USSM researchers were first authors on 29 (27%) articles. Because 6 journals were included in more than one category, there were 113 journal data sets for analysis of journal categories. These articles were published in journals from 29 out of 47 WoS journal categories (Table 1). The list of articles and their abstracts is available in the Supplement 1(Supplementary material 1). There were 18 WoSCC Categories without USSM articles in top journals as follows: Allergy; Emergency Medicine; Dentistry, Oral Surgery & Medicine; Dermatology; Hematology; Integrative & Complementary Medicine; Medical Ethics; Medicine, Legal; Medical Informatics; Medical Laboratory Technology; Neuroimaging; Nutrition & Dietetics; Primary Health Care; Rehabilitation; Rheumatology; Substance Abuse; Surgery; Transplantation. Research categories with more than 10 articles in top journals were Health Care Sciences & Services; Public, Environmental & Occupational Medicine; and Sport Sciences (Table 1).

**Table 1:** The number of top 5% articles published by researchers from the University of Split, and citations to these articles in the clinical medicine group of journals in Web of Science Core Collection (WoSCC)*

Subject categories	No. articles	Total No. citations	Median No. citations per article (IQR)*
1. Anesthesiology	1	23	–
2. Audiology & Speech Language Pathology	1	77	–
3. Cardiac & Cardiovascular System	6	832	145.50 (35.00-186.00)
4. Clinical Neurology	2	105	52.50
5. Critical Care Medicine	1	62	–
6. Endocrinology & Metabolism	2	2387	1193.50
7. Gastroenterology & Hepatology	4	2515	335.00 (175.50-1082.00)
8. Geriatrics & Gerontology	1	27	–
9. Health Care Sciences & Services	14	267	10.5 (4.00-24.00)
10. Infectious Diseases	5	1354	264.50 (13.00-634.00)
11. Medicine, General & Internal	7	1824	189.00 (39.25-436.00)
12. Medicine, Research & Experimental	1	897	–
13. Nursing	3	74	9.00 (2.25-51.00)
14. Obstetrics & Gynecology	2	27	13.50
15. Oncology	7	3728	343.00 (132.00-549.25)
16. Ophthalmology	1	72	–
17. Orthopedics	1	89	–
18. Othorhinolaryngology	1	77	–
19. Pathology	1	28	–
20. Pediatrics	3	2358	61.00 (22.00-1731.25)
21. Peripheral Vascular Disease	3	1683	298.00 (100.75-1087.00)
22. Pharmacology & Pharmacy	1	51	–
23. Psychiatry	4	245	66.00 (21.50-101.00)
24. Public, Environmental & Occupational Medicine	13	1074	81.00 (35.75-120.00)
25. Radiology, Nuclear Medicine & Medical Imaging	2	33	16.50
26. Respiratory System	1	62	–
27. Sport Sciences	17	693	35.00 (17.75-49.50)
28. Tropical Medicine	2	26	13.00
29. Urology & Nephrology	6	481	
Total	113	21171	76.00 (71.00-106.00)

*Six articles were included in more than one WoSCC category; the number of unique published articles was 106.

**Figure 1:**
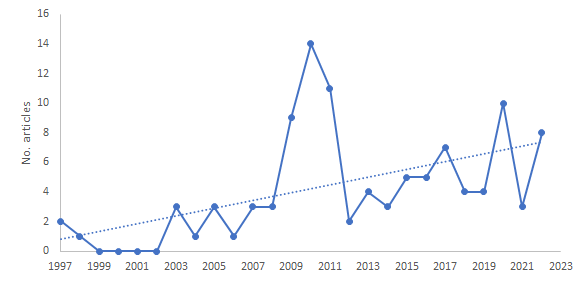
Number of articles published in top 5% journals in Web of Science Core Collection journals, 1997-2022.

The number of articles in top journals increased over time (Figure 1), with a median of 3 (IQR = 1.00-5.00) articles per year. Apart from 3 articles published in 1997-1998, the continual publication started only in 2003, when the researchers from UNIST published 3 articles (Figure 1) and continued to publish a median of 4 articles (IQR = 3.00-7.25) per year. The number of published articles had a significant increasing trend (ρ = 0.716, *P* < 0.001), with a peak in 2010 and 2011, with 14 and 11 articles published, respectively.

Most articles were published in Sport Sciences category (17 articles), followed by Health Care Sciences & Services (14 articles), and Public, Environmental & Occupational Medicine (13 articles). The total number of citations across these articles was 21 171, with a median of 10 citations per article (IQR = 59.25-1144.00). The most cited papers were in the category of Oncology (7 articles with 3728 citations), Endocrinology & Metabolism (2 articles with 2387 citations) and Pediatrics (3 articles with 2358 citations). There were five articles with more than one thousand citations: 1) a multicenter study on obesity and the risk of myocardial infarction by Yusuf et al (10) (2075 citations in WoS Core Collection and 2313 in all WoS databases); 2) a study on global burden of acute lower respiratory infections due to respiratory syncytial virus in young children by Nair et al (11) (2061 citations in WoS Core Collection and 2304 in all WoS databases); 3) an experimental study on the promotors of colitis-associated cancer by Grivennikov et al (12) (1699 citations in WoS Core Collection and 1987 in all WoS databases); 4) a review article on inflammation and colon cancer by Terzić et al (13) (1530 citations in WoS Core Collection and 1720 in all WoS databases); and 5) a multicenter study on risk factors associated with acute stroke by O’Donnell et al (14) (1256 citations in WoS Core Collection and 1370 in all WoS databases).

The first two articles from this study were published in 1997, one in *The Lancet*, describing a case of dermatomyositis due to leishmania infection (15) and the other in *Gastroenterology*, describing the expression and prognostic value of an oncogene in colorectal cancer (16). In 1998, there was another paper by Punda Polić et al in *The Lancet* (17), again a case report, on visceral leishmaniosis.

**Figure 2:**
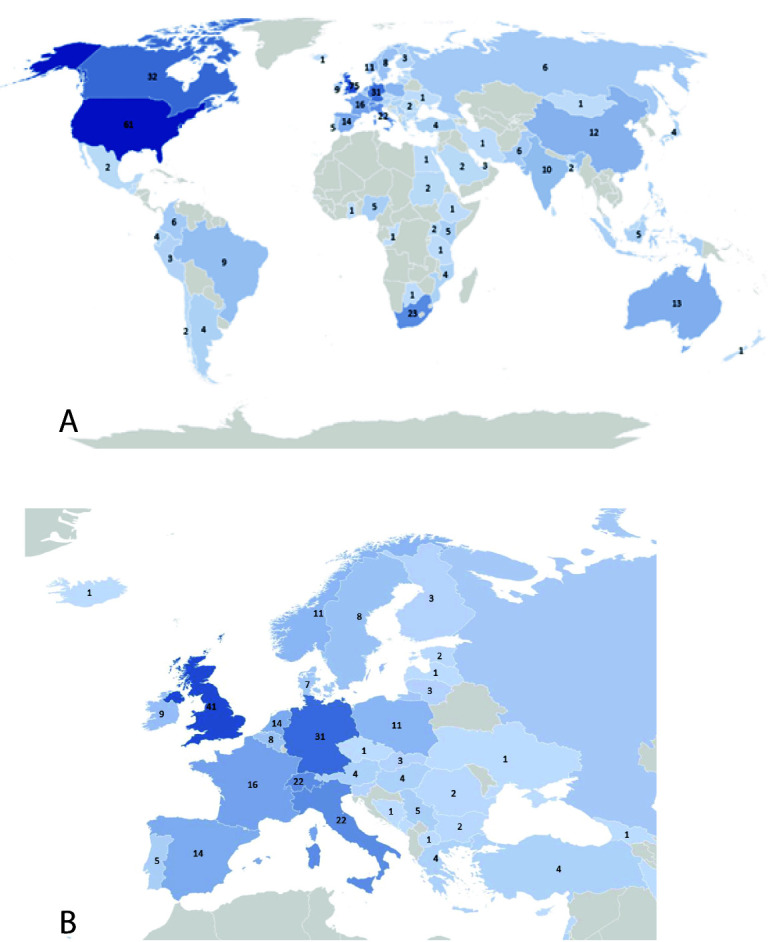
International collaboration map for the University of Split in top 5% articles in Web of Science Core Collection journals, 1980-2022. (**A**) Global collaboration. (**B**) Detailed map of European collaborations. The shading indicates the number of affiliations from different countries. For UK, the affiliations were listed as England (n = 41), Scotland (n = 33) and Wales (n = 1). Data from Incites data set includes data until 30 June 2024, updated 26 July 2024 and exported 29 August 2024. The map was created using Example: Custom Bing Map Title: International collaboration of UNIST; author: Petra Jelić; date of creation: January 7, 2024; Bing Maps Attribution: “© Australian Bureau of Statistics, Geonames, Microsoft, Navinfo, Open Places, OpenStreetMap, Overture Maps Foundation, TomTom, Zenrin.

**Figure 3:**
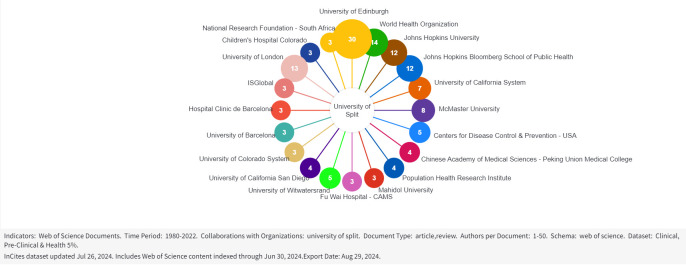
Institutions listed as collaborating affiliations for the University of Split in top 5% articles in medicine in Web of Science Core Collection journals, 1980-2022. Data from Incites data set includes data until 30 June 2024, updated 26 July 2024 and exported 29 August 2024.

Out of 106 unique articles, 7 (6.5%) had all authors affiliated with UNIST, 14 (13.0%) had co-authors from Croatia only, and 87 (80.5%) had co-authors with non-Croatian affiliations from 82 different countries (Figure 2). Most common collaborations were with institutions from USA (n = 61 affiliations), UK (n = 74), Germany (n = 35), Canada (n = 32), Italy (n = 22), Switzerland (n = 22), and South Africa (n = 19). Regarding individual institutions, researchers from the UNIST most intensely collaborated with the University of Edinburgh (n = 30 articles), followed by the John Hopkins University (n = 24), and the World Health Organization (n = 14) (Figure 3).

**Figure 4:**
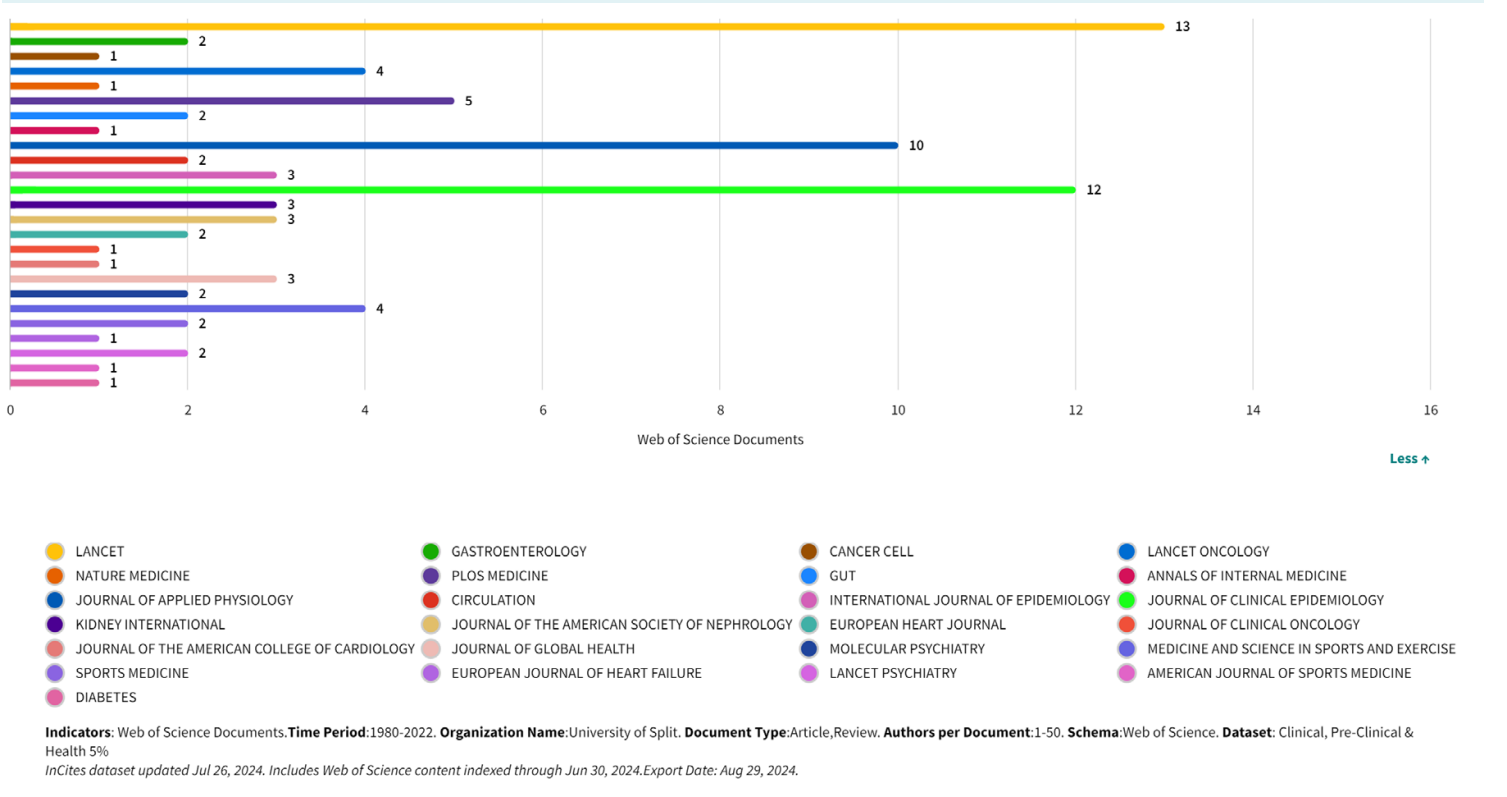
Journals in which top 5% articles in medicine with authors from the University of Split, Web of Science Core Collection journals, 1980-2022. Data from Incites data set includes data until 30 June 2024, updated 26 July 2024 and exported 29 August 2024.

**Figure 5:**
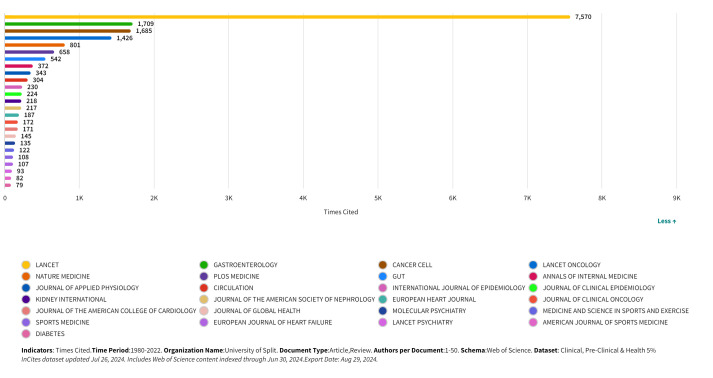
Citations to top 5% articles in medicine with authors from the University of Split in journals from the Web of Science Core Collection, 1980-2022. Data from Incites data set includes data until 30 June 2024, updated 26 July 2024 and exported 29 August 2024.

Most of the articles were published in *The Lancet* (n = 13), the *Journal of Clinical Epidemiology* (n = 12), and the *Journal of Applied Physiology* (n = 10) (Figure 4). By far the largest number of citations came from the articles published in *The Lancet*, followed by those in *Gastroenterology*, *Cancer Cell*, and *Lancet Oncology* (Figure 5).

We also analyzed InCites matching of the articles to sustainable development goals (SDGs). Almost all the articles (n = 105) addressed the SDG 3 Good Health and Well-being, 15 articles also addressed SDG5 Gender Equality, and 1 article each addressed SDG13 Climate Action and SDG14 Life Below Water.

### Analysis of trials from clinical trial registry

**Figure 6:**
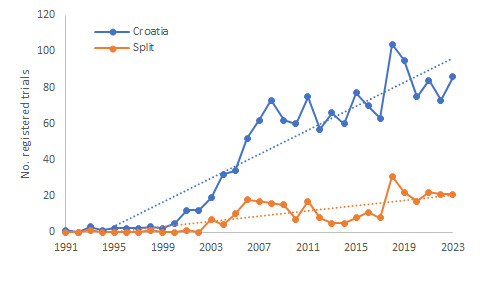
Total registered clinical trials in Croatia until 2023, and those among them with a trial location in Split.

Until the end of 2023, there were 1423 trials with Croatia mentioned in any of the registry fields in ClinicalTrials.gov (Figure 6). The first clinical trial was registered in 1991, and the number of registered trials significantly increased over time (ρ = 0.941, *P* < 0.001), particularly after 2005, when the trial registration became mandatory requirement for manuscript submission to journals. Out of all registered trials in Croatia, 293 (20.6%) involved the UNIST or USSM. In comparison, Zagreb, the capital of Croatia, with 6 academic clinical institutions had 1156 registered trials with Zagreb as a trial site, but only 79 (6.8%) as a trial sponsor or collaborator.

The first registered trial with a location in Split was in 1993 (Figure 6), related to the UNIST. The median number of trials registered annually was 6 (IQR = 1.00-13.75), with a statistically significant increasing trend over the years (ρ = 0.752, *P* < 0.001).

**Figure 7:**
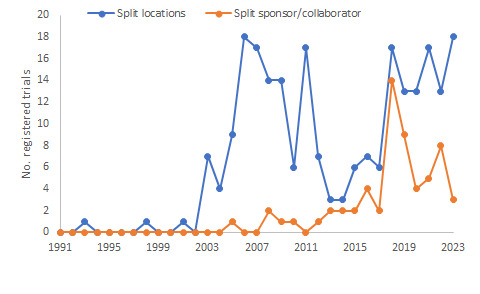
Number of clinical trials registered in *ClinicalTrials.gov* where the University of Split School of Medicine or the University Hospital of Split were primary organizers (sponsors or collaborators).

Among all trials with Split as a location, the USSM or the UNIST were either the trial sponsor for 54 trials (18.4%), a collaborator (n = 7, 2.4%), or a trial site (n = 232, 79.2%) (Figure 7). Among the trials where the USSM or the UNIST were the sponsors, most (n = 40) were interventional trials, and 14 were observational studies. Most of these trials (n = 30) were completed at the time of this study, 1 is enrolling by invitation, 1 was withdrawn, 6 were still recruiting, and 16 had an unknown status. Out of 30 completed trials, 23 had publications in journals indexed in PubMed. There were 24 journal articles, among which 1 article presented the results of 3 separate registered trials, 1 article presented the results from 2 separate registered trials, and 2 trials each had 2 separate articles published. The list of trials and their publications is presented in Supplement 2(Supplementary material 2).

The UMSS or the UNIST started to be registered as trials sponsors (organizers) or collaborators from 2005, with a significantly increasing trend in the annual number of registered trials (ρ = 0.839, *P* < 0.001), and a peak in 2018 (Figure 7). The median number of trials where Split was location or sponsor per year since 2005 was 2 (IQR = 1-4).

## Discussion

Our study demonstrated that the University of Split continues to be a center of excellence in clinical research. This is evident from the number of articles published in the top 5% most-cited journals indexed in WoS, and a steady increase in this number from 1997, the year of the establishment of the School of Medicine as the unity of the University of Split, separate from the University of Zagreb School of Medicine. The trend of increasing clinical research evidence confirms previous findings by Puljak et al (9), who reported that the annual number of publications from clinical and life-science researchers from all institutions in Split tripled in the 2000-2006 period. The USSM and the UNIST, as two major clinical research institutions in Split, have demonstrated their excellence in terms of overall publications as well as publications in journals with the highest impact in the global research community.

Excellence in published clinical research from the Split University was most prominent in the journals from three WoS categories. A high number of articles in top journals from WoS categories Health Care Sciences & Services; Public, Environmental & Occupational Medicine can be related to the establishment of the Croatian Centre for Global Health (18) and the Cochrane Croatia (19) as research units of the USSM in 2008. These two centers brought together researchers from different disciplines and their joint research efforts, including the creation of the “10001 Dalmatians” biobank for genomic and metabolomic studies (20), resulted in a sharp increase in high-quality articles in 2009, 2010, and 2011. The high number of articles in the journal Web Sport Sciences category are the results of the oldest research group at USSM, focused on the physiology of diving and integrative physiology (21). Efforts from all research groups at the USSM and the University Hospital of Split also resulted in strong international research collaborations, with collaborators from 82 countries, clearly demonstrating that Split University is a part of the global research community. Global relevance of medical research at the UNIST is also visible in its research efforts contributing to several UN sustainable development goals (SDG): SDG3 – Ensure healthy lives and promote well-being for all at all ages; SDG9 – Build resilient infrastructure, promote inclusive and sustainable industrialization and foster innovation; and SDG16 – Promote peaceful and inclusive societies for sustainable development, provide access to justice for all, and build effective, accountable, and inclusive institutions at all levels (22). Our study of the publication output demonstrated that medical research predominantly contributed to SDG3, but also opened the possibility to expand its contributions to other SDGs, such as SDG5 Gender Equality, SDG13 Climate Action, and SDG14 Life Below Water. The three latter SDG have the potential to be further developed at the USSM.

It is difficult to make comparisons of citations to papers from UNIST with that of other medical schools in Croatia, as there are no comparative studies for other universities or medical schools. We only found the 2012 study on the publication output of the Department of Internal Medicine, University of Zagreb School of Medicine and the University Hospital Center Zagreb, which observed that clinical practice guidelines and randomized clinical trials received most citations (an average of 157 and 76 citations, respectively) (23). In our sample of publications, we had much higher citation rates, primarily from the categories of Oncology (7 articles with 3728 citations), Endocrinology & Metabolism (2 articles with 2387 citations) and Pediatrics (3 articles with 2358 citations). The differences in citation numbers partly reflect the time difference between the two studies, which necessarily resulted in the higher citation numbers in our study, as well as the increased number of indexed journals over time. Further research is needed to dissect in detail the publication output in medicine from different universities in Croatia.

Our study is the first to describe in more detail research activities related to clinical trials in Croatia. In our previous study from 2017, we showed that, despite the strong legal provisions for clinical trial regulation, the information about clinical trials is not very transparent and visible to the public (24). Our analysis in this study shows that, according to the largest trial registry, ClinicalTrial.gov, the number of registered clinical trials increased since 2000, with a steady increase since 2004, reaching 86 registered trials in 2023. These numbers do not represent the total number of clinical trials in Croatia, because there was no legal mandate for trial registration before 2005 (24). This mandate was brought about by an international movement led by journal editors of the International Committee of Medical Journal Editors (ICMJE), the most influential group in medical publishing, to increase the transparency and trustworthiness of clinical trials (25). *Croatian Medical Journal*, the official journal of the USSM was a member of the ICMJE and actively contributed to the development of the trial registration mandate and legislation (26). Most of the clinical trials in Croatia are performed in Zagreb, which has two university hospital centers and two university hospitals (27). Split has a single university hospital center (UNIST) and, therefore, has lower numbers of trials and slower increase in their number over the years. However, Split performed better in the number of registered clinical trials in which either USSM or the UNIST were sponsors or collaborators (20.9% of all registered trials, compared with 6.8% of all trials in Zagreb). This demonstrates the active approach and research initiative of clinical researchers in Split, who performed investigator-driven trials.

The limitations of our study are related to the inherent biases related to the information sources used in data collection. WoS represents only a small, albeit most influential, fraction of journals (28). However, the aim of this study was to explore medical research from the UNIST that goes beyond the state of the art, to showcase research excellence and provide evidence for future research policies and plans for the university. Our study is also restricted to a single university. It would be interesting to compare the publication output with other universities in Croatia, but a study of high-impact articles may be biased because the USSM is a relatively new institution, established in 1997, while other research-intensive medical schools have much longer research tradition: the University of Zagreb School of Medicine was established in 1917 and the University of Rijeka School of Medicine in 1955. The limitation related to the registry analysis is that the ClinicalTrials.gov is not the only trial registry and is included in the World Health Organization (WHO) International Clinical Trials Registry Platform (ICTRP), which also includes the EU Clinical Trial Register and a number of national registries (29). We chose ClinicalTrials.gov for our study as it is the largest global registry, which has high-quality data and includes many of the trials from other registries (30). Also, a reliable analysis of clinical trials in Croatia was possible only after 2005, when the trial registration became a mandatory requirement for journal manuscript submission. We included all registered trials with the Split location in this analysis, but we plan a more extensive analysis of clinical trials in Croatia in different registries.

Instead of a conclusion, we can offer ideas about the future prospects for medical research at the UNIST and its affiliated hospital. The UNIST should continue and increase support for medical research as the best showcase for research excellence at the University. Research efforts of already outstanding research groups and topics should be continued, particularly in relation to the support to acquire international funding. Research areas that have a great potential for excellent research but have not yet been published in top journals, should be supported in their efforts. Finally, analyses such as in this study should be regularly performed to follow the development of medical research at the University, identify new areas for excellence, support researchers, and attract both early-career and experienced researchers from all over the world to Split. Only by promoting and sustaining research excellence, can the UNIST take the position in the global research community that it deserves.
