# Supplementary material for: Bibliometric and clinical trial registry analysis of the University of Split medical research from 1997 to 2022
Source: Croat Med J. 2025 Apr;66(2):153–63. doi: 10.3325/cmj.2025.66.153 (PMC12093127; doi:10.3325/cmj.2025.66.153)
Supplement: Supplementary Material 1 [file CroatMedJ_66_s014.pdf]

Supplementary table 1. Journal articles published in 5% top journals indexed in the Web of Science Core collection, 1980-2022, according to the Clinical Medicine categories

	Article title	Abstract
	**Anesthesiology**	
1.	Sapunar D, Vukojević K, Kostić S, Puljak L. Attenuation of pain-related behavior evoked by injury through blockade of neuropeptide Y Y2 receptor. Pain. 2011 May;152(5):1173-1181. doi: 10.1016/j.pain.2011.01.045.	Neuropeptide Y (NPY) has an important but still insufficiently defined role in pain modulation. We therefore examined the ability of NPY to modulate experimentally induced neuropathic pain by injecting it directly into dorsal root ganglion (DRG) immediately following spinal nerve ligation (SNL) injury. We have found that this application exacerbates pain-related behavior induced by SNL in a modality-specific fashion. When saline was injected after SNL, the expected increase in hyperalgesia responses to needle stimulation was present on the 8th postoperative day. When we injected NPY, hyperalgesic responses were increased in a manner similar to the SNL/saline group. To characterize NPY action, specific Y1 and Y2 antagonists were also delivered directly to DRG, which revealed that behavioral actions of NPY were abolished by Y2 receptor antagonist. We tested whether NPY effects were the result of its role in immunity by immunohistochemical staining for glial fibrillary acidic protein, in order to identify activation of DRG satellite cells and dorsal horn astrocytes. Exacerbation of pain-related behavior following NPY injection was accompanied by astrocyte activation in ipsilateral dorsal horn and with satellite cells activation in the DRG proximal to injury. This activation was reduced following Y2 receptor antagonist application. These findings indicate an important link between pain-related behavior and neuroimmune activation by NPY through its Y2 receptor. (C) 2011 International Association for the Study of Pain. Published by Elsevier B.V. All rights reserved.
	**Audiology & Speech Language Pat**	
2.	Rudic M, Keogh I, Wagner R, Wilkinson E, Kiros N, Ferrary E, Sterkers O, Bozorg Grayeli A, Zarkovic K, Zarkovic N. The pathophysiology of otosclerosis: Review of current research. Hear Res. 2015 Dec;330(Pt A):51-6. doi: 10.1016/j.heares.2015.07.014.	Otosclerosis is a complex disease of the human otic capsule with highest incidence in adult Caucasians. So far, many possible etiological factors like genetics, HLA, autoimmunity, viruses, inflammation, and hormones have been investigated but still the development of the disease remains unclear. Currently, the surgical replacement of stapes (stapedotomy) remains the best possible treatment option. In this review, we analyze different etiological factors studied so far in otosclerosis pathophysiology and discuss most recent findings and possible new research pathways. This article is part of a Special Issue entitled . © 2015 Elsevier B.V. All rights reserved.

	Cardiac & Cardiovascular System	
3.	McGorrian C, Yusuf S, Islam S, Jung H, Rangarajan S, Avezum A, Prabhakaran D, Almahmeed W, Rumboldt Z, Budaj A, Dans AL, Gerstein HC, Teo K, Anand SS; INTERHEART Investigators. Estimating modifiable coronary heart disease risk in multiple regions of the world: the INTERHEART Modifiable Risk Score. Eur Heart J. 2011 Mar;32(5):581-9. doi: 10.1093/eurheartj/ehq448.	Aims Summating risk factor burden is a useful approach in the assessment of cardiovascular risk among apparently healthy individuals. We aimed to derive and validate a new score for myocardial infarction (MI) risk using modifiable risk factors, derived from the INTERHEART case-control study (n = 19 470).<br>Methods and results Multiple logistic regression was used to create the INTERHEART Modifiable Risk Score (IHMRS). Internal validation was performed using split-sample methods. External validation was performed in an international prospective cohort study. A risk model including apolipoproteins, smoking, second-hand smoke exposure, hypertension, and diabetes was developed. Addition of further modifiable risk factors did not improve score discrimination in an external cohort. Split-sample validation studies 2 san2er2 area under the receiver-operating characteristic (ROC) curve c-statistic of 0.71 [95% confidence interval (CI): 0.70, 0.72]. The IHMRS was positively associated with incident MI in a large cohort of people at low risk for cardiovascular disease [12% increase in MI risk (95% CI: 8, 16%) with a 1-point increase in score] and showed appropriate discrimination in this cohort (ROC c-statistic 0.69, 95% CI: 0.64, 0.74). Results were consistent across ethnic groups and geographic regions. A non-laboratory-based score is also supplied.<br>Conclusions Using multiple modifiable risk factors from the INTERHEART case-control study, we have developed and validated a simple score for MI risk which is applicable to an international population.
4.	Doehner W, Ural D, Haeusler KG, Čelutkienė J, Bestetti R, Cavusoglu Y, Peña-Duque MA, Glavas D, Iacoviello M, Laufs U, Alvear RM, Mbakwem A, Piepoli MF, Rosen SD, Tsivgoulis G, Vitale C, Yilmaz MB, Anker SD, Filippatos G, Seferovic P, Coats AJS, Ruschitzka F. Heart and brain interaction in patients with heart failure: overview and proposal for a taxonomy. A position paper from the Study Group on Heart and Brain Interaction of the Heart Failure Association. Eur J Heart Fail. 2018 Feb;20(2):199-215. doi: 10.1002/ejhf.1100.	Heart failure (HF) is a complex clinical syndrome with multiple interactions between the failing myocardium and cerebral (dys-)functions. Bi-directional feedback interactions between the heart and the brain are inherent in the pathophysiology of HF: (i) the impaired cardiac function affects cerebral structure and functional capacity, and (ii) neuronal signals impact on the cardiovascular continuum. These interactions contribute to the symptomatic presentation of HF patients and affect many co-morbidities of HF. Moreover, neuro-cardiac feedback signals significantly promote aggravation and further progression of HF and are causal in the poor prognosis of HE The diversity and complexity of heart and brain interactions make it difficult to develop a comprehensive overview. In this paper a systematic approach is proposed to develop a comprehensive atlas of related conditions, signals and disease mechanisms of the interactions between the heart and the brain in HF. The proposed taxonomy is based on pathophysiological principles. Impaired perfusion of the brain may represent one major category, with acute (cardio-embolic) or chronic (haemodynamic failure) low perfusion being sub-categories with mostly different

		consequences (i.e. ischaemic stroke or cognitive impairment, respectively). Further categories include impairment of higher cortical function (mood, cognition), of brain stem function (sympathetic over-activation, neuro-cardiac reflexes). Treatment-related interactions could be categorized as medical, interventional and device-related interactions. Also interactions due to specific diseases are categorized. A methodical approach to categorize the interdependency of heart and brain may help to integrate individual research areas into an overall picture.
5.	Smith NL, Chen MH, Dehghan A, Strachan DP, Basu S, Soranzo N, Hayward C, Rudan I, Sabater-Lleal M, Bis JC, de Maat MP, Rumley A, Kong X, Yang Q, Williams FM, Vitart V, Campbell H, Mälarstig A, Wiggins KL, Van Duijn CM, McArdle WL, Pankow JS, Johnson AD, Silveira A, McKnight B, Uitterlinden AG; Wellcome Trust Case Control Consortium;; Aleksic N, Meigs JB, Peters A, Koenig W, Cushman M, Kathiresan S, Rotter JI, Bovill EG, Hofman A, Boerwinkle E, Tofler GH, Peden JF, Psaty BM, Leebeek F, Folsom AR, Larson MG, Spector TD, Wright AF, Wilson JF, Hamsten A, Lumley T, Witteman JC, Tang W, O'Donnell CJ. Novel associations of multiple genetic loci with plasma levels of factor VII, factor VIII, and von Willebrand factor: The CHARGE (Cohorts for Heart and Aging Research in Genome Epidemiology) Consortium. Circulation. 2010 Mar 30;121(12):1382-92. doi: 10.1161/CIRCULATIONAHA.109.869156. Epub 2010 Mar 15. Erratum in: Circulation.2010 Jul 20;122(3):e399.	Background-Plasma levels of coagulation factors VII (FVII), VIII (FVIII), and von Willebrand factor (vWF) influence risk of hemorrhage and thrombosis. We conducted genome-wide association studies to identify new loci associated with plasma levels.<br>Methods and Results-The setting of the study included 5 community-based studies for discovery comprising 23 608 European-ancestry participants: Atherosclerosis Risk In Communities Study, Cardiovascular Health Study, British 1958 Birth Cohort, Framingham Heart Study, and Rotterdam Study. All subjects had genome-wide single-nucleotide polymorphism (SNP) scans and at least 1 phenotype measured: FVII activity/antigen, FVIII activity, and vWF antigen. Each study used its genotype data to impute to HapMap SNPs and independently conducted association analyses of hemostasis measures using an additive genetic model. Study findings were combined by meta-analysis. Replication was conducted in 7604 participants not in the discovery cohort. For FVII, 305 SNPs exceeded the genome-wide significance threshold of 5.0X10(-8) and comprised 5 loci on 5 chromosomes: 2p23 (smallest P value 6.2X10(-24)), 4q25 (3.6X10(-12)), 11q12 (2.0X10(-10)), 13q34 (9.0X10(-259)), and 20q11.2 (5.7X10(-37)). Loci were within or near genes, including 4 new candidate genes and F7 (13q34). For vWF, 400 SNPs exceeded the threshold and marked 8 loci on 6 chromosomes: 6q24 (1.2X10(-22)), 8p21 (1.3X10(-16)), 9q34 (<5.0X10(-324)), 12p13 (1.7X10(-32)), 12q23 (7.3X10(-10)), 12q24.3 (3.8X10(-11)), 14q32 (2.3X10(-10)), and 19p13.2 (1.3X10(-9)). All loci were within genes, including 6 new candidate genes, as well as ABO (9q34) and VWF (12p13). For FVIII, 5 loci were identified and overlapped vWF findings. Nine of the 10 new findings were replicated.<br>Conclusions-New genetic associations were discovered outside previously known biological pathways and may point to novel prevention and treatment targets of hemostasis disorders. (Circulation. 2010; 121: 1382-1392.)
6.	Giblett JP, Matetic A, Jenkins D, Ng CY, Venuraju S, MacCarthy T, Vibhishanan J, O'Neill JP, Kirmani BH, Pullan DM, Stables RH, Andrews J, Buttinger N, Kim WC, Kanyal R, Butler MA, Butler R, George S,	Aims Post-infarction ventricular septal defect (PIVSD) is a mechanical complication of acute myocardial infarction (AMI) with a poor prognosis. Surgical repair is the mainstay of treatment, although percutaneous closure is increasingly undertaken. Methods and resuts Patients treated with surgical or percutaneous repair of PIVSD (2010-2021) were identified

	Khurana A, Crossland DS, Marczak J, Smith WHT, Thomson JDR, Bentham JR, Clapp BR, Buch M, Hayes N, Byrne J, MacCarthy P, Aggarwal SK, Shapiro LM, Turner MS, de Giovanni J, Northridge DB, Hildick-Smith D, Mamas MA, Calvert PA. Post-infarction ventricular septal defect: percutaneous or surgical management in the UK national registry. Eur Heart J. 2022 Dec 21;43(48):5020-5032. doi: 10.1093/eurheartj/ehac511.	at 16 UK centres. Case note review was undertaken. The primary outcome was long-term mortality. Patient groups were allocated based upon initial management (percutaneous or surgical). Three-hundred sixty-two patients received 416 procedures (131 percutaneous, 231 surgery). 16.1% of percutaneous patients subsequently had surgery. 7.8% of surgical patients subsequently had percutaneous treatment. Times from AMI to treatment were similar [percutaneous 9 (6-14) vs. surgical 9 (4-22) days, P = 0.18]. Surgical patients were more likely to have cardiogenic shock (62.8% vs. 51.9%, P = 0.044). Percutaneous patients were substantially older [72 (64-77) vs. 67 (61-73) years, P < 0.001] and more likely to be discussed in a heart team setting. There was no difference in long-term mortality between patients (61.1% vs. 53.7%, P = 0.17). In-hospital mortality was lower in the surgical group (55.0% vs. 44.2%, P = 0.048) with no difference in mortality after hospital discharge (P = 0.65). Cardiogenic shock [adjusted hazard ratio (aHR) 1.97 (95% confidence interval 1.37-2.84), P < 0.001), percutaneous approach [aHR 1.44 (1.01-2.05), P = 0.042], and number of vessels with coronary artery disease [aHR 1.22 (1.01-1.47), P = 0.043] were independently associated with long-term mortality. Conclusion Surgical and percutaneous repair are viable options for management of PIVSD. There was no difference in post-discharge long-term mortality between patients, although in-hospital mortality was lower for surgery.
7.	Reddy VY, Anic A, Koruth J, Petru J, Funasako M, Minami K, Breskovic T, Sikiric I, Dukkipati SR, Kawamura I, Neuzil P. Pulsed Field Ablation in Patients With Persistent Atrial Fibrillation. J Am Coll Cardiol. 2020 Sep 1;76(9):1068-1080. doi: 10.1016/j.jacc.2020.07.007.	BACKGROUND Unlike for paroxysmal atrial fibrillation (AF), pulmonary vein isolation (PVI) alone is considered insufficient for many patients with persistent AF. Adjunctive ablation of the left atrial posterior wall (LAPW) may improve outcomes, but is limited by both the difficulty of achieving lesion durability and concerns of damage to the esophagus-situated behind the LAPW.<br>OBJECTIVES This study sought to assess the safety and lesion durability of pulsed field ablation (PFA) for both PVI and LAPW ablation in persistent AF.<br>METHODS PersAFOne is a single-arm study evaluating biphasic, bipolar PFA using a multispline catheter for PVI and LAPW ablation under intracardiac echocardiographic guidance. A focal PFA catheter was used for cavotricuspid isthmus ablation. No esophageal protection strategy was used. Invasive remapping was mandated at 2 to 3 months to assess lesion durability.<br>RESULTS In 25 patients, acute PVI (96 of 96 pulmonary veins [PVs]; mean ablation time: 22 min; interquartile range [IQR]: 15 to 29 min) and LAPW ablation (24 of 24 patients; median ablation time: 10 min; IQR: 6 to 13 min) were 100% acutely successful with the multispline PFA catheter alone. Using the focal PFA catheter, acute cavotricuspid isthmus block was achieved in 13 of 13 patients (median: 9 min; IQR: 6 to 12 min). The median total procedure time was 125 min (IQR: 108 to 166 min) (including a median of 28 min [IQR: 25

		to 33 min] for voltage mapping), with a median of 16 min (IQR: 12 to 23 min) fluoroscopy. Post-procedure esophagogastroduodenoscopy and repeat cardiac computed tomography revealed no mucosal lesions or PV narrowing, respectively. Invasive remapping demonstrated durable isolation (defined by entrance block) in 82 of 85 PVs (96%) and 21 of 21 LAPWs (100%) treated with the pentaspline catheter. In 3 patients, there was localized scar regression of the LAPW ablation, albeit without conduction breakthrough. CONCLUSIONS The unique safety profile of PFA potentiated efficient, safe, and durable PVI and LAPW ablation. This extends the potential role of PFA beyond paroxysmal to persistent forms of AF. © 2020 The Authors. Published by Elsevier on behalf of the American College of Cardiology Foundation. This 5 san open access article under the CC BY-NC ND license (http://creativecommons.org/licenses/by-nc-nd/4.0/).
8.	Peng J, Vongpatanasin W, Sacharidou A, Kifer D, Yuhanna IS, Banerjee S, Tanigaki K, Polasek O, Chu H, Sundgren NC, Rohatgi A, Chambliss KL, Lauc G, Mineo C, Shaul PW. Supplementation With the Sialic Acid Precursor N-Acetyl-D-Mannosamine Breaks the Link Between Obesity and Hypertension. Circulation. 2019 Dec 10;140(24):2005-2018. doi: 10.1161/CIRCULATIONAHA.119.043490.	BACKGROUND: Obesity-related hypertension is a common disorder, and attempts to combat the underlying obesity are often unsuccessful. We previously revealed that mice globally deficient in the inhibitory immunoglobulin G (IgG) receptor Fc gamma RIIB are protected from obesity-induced hypertension. However, how Fc gamma RIIB participates is unknown. Studies were designed to determine if alterations in IgG contribute to the pathogenesis of obesity-induced hypertension.<br>METHODS: Involvement of IgG was studied using IgG mu heavy chain-null mice deficient in mature B cells and by IgG transfer. Participation of Fc gamma RIIB was interrogated in mice with global or endothelial cell-specific deletion of the receptor. Obesity was induced by high-fat diet (HFD), and blood pressure (BP) was measured by radiotelemetry or tail cuff. The relative sialylation of the Fc glycan on mouse IgG, which influences IgG activation of Fc receptors, was evaluated by Sambucus nigra lectin blotting. Effects of IgG on endothelial NO synthase were assessed in human aortic endothelial cells. IgG Fc glycan sialylation was interrogated in 3442 human participants by mass spectrometry, and the relationship between sialylation and BP was evaluated. Effects of normalizing IgG sialylation were determined in HFD-fed mice administered the sialic acid precursor N-acetyl-D-mannosamine (ManNAc).<br>RESULTS: Mice deficient in B cells were protected from obesity-induced hypertension. Compared with IgG from control chow-fed mice, IgG from HFD-fed mice was hyposialylated, and it raised BP when transferred to recipients lacking IgG; the hypertensive response was absent if recipients were Fc gamma RIIB-deficient. Neuraminidase-treated IgG lacking the Fc glycan terminal sialic acid also raised BP. In cultured endothelial cells, via Fc gamma RIIB, IgG from HFD-fed mice and neuraminidase-treated IgG inhibited vascular endothelial growth factor activation of endothelial NO

		synthase by altering endothelial NO synthase phosphorylation. In humans, obesity was associated with lower IgG sialylation, and systolic BP was inversely related to IgG sialylation. Mice deficient in Fc gamma RIIB in endothelium were protected from obesity-induced hypertension. Furthermore, in HFD-fed mice, ManNAc normalized IgG sialylation and prevented obesity-induced hypertension.<br>CONCLUSIONS: Hyposialylated IgG and Fc gamma RIIB in endothelium are critically involved in obesity-induced hypertension in mice, and supportive evidence was obtained in humans. Interventions targeting these mechanisms, such as ManNAc supplementation, may provide novel means to break the link between obesity and hypertension.
	**Clinical Neurology**	
9.	Langhorne P, O'Donnell MJ, Chin SL, Zhang H, Xavier D, Avezum A, Mathur N, Turner M, MacLeod MJ, Lopez-Jaramillo P, Damasceno A, Hankey GJ, Dans AL, Elsayed A, Mondo C, Wasay M, Czlonkowska A, Weimar C, Yusufali AH, Hussain FA, Lisheng L, Diener HC, Ryglewicz D, Pogosova N, Iqbal R, Diaz R, Yusoff K, Oguz A, Wang X, Penaherrera E, Lanas F, Ogah OS, Ogunniyi A, Iversen HK, Malaga G, Rumboldt Z, Magazi D, Nilanont Y, Rosengren A, Oveisgharan S, Yusuf S; INTERSTROKE collaborators. Practice patterns and outcomes after stroke across countries at different economic levels (INTERSTROKE): an international observational study. Lancet. 2018 May 19;391(10134):2019-2027. doi: 10.1016/S0140-6736(18)30802-X.	Background Stroke disproportionately affects people in low-income and middle-income countries. Although improvements in stroke care and outcomes have been reported in high-income countries, little is known about practice and outcomes in low and middle-income countries. We aimed to compare patterns of care available and their association with patient outcomes across countries at different economic levels.<br>Methods We studied the patterns and effect of practice variations (ie, treatments used and access to services) among participants in the INTERSTROKE study, an international observational study that enrolled 13447 stroke patients from 142 clinical sites in 32 countries between Jan 11, 2007, and Aug 8, 2015. We supplemented patient data with a questionnaire about health-care and stroke service facilities at all participating hospitals. Using univariate and multivariate regression analyses to account for patient casemix and service clustering, we estimated the association between services available, treatments given, and patient outcomes (death or dependency) at 1 month.<br>Findings We obtained full information for 12342 (92%) of 13447 INTERSTROKE patients, from 108 hospitals in 28 countries; 2576 from 38 hospitals in ten high-income countries and 9766 from 70 hospitals in 18 low and middle-income countries. Patients in low-income and middle-income countries more often had severe strokes, intracerebral haemorrhage, poorer access to services, and used fewer investigations and treatments ($p<0.0001$) than those in high-income countries, although only differences in patient characteristics explained the poorer clinical outcomes in low and middle-income countries. However across all countries, irrespective of economic level, access to a stroke unit was associated with improved use of investigations and treatments, access to other rehabilitation services, and improved survival without severe dependency (odds ratio [OR] 1.29; 95% CI 1.14-1.44; all $p<0.0001$), which was independent of patient casemix characteristics and other measures of care. Use of acute antiplatelet treatment was associated with improved

		survival (1.39; 1.12-1.72) irrespective of other patient and service characteristics. Interpretation Evidence-based treatments, diagnostics, and stroke units were less commonly available or used in low and middle-income countries. Access to stroke units and appropriate use of antiplatelet treatment were associated with improved recovery. Improved care and facilities in low-income and middle-income countries are essential to improve outcomes.
10.	Squair JW, Lee AHX, Sarafis ZK, Coombs G, Barak O, Cragg JJ, Mijacika T, Pecotic R, Krassioukov AV, Dogas Z, Dujic Z, Phillips AA. Sleep-disordered breathing is associated with brain vascular reactivity in spinal cord injury. Neurology. 2019 Dec 10;93(24):e2181-e2191. doi: 10.1212/WNL.0000000000008619.	To determine the population-level odds of individuals with spinal cord injury (SCI) experiencing fatigue and sleep apnea, to elucidate relationships with level and severity of injury, and to examine associations with abnormal cerebrovascular responsiveness.<br>Methods<br>We used population-level data, meta-analyses, and primary physiologic assessments to provide a large-scale integrated assessment of sleep-related complications after SCI. Population-level and meta-analyses included more than 60,000 able-bodied individuals and more than 1,800 individuals with SCI. Physiologic assessments were completed on a homogenous sample of individuals with cervical SCI and matched controls. We examined the prevalence of (1) self-reported chronic fatigue, (2) clinically identified sleep apnea, and 3) cerebrovascular responsiveness to changing CO2.<br>Results<br>Logistic regression revealed a 7-fold elevated odds of chronic fatigue after SCI (odds ratio [OR] 7.9, 95% confidence interval [CI] 3.5-16.2), and that fatigue and trouble sleeping are correlated with the level and severity of injury. We further show that those with SCI experience elevated risk of clinically defined sleep-disordered breathing in more than 600 individuals with SCI (pooled OR 3.1, 95% CI 1.3-7.5). We confirmed that individuals with SCI experience a high rate of clinically defined sleep apnea using primary polysomnography assessments. We then provide evidence using syndromic analysis that sleep-disordered breathing is a factor strongly associated with impaired cerebrovascular responsiveness to CO2 in patients with SCI.<br>Conclusions<br>Individuals with SCI have an increased prevalence of sleep-disordered breathing, which may partially underpin their increased risk of stroke. There is thus a need to integrate sleep-related breathing examinations into routine care for individuals with SCI.
	**Critical Care Medicine**	
11.	Obeidat M, Hao K, Bossé Y, Nickle DC, Nie Y, Postma DS, Laviolette M, Sandford AJ, Daley DD, Hogg JC, Elliott WM, Fishbane N, Timens W, Hysi PG, Kaprio J,	Background Lung function measures reflect the physiological state of the lung, and are essential to the diagnosis of chronic obstructive pulmonary disease (COPD). The SpiroMeta-CHARGE consortium undertook the largest genome-wide association study

	Wilson JF, Hui J, Rawal R, Schulz H, Stubbe B, Hayward C, Polasek O, Järvelin MR, Zhao JH, Jarvis D, Kähönen M, Franceschini N, North KE, Loth DW, Brusselle GG, Smith AV, Gudnason V, Bartz TM, Wilk JB, O'Connor GT, Cassano PA, Tang W, Wain LV, Soler Artigas M, Gharib SA, Strachan DP, Sin DD, Tobin MD, London SJ, Hall IP, Paré PD. Molecular mechanisms underlying variations in lung function: a systems genetics analysis. Lancet Respir Med. 2015 Oct;3(10):782-95. doi: 10.1016/S2213-2600(15)00380-X. Epub 2015 Sep 21. Erratum in: Lancet Respir Med. 2015 Dec;3(12):e44. doi: 10.1016/S2213-2600(15)00478-6.	(GWAS) so far (n=48 201) for forced expiratory volume in 1 s (FEV1) and the ratio of FEV1 to forced vital capacity (FEV1/FVC) in the general population. The lung expression quantitative trait loci (eQTLs) study mapped the genetic architecture of gene expression in lung tissue from 1111 individuals. We used a systems genetics approach to identify single nucleotide polymorphisms (SNPs) associated with lung function that act as eQTLs and change the level of expression of their target genes in lung tissue; termed eSNPs.<br>Methods The SpiroMeta-CHARGE GWAS results were integrated with lung eQTLs to map eSNPs and the genes and pathways underlying the associations in lung tissue. For comparison, a similar analysis was done in peripheral blood. The lung mRNA expression levels of the eSNP-regulated genes were tested for associations with lung function measures in 727 individuals. Additional analyses identified the pleiotropic effects of eSNPs from the published GWAS catalogue, and mapped enrichment in regulatory regions from the ENCODE project. Finally, the Connectivity Map database was used to identify potential therapeutics in silico that could reverse the COPD lung tissue gene signature.<br>Findings SNPs associated with lung function measures were more likely to be eQTLs and vice versa. The integration mapped the specific genes underlying the GWAS signals in lung tissue. The eSNP-regulated genes were enriched for developmental and inflammatory pathways; by comparison, SNPs associated with lung function that were eQTLs in blood, but not in lung, were only involved in inflammatory pathways. Lung function eSNPs were enriched for regulatory elements and were over-represented among genes showing differential expression during fetal lung development. An mRNA gene expression signature for COPD was identified in lung tissue and compared with the Connectivity Map. This in-silico drug repurposing approach suggested several compounds that reverse the COPD gene expression signature, including a nicotine receptor antagonist. These findings represent novel therapeutic pathways for COPD.<br>Interpretation The system genetics approach identified lung tissue genes driving the variation in lung function and susceptibility to COPD. The identification of these genes and the pathways in which they are enriched is essential to understand the pathophysiology of airway obstruction and to identify novel therapeutic targets and biomarkers for COPD, including drugs that reverse the COPD gene signature in silico.
	**Endocrinology & Metabolism**	
12.	Thanabalasingham G, Huffman JE, Kattla JJ, Novokmet M, Rudan I, Gloyn AL, Hayward C, Adamczyk B, Reynolds RM, Muzinic A, Hassanali N, Pucic M, Bennett AJ, Essafi A, Polasek O, Mughal SA,	A recent genome-wide association study identified hepatocyte nuclear factor 1-alpha (HNF1A) as a key regulator of fucosylation. We hypothesized that loss-of-function HNF1A mutations causal for maturity-onset diabetes of the young (MOD?) would display altered fucosylation of N-linked glycans on plasma proteins and that glycan biomarkers could

	Redzic I, Primorac D, Zgaga L, Kolcic I, Hansen T, Gasperikova D, Tjora E, Strachan MW, Nielsen T, Stanik J, Klimes I, Pedersen OB, Njølstad PR, Wild SH, Gyllensten U, Gornik O, Wilson JF, Hastie ND, Campbell H, McCarthy MI, Rudd PM, Owen KR, Lauc G, Wright AF. Mutations in HNF1A result in marked alterations of plasma glycan profile. Diabetes. 2013 Apr;62(4):1329-37. doi: 10.2337/db12-0880.	improve the efficiency of a diagnosis of HNF1A-MODY. In a pilot comparison of 33 subjects with HNF1A-MODY and 41 subjects with type 2 diabetes, 15 of 29 glycan measurements differed between the two groups. The DG9-glycan ©, which is the ratio of fucosylated to nonfucosylated triantennary glycans, provided optimum discrimination in the pilot study and was examined further among additional subjects with HNF1A-MODY (n = 188), glucokinase (GCE)-MODY (n = 118), hepatocyte nuclear factor 4-alpha (HNF4A)-MODY (n = 40), type 1 diabetes (n = 98), type 2 diabetes (n = 167), and nondiabetic controls (n = 98). The DG9-glycan © was markedly lower in HNF1A-MODY than in controls or other diabetes subtypes, offered good discrimination between HNF1A-MODY and both type 1 and type 2 diabetes (C statistic >= 0.90), and enabled us to detect three previously undetected HNF1A mutations in patients with diabetes. In conclusion, glycan profiles are altered substantially in HNF1A-MODY, and the DG9-glycan © has potential clinical value as a diagnostic biomarker of HNF1A dysfunction. Diabetes 62:1329-1337, 2013
13.	Yusuf S, Hawken S, Ounpuu S, Bautista L, Franzosi MG, Commerford P, Lang CC, Rumboldt Z, Onen CL, Lisheng L, Tanomsup S, Wangai P Jr, Razak F, Sharma AM, Anand SS; INTERHEART Study Investigators. Obesity and the risk of myocardial infarction in 27,000 participants from 52 countries: a case-control study. Lancet. 2005 Nov 5;366(9497):1640-9. doi: 10.1016/S0140-6736(05)67663-5.	Background Obesity is a major risk factor for cardiovascular disease, but the most predictive measure for different ethnic populations is not clear. We aimed to assess whether markers of obesity, especially waist-to-hip ratio, would be stronger indicators of myocardial infarction than body-mass © (BMI), the conventional measure.<br>Methods We did a standardised case-control study of acute myocardial infarction with 27 098 participants in 52 countries (12461 cases and 14637 controls) representing several major ethnic groups. We assessed the relation between BMI, waist and hip circumferences, and waist-to-hip ratio to myocardial infarction overall and for each group.<br>Findings BMI showed a modest and graded association with myocardial infarction (OR 1.44, 95% CI 1.32-1.57 top quintile vs bottom quintile before adjustment), which was substantially reduced after adjustment for waist-to-hip ratio (1.12, 1.03-1.22), and non-significant after adjustment for other risk factors (0.98, 0.88-1.09). For waist-to-hip ratio, the odds ratios for every successive quintile were significantly greater than that of the previous one (2nd quintile: 1.15, 1.05-1.26; 3rd quintile: 1.39; 1.28-1.52; 4th quintile: 1.90, 1.74-2.07; and 5th quintiles: 2.52, 2.31-2.74 [adjusted for age, ©, region, and smoking]). Waist (adjusted OR 1.77; 1.59-1.97) and hip (0.73; 0.66-0.80) circumferences were both highly significant after adjustment for BMI (p<0.0001 top vs bottom quintiles). Waist-to-hip ratio and waist and hip circumferences were closely (p<0.0001) associated with risk of myocardial infarction even after adjustment for other risk factors (Ors for top quintile vs lowest quintiles were 1.75, 1.33, and 0.76, respectively). The population-attributable risks of myocardial infarction for increased waist-to-hip ratio in the top two quintiles was 24.3% (95% CI 22.5-26.2) compared with only 7.7% (6.0-10.0) for the top two quintiles of BMI.

		Interpretation Waist-to-hip ratio shows a graded and highly significant association with myocardial infarction risk worldwide. Redefinition of obesity based on waist-to-hip ratio instead of BMI increases the estimate of myocardial infarction attributable to obesity in most ethnic groups.
	**Gastroenterology & Hepatology**	
14.	Nyssen OP, Bordin D, Tepes B, Pérez-Aisa Á, Vaira D, Caldas M, Bujanda L, Castro-Fernandez M, Lerang F, Leja M, Rodrigo L, Rokkas T, Kupcinskas L, Pérez-Lasala J, Jonaitis L, Shvets O, Gasbarrini A, Simsek H, Axon ATR, Buzás G, Machado JC, Niv Y, Boyanova L, Goldis A, Lamy V, Tonkic A, Przytulski K, Beglinger C, Venerito M, Bytzer P, Capelle L, Milosavljević T, Milivojevic V, Veijola L, Molina-Infante J, Vologzhanina L, Fadeenko G, Ariño I, Fiorini G, Garre A, Garrido J, F Pérez C, Puig I, Heluwaert F, Megraud F, O'Morain C, Gisbert JP; Hp-EuReg Investigators. European Registry on Helicobacter pylori management (Hp-EuReg): patterns and trends in first-line empirical eradication prescription and outcomes of 5 years and 21 533 patients. Gut. 2021 Jan;70(1):40-54. doi: 10.1136/gutjnl-2020-321372. Epub 2020 Sep 21.	Objective The best approach for Helicobacter pylori management remains unclear. An audit process is essential to ensure clinical practice is aligned with best standards of care.<br>Design International multicentre prospective non-interventional registry starting in 2013 aimed to evaluate the decisions and outcomes in H. pylori management by European gastroenterologists. Patients were registered in an e-CRF by AEG-REDCap. Variables included demographics, previous eradication attempts, prescribed treatment, adverse events and outcomes. Data monitoring was performed to ensure data quality. Time-trend and geographical analyses were performed.<br>Results 30 394 patients from 27 European countries were evaluated and 21 533 (78%) first-line empirical H. pylori treatments were included for analysis. Pretreatment resistance rates were 23% to clarithromycin, 32% to metronidazole and 13% to both. Triple therapy with amoxicillin and clarithromycin was most commonly prescribed (39%), achieving 81.5% modified intention-to-treat eradication rate. Over 90% eradication was obtained only with 10-day bismuth quadruple or 14-day concomitant treatments. Longer treatment duration, higher acid inhibition and compliance were associated with higher eradication rates. Time-trend analysis showed a region-dependent shift in prescriptions including abandoning triple therapies, using higher acid-inhibition and longer treatments, which was associated with an overall effectiveness increase (84%-90%).<br>Conclusion Management of H. pylori infection by European gastroenterologists is heterogeneous, suboptimal and discrepant with current recommendations. Only quadruple therapies lasting at least 10 days are able to achieve over 90% eradication rates. European recommendations are being slowly and heterogeneously incorporated into routine clinical practice, which was associated with a corresponding increase in effectiveness.
15.	Megraud F, Coenen S, Versporten A, Kist M, Lopez-Brea M, Hirschl AM, Andersen LP, Goossens H, Glupczynski Y; Study Group participants. Helicobacter pylori resistance to antibiotics in Europe and its relationship to antibiotic	Objective Resistance to antibiotics is the major cause of treatment failure of Helicobacter pylori infection. A study was conducted to assess prospectively the antibacterial resistance rates of H pylori in Europe and to study the link between outpatient antibiotic use and resistance levels in different countries.<br>Design Primary antibiotic resistance rates of H pylori were determined from April 2008 to June 2009 in 18 European countries. Data on yearly and cumulative use over several years

	consumption. Gut. 2013 Jan;62(1):34-42. doi: 10.1136/gutjnl-2012-302254. Epub 2012 May 12. (member of Study Group: M Tonkić)	of systemic antibacterial agents in ambulatory care for the period 2001-8 were expressed in Defined Daily Doses (DDD) per 1000 inhabitants per day. The fit of models and the degree of ecological association between antibiotic use and resistance data were assessed using generalised linear mixed models.<br>Results Of 2204 patients included, H pylori resistance rates for adults were 17.5% for clarithromycin, 14.1% for levofloxacin and 34.9% for metronidazole, and were significantly higher for clarithromycin and levofloxacin in Western/Central and Southern Europe (>20%) than in Northern European countries (<10%). Model fit improved for each additional year of antibiotic use accumulated, but the best fit was obtained for 2005. A significant association was found between outpatient quinolone use and the proportion of levofloxacin resistance (p = 0.0013) and between the use of long-acting macrolides only and clarithromycin resistance (p = 0.036).<br>Conclusion In many countries the high rate of clarithromycin resistance no longer allows its empirical use in standard anti-H pylori regimens. The knowledge of outpatient antibiotic consumption may provide a simple tool to predict the susceptibility of H pylori to quinolones and to macrolides and to adapt the treatment strategies.
16.	Terzić J, Grivennikov S, Karin E, Karin M. Inflammation and colon cancer. Gastroenterology. 2010 Jun;138(6):2101-2114.e5. doi: 10.1053/j.gastro.2010.01.058..	The connection between inflammation and tumorigenesis is well-established and in the last decade has received a great deal of supporting evidence from genetic, pharmacological, and epidemiological data. Inflammatory bowel disease 11 san important risk factor for the development of colon cancer. Inflammation is also likely to be involved with other forms of sporadic as well as heritable colon cancer. The molecular mechanisms by which inflammation promotes cancer development are still being uncovered and could differ between colitis-associated and other forms of colorectal cancer. Recent work has elucidated the role of distinct immune cells, cytokines, and other immune mediators in virtually all steps of colon tumorigenesis, including initiation, promotion, progression, and metastasis. These mechanisms, as well as new approaches to prevention and therapy, are discussed in this review.
17.	Kapitanović S, Radosević S, Kapitanović M, Andelinović S, Ferencić Z, Tavassoli M, Primorać D, Sonicki Z, Spaventi S, Pavelic K, Spaventi R. The expression of p185(HER-2/neu) correlates with the stage of disease and survival in colorectal cancer. Gastroenterology. 1997 Apr;112(4):1103-13. doi: 10.1016/s0016-5085(97)70120-3.	Background & Aims: HER-2/neu oncogene encodes a transmembrane tyrosine kinase receptor that is amplified and/or overexpressed predominantly in adenocarcinomas, This phenomenon has been most intensively studied in breast carcinoma where its amplification and overexpression correlate with the overall course of disease and poor prognosis, This study was designed to investigate HER-2/neu gene expression in benign and malignant colorectal lesions and to evaluate its prognostic importance in colorectal cancer, Methods: Two hundred twenty-one samples of normal colon, benign lesions, and colorectal adenocarcinomas were studied for expression of HER-2/neu oncoprotein,

		Immunohistochemical staining of formalin-fixed, paraffin-embedded tissue sections of primary tumor and lymph nodes was performed. Immunoprecipitation followed by Western blotting of freshly frozen samples of the same tumors were also performed, Results: Normal colon mucosa, benign lesions, and adenocarcinomas clearly differed in the expression levels and histological distribution of p185(HER-2/neu). Normal mucosa was mostly negative, but significant number of benign lesions and adenocarcinomas overexpressed HER-2/neu protein, Adenocarcinomas were significantly more positive than benign lesions. The results show significant correlation with the epithelial abnormality degree and clinical parameters including Dukes' classification and relapse-free and postoperative survival period, Conclusions: The p185(HER-2/neu) rate expression could serve as an independent prognostic factor in patients with p185(HER-2)/(neu)-positive colorectal malignancies.
	**Geriatrics & Gerontology**	
18.	Palada V, Terzić J, Mazzulli J, Bwala G, Hagenah J, Peterlin B, Hung AY, Klein C, Krainc D. Histamine N-methyltransferase Thr105Ile polymorphism is associated with Parkinson's disease. Neurobiol Aging. 2012 Apr;33(4):836.e1-3. doi: 10.1016/j.neurobiolaging.2011.06.015. Epub 2011 Jul 27.	Histamine is a central neurotransmitter degraded by histamine-N-methyltransferase (HNMT). Several abnormalities in the histaminergic system were found in patients with Parkinson's disease (PD), thus we tested the possible association of a Thr105Ile functional polymorphism in HNMT with PD. A total of 913 patients with PD and 958 controls were genotyped using a TaqMan RT-PCR Genotyping Assay (Foster City, California, USA). Lower frequency of HNMT Ile105 allele that is associated with decreased enzymatic activity was found in patients compared with controls (chi(2) = 11.65; p = 0.0006). We performed meta-analysis to confirm the association of Thr105Ile functional polymorphism with PD. Our results indicate that lower HNMT activity plays a role in the pathogenesis of PD. © 2012 Elsevier Inc. All rights reserved.
	**Health Care Sciences & Services**	
19.	Nørgaard B, Briel M, Chrysostomou S, Ristic Medic D, Buttigieg SC, Kiisk E, Puljak L, Bala M, Pericic TP, Lesniak W, Zając J, Lund H, Pieper D. A systematic review of meta-research studies finds substantial methodological heterogeneity in citation analyses to monitor evidence-based research. J Clin Epidemiol. 2022 Oct;150:126-141. doi: 10.1016/j.jclinepi.2022.06.021.	Objectives: This systematic review aimed to identify the characteristics and application of citation analyses in evaluating the justification, design, and placement of the research results of clinical health studies in the context of earlier similar studies.<br>Study Design and Setting: We searched MEDLINE (Ovid), Embase (Ovid), and the Cochrane Methodology Register for meta-research studies. We included meta-research studies assessing whether researchers used earlier similar studies and/or systematic reviews of such studies to inform the justification or design of a new study, whether researchers used systematic reviews to inform the interpretation of new results, and meta-research studies assessing whether redundant studies were published within a specific area. The results are presented as a narrative synthesis.<br>Results: A total of 27 studies were included. How authors of citation analyses define their

		outcomes appears rather arbitrary, as does how the reference of a landmark review or adherence to reporting guidelines was expected to contribute to the initiation, justification, design, or contextualization of relevant clinical trials.<br>Conclusion: Continued and improved efforts to promote evidence-based research are needed, including clearly defined and justified outcomes in meta-research studies to monitor the implementation of an evidence-based approach. © 2022 The Authors. Published by Elsevier Inc. This 13 san open access article under the CC BY license (http://creativecommons.org/licenses/by/4.0/).
20.	Puljak L, Ramic I, Arriola Naharro C, Brezova J, Lin YC, Surdila AA, Tomajkova E, Farias Medeiros I, Nikolovska M, Poklepovic Pericic T, Barcot O, Suarez Salvado M. Cochrane risk of bias tool was used inadequately in the majority of non-Cochrane systematic reviews. J Clin Epidemiol. 2020 Jul;123:114-119. doi: 10.1016/j.jclinepi.2020.03.019.	Objectives: To analyze how many non-Cochrane systematic reviews (NCSRs) used Cochrane's risk of bias (RoB) tool, domains they used, and whether judgments and comments about RoB were in line with Cochrane Handbook.<br>Methods: This was a methodological (research-on-research) study. We retrieved NCSRs from PubMed, extracted information about methods used for RoB assessment, and if they used 2011 Cochrane RoB tool, we analyzed their RoB methods and compared them with Cochrane Handbook guidance.<br>Results: We included 508 NCSRs; 431 (85%) reported they analyzed RoB, and 269 (53%) used Cochrane RoB tool. Only 16 of those 269 (5.9%) reported both a judgment and a supporting comment in the Cochrane RoB table in the manuscript (N = 4) or in a supplementary file (N = 12). Fifteen reviews, with 158 included trials, used judgments low/high/unclear; 41% of analyzed available judgments were inadequate, either because judgment was not in line with comment or comment was missing.<br>Conclusions: Most NCSRs use Cochrane RoB tool to assess RoB, but most of them reported it incompletely, with high prevalence of inadequate judgments. Authors, editors, and peer-reviewers should make an effort to improve completeness and adequacy of Cochrane RoB assessment in non-Cochrane reviews.
21.	Saric L, Dosenovic S, Saldanha IJ, Jelicic Kadic A, Puljak L. Conference abstracts describing systematic reviews on pain were selectively published, not reliable, and poorly reported. J Clin Epidemiol. 2020 Jan;117:1-8. doi: 10.1016/j.jclinepi.2019.09.011.	Objective: The objective of the study was to determine the reporting quality of systematic review (SR) abstracts presented at World Congresses on Pain (WCPs) and to quantify agreement in results presented in those abstracts with their corresponding full-length publications.<br>Study Design and Setting: We screened abstracts of five WCPs held from 2008 to 2016 to find abstracts describing SRs. Two authors searched for corresponding full publications using PubMed and Google Scholar in April 2018. Methods and outcomes extracted from abstracts were compared with their corresponding full publications. The reporting quality of abstracts was evaluated against the PRISMA for Abstracts (PRISMA-A) checklist.<br>Results: We identified 143 conference abstracts describing SRs. Of these, 90 (63%) were

		published as full-length articles in peer-reviewed journals by April 2018, with a median time from conference presentation to publication of 5 months (interquartile range: -0.25 to 14 months). Among 79 abstract-publication pairs evaluable for discordance, there was some form of discordance in 40% of pairs. Qualitative discordance (different direction of the effect) was found in 13 analyzed pairs (16%). The median adherence by abstracts to each PRISMA-A checklist item was 33% (interquartile range: 29% to 42%).<br>Conclusion: Conference abstracts of pain SRs are selectively published, not reliable, and poorly reported. © 2019 Elsevier Inc. All rights reserved.
22.	Jurić D, Bolić A, Pranić S, Marušić A. Drug-drug interaction trials incompletely described drug interventions in ClinicalTrials.gov and published articles: an observational study. J Clin Epidemiol. 2020 Jan;117:126-137. doi: 10.1016/j.jclinepi.2019.10.002.	Objectives: The aim of the study was to evaluate the completeness of intervention description in ClinicalTrials.gov and corresponding journal articles for registered and published drug-drug interaction (DDI) trials because complete and transparent description of interventions is particularly important for DDI.<br>Study Design and Setting: Observational study of completed interventional trials on DDIs with up to two drugs within the Intervention registration element in ClinicalTrials.gov until October 2015. We used the Template for Intervention Description and Replication items to assess the quality of intervention description in both ClinicalTrials.gov Descriptive Information section and matching publications. Corresponding articles were identified in March 2019.<br>Results: The description of 1,180 drug interventions registered for 642 DDI trials mostly lacked information on the intervention provider (99.7%), adherence strategies (99.2%), procedure (83.8%), location (71.3%), and dosage form (60.7%). Generic name (82.5%), dose (70.8%), and duration of administration (65.6%) were most frequently reported. Among 51 trials that had data reported both in ClinicalTrials.gov and publication, 60.8% were in phase 1. Less than half of 96 interventions had clear and matching description of dosage form, procedure, and route of administration in both sources.<br>Conclusion: DDI trials did not sufficiently report components required for complete intervention description. Further improvements in ClinicalTrials.gov registration requirements, including phase 1 trials, and more stringent publishing requirements for essential data on drug interventions, are needed to prevent patient risk in clinical practice regarding concomitant medication use. © 2019 Elsevier Inc. All rights reserved.
23.	Meneses-Echavez JF, Bidonde J, Yepes-Nuñez JJ, Poklepović Peričić T, Puljak L, Bala MM, Storman D, Swierz MJ, Zając J, Montesinos-Guevara C, Zhang Y, Chavez Guapo N, Schünemann H, Flottorp S, Alonso-Coello P. Evidence to decision frameworks enabled	Objective: The aim of this study is to identify and describe the processes suggested for the formulation of healthcare recommendations in healthcare guidelines available in guidance documents.<br>Methods: We searched international databases in May 2020 to retrieve guidance documents published by organizations dedicated to guideline development. Pairs of

	structured and explicit development of healthcare recommendations. J Clin Epidemiol. 2022 Oct;150:51-62. doi: 10.1016/j.jclinepi.2022.06.004.	researchers independently selected and extracted data about the characteristics of the guidance document, including explicit or implicit recommendation-related criteria and processes considered, as well as the use of evidence to decision (EtD) frameworks.<br>Results: We included 68 guidance documents. Most organizations reported a system for grading the strength of recommendations (88%), half of them being the GRADE (Grading of Recommendations Assessment, Development, and Evaluation) approach. Two out of three guidance documents (66%) proposed the use of a framework to guide the EtD process. The GRADE-EtD framework was the most often reported framework (19 organizations, 42%), whereas 20 organizations (44%) proposed their own multicriteria frameworks. Using any EtD framework was related with a more comprehensive set of recommendation-related criteria compared to no framework, especially for criteria like values, equity, and acceptability.<br>Conclusion: Although limited, the use of EtD frameworks was associated with the inclusion of relevant recommendation criteria. Among the EtD structured frameworks, the GRADE-EtD framework offers the most comprehensive perspective for evidence-informed decision-making processes. © 2022 The Author(s). Published by Elsevier Inc. This 15 san open access article under the CC BY license (http://creativecommons.org/licenses/by/4.0/).
24.	Hamel C, Michaud A, Thuku M, Affengruber L, Skidmore B, Nussbaumer-Streit B, Stevens A, Garritty C. Few evaluative studies exist examining rapid review methodology across stages of conduct: a systematic scoping review. J Clin Epidemiol. 2020 Oct;126:131-140. doi: 10.1016/j.jclinepi.2020.06.027.	Objectives: The objective is to identify studies that have assessed methodological shortcuts for undertaking rapid reviews (RRs) and mapping these to review conduct stages and Methodological Expectations of Cochrane Intervention Reviews (MECIR) guidance.<br>Study Design and Setting: We conducted a systematic scoping review. We searched multiple databases (e.g., MEDLINE, Embase), which were supplemented by grey literature searching. Methods were defined a priori in a published protocol.<br>Results: Out of 1,873 records, 90 publications were divided into four RR categories: formal evaluation (n = 14), development, which included four subcategories (n = 65), comparison (n = 2), and applying reporting guidelines/critical appraisal tools (n = 3), and a systematic review surrogate category (n = 6). Four formal evaluation studies were composite evaluations, including more than one shortcut simultaneously. The remaining 10 studies evaluated viable (e.g., including English-only publications) and unviable (e.g., single-reviewer screening) shortcuts, covering five key dimensions and five 'other' (e.g., involving stakeholders) considerations while conducting a review. Because of complexities around shortcuts evaluated, only a cursory mapping to MECIR criteria was possible.<br>Conclusion: Some methods shortcuts may be valid in the context of RRs, but limitations in the studies may limit their applicability. The results will serve to inform discussions within

		Cochrane regarding possible future implementation of RRs. © 2020 Elsevier Inc. All rights reserved.
25.	Könsgen N, Barcot O, Heß S, Puljak L, Goossen K, Rombey T, Pieper D. Inter-review agreement of risk-of-bias judgments varied in Cochrane reviews. J Clin Epidemiol. 2020 Apr;120:25-32. doi: 10.1016/j.jclinepi.2019.12.016.	Objectives: The objective of the study was to measure the level of agreement between Cochrane reviews of overlapping randomized controlled trials (RCTs) regarding risk-of-bias (RoB) judgments.<br>Study Design and Setting: On November 5, 2017, the Cochrane Database of Systematic Reviews was searched for Cochrane reviews on tobacco. Reviews that included overlapping RCTs were included. RoB judgments were extracted from RoB tables using automated data scraping with manual verification and adjustments. Agreement between the reviews was calculated using Conger's generalized kappa coefficient (kappa) and raw agreement (a).<br>Results: We included 53 Cochrane reviews of 376 RCTs. For the RoB domain „random sequence generation,“ the level of agreement between the reviews was substantial with kappa = 0.63 (95% confidence interval: 0.56 to 0.71; a = 0.80). There was slight-to-moderate agreement between the reviews regarding the domains „allocation concealment“: kappa = 0.51 (0.41 to 0.61), a = 0.75; „blinding“: kappa = 0.19 (0.02 to 0.37), a = 0.52; „blinding of outcome assessment“: kappa = 0.43 (0.14 to 0.72) a = 0.67; and „incomplete outcome data“: kappa = 0.15 (-0.03 to 0.32), a = 0.64. For „blinding of participants and personnel“ and „selective reporting“, kappa could not be calculated. The raw agreement was 0.40 and 0.42, respectively.<br>Conclusion: The level of agreement between Cochrane reviews regarding RoB judgments ranged from slight to substantial depending on the RoB domain. Further investigations regarding reasons for variation and interventions to improve agreement are needed. (C) 2019 Elsevier Inc. All rights reserved.
26.	Gabelica M, Bojčić R, Puljak L. Many researchers were not compliant with their published data sharing statement: a mixed-methods study. J Clin Epidemiol. 2022 Oct;150:33-41. doi: 10.1016/j.jclinepi.2022.05.019.	Objectives: The objective of the study was to analyze researchers' compliance with their data availability statement (DAS) from manuscripts published in open-access journals with the mandatory DAS.<br>Study Design and Setting: We analyzed all articles from 333 open-access journals published during January 2019 by BioMed Central. We categorized types of the DAS. We surveyed corresponding authors who wrote in the DAS that they would share the data. Consent to participate in the study was sought for all included manuscripts. After accessing raw data sets, we checked whether data were available in a way that enabled reanalysis.<br>Results: Of 3556 analyzed articles, 3416 contained the DAS. The most frequent DAS category (42%) indicated that the data sets are available on reasonable request. Among 1792 manuscripts in which the DAS indicated that authors are willing to share their data,

		1669 (93%) authors either did not respond or declined to share their data with us. Among 254 (14%) of 1792 authors who responded to our query for data sharing, only 123 (6.8%) provided the requested data.<br>Conclusion: Even when authors indicate in their manuscript that they will share data upon request, the compliance rate is the same as for authors who do not provide the DAS, suggesting that the DAS may not be sufficient to ensure data sharing. © 2022 Elsevier Inc. All rights reserved.
27.	Sharp MK, Glonti K, Hren D. Online survey about the STROBE statement highlighted diverging views about its content, purpose, and value. J Clin Epidemiol. 2020 Jul;123:100-106. doi: 10.1016/j.jclinepi.2020.03.025.	Background and objective: The endorsement rates of The Strengthening the Reporting of Observational studies in Epidemiology<br>(STROBE) Statement are low and little is known about authors' opinions about this reporting guideline. We conducted an online survey<br>with observational study authors on attitude toward and experiences with the STROBE Statement with the aim of understanding how to<br>effectively implement STROBE.<br>Methods: A thematic analysis on the responses to an open-ended question was conducted using inductive coding. Two coders classified<br>responses independently into themes using a codebook. The inter-rater agreement ranged from 87.7 to 99.9%.<br>Results: 15% (n 5 150) of survey participants (n 5 1,015) shared perceptions and insights on STROBE. We established four themes: 1)<br>perceptions of the checklist, 2) academic confidence, 3) use in education and training, and 4) journal endorsement and use in peer review.<br>Views were diverse and revealed multiple misunderstandings about the checklist's purpose and content, and lack of incentives for its use.<br>Conclusions: Better communication efforts are needed when disseminating STROBE and other reporting guidelines. These should<br>focus on content, education for early career researchers, and encouragement of critical self-reflection on one's own work. In addition, results<br>emphasized the need for better incentive and enforcement mechanisms.
28.	Babic A, Vuka I, Saric F, Proloscic I, Slapnicar E, Cavar J, Poklepovic Pericic T, Pieper D, Puljak L. Overall bias methods and their use in sensitivity analysis of Cochrane reviews were not consistent. J Clin Epidemiol. 2020 Mar;119:57-64. doi: 10.1016/j.jclinepi.2019.11.008.	Objective: The objective of the study was to analyze methods of assessing „overall bias" in Cochrane reviews of interventions published in the Cochrane Database of Systematic Reviews and sensitivity analyses related to overall risk of bias (RoB).<br>Study Design and Setting: From Cochrane reviews published within 3 years, from July 2015 to June 2018, we extracted data regarding methods of judging overall bias for a single trial, as well as details regarding methods used in frequency of RoB in sensitivity analyses.

		Results: Of the 1,452 analyzed Cochrane reviews, 409 mentioned assessment of overall RoB on a study level. In 107 reviews, authors clearly specified key domains that determined the overall RoB, whereas in the remaining reviews, assessment of overall bias was not in line with the Cochrane Handbook. Among 268 Cochrane reviews that had any RoB-related sensitivity analysis, in 56 (21%) reviews, the authors reported a significant change for at least one outcome compared with the initial analysis.<br>Conclusion: Highly heterogeneous approaches to summarizing overall RoB on a study level and using RoB for sensitivity analyses may yield inconsistent and incomparable results across Cochrane reviews. © 2019 Elsevier Inc. All rights reserved.
29.	Roguljić M, Šimunović D, Poklepović Peričić T, Viđak M, Utrobičić A, Marušić M, Marušić A. Publishing Identifiable Patient Photographs in Scientific Journals: Scoping Review of Policies and Practices. J Med © Res. 2022 Aug 31;24(8):e37594. doi: 10.2196/37594.	Background: Publishing identifiable patient data in scientific journals may jeopardize patient privacy and confidentiality if best ethical practices are not followed. Current journal practices show considerable diversity in the publication of identifiable patient photographs, and different stakeholders may have different opinions of and practices in publishing patient photographs.<br>Objective: This scoping review aimed to identify existing evidence and map knowledge gaps in medical research on the policies and practices of publishing identifiable photographs in scientific articles.<br>Methods: We performed a comprehensive search of the Cochrane Central Register of Controlled Trials, Cochrane Database of Systematic Reviews, CINAHL with Full Text, Database of Abstracts of Reviews of Effects, Ovid MEDLINE, and Scopus. The Open Science Framework, PROSPERO, BASE, Google Scholar, OpenGrey, ClinicalTrials.gov, the Campbell Collaboration Library, and Science.gov were also searched.<br>Results: After screening the initial 15,949 titles and abstracts, 98 (0.61%) publications were assessed for eligibility at the full-text level, and 30 (0.19%) publications were included in this review. The studies were published between 1994 and 2020; most had a cross-sectional design and were published in journals covering different medical disciplines. We identified 3 main topics. The first included ethical aspects of the use of facial photographs in publications. In different clinical settings, the consent process was not conducted properly, and health professionals did not recognize the importance of obtaining written patient consent for taking and using patient medical photographs. They often considered verbal consent sufficient or even used the photographs without consent. The second topic included studies that investigated the practices and use of medical photography in publishing. Both patients and doctors asked for confidential storage and maintenance of medical photographs. Patients preferred to be photographed by their physicians using an institutional camera and preferred nonidentifiable medical photographs not only for

		publication but also in general. Conventional methods of deidentification of facial photographs concealing the eye area were recognized as unsuccessful in protecting patient privacy. The third topic emerged from studies investigating medical photography in journal articles. These studies showed great diversity in publishing practices regarding consent for publication of medical photographs. Journal policies regarding the consent process and consent forms were insufficient, and existing ethical professional guidelines were not fully implemented in actual practices. Patients' photographs from open-access medical journals were found on public web-based platforms.<br>Conclusions: This scoping review showed a diversity of practices in publishing identifiable patient photographs and an unsatisfactory level of knowledge of this issue among different stakeholders despite existing standards. Emerging issues include the availability of patients' photographs from open-access journals or preprints in the digital environment. There is a need to improve standards and processes to obtain proper consent to fully protect the privacy of patients in published articles.
30.	Vucic K, Jelicic Kadic A, Puljak L. Survey of Cochrane protocols found methods for data extraction from figures not mentioned or unclear. J Clin Epidemiol. 2015 Oct;68(10):1161-4. doi: 10.1016/j.jclinepi.2014.11.016.	Objectives: To analyze whether protocols of Cochrane systematic reviews address data extraction from figures in included trials.<br>Study Design and Setting: Protocols of Cochrane systematic reviews published between May 2013 and May 2014 were screened by two authors independently, and the following data were collected: date of protocol publication, country of authors' origin, number of authors, number of affiliated institutions, Cochrane review group, whether the protocol contains description about data extraction from figures, method of data extraction from figures, and literature reference for a method of data extraction from figures.<br>Results: Among 589 protocols, 33 (5.6%) mentioned data extraction from figures in Methods section. Only one protocol specified that computer software will be used for data extraction from figures, one specifically indicated that data from figures will not be used, few stated estimation or approximation, whereas others did not provide any description of methodology for data extraction from figures.<br>Conclusion: Very few protocols of Cochrane systematic reviews mention data extraction from figures, and even when mentioned, methods for data extraction are unclear. Methodology for data extraction from figures should be incorporated into the Cochrane Handbook and new methodological standards for Cochrane systematic reviews. © 2015 Elsevier Inc. All rights reserved.
31.	Wang X, Chen Y, Akl EA, Tokalić R, Marušić A, Qaseem A, Falck-Ytter Y, Lee MS, Siedler M, Barber SL, Zhang M, Chan ESY, Estill J, Kwong JSW, Okumura	Background: Public or patient versions of guidelines (PVGs) are derivative documents that „translate“ recommendations and their rationale from clinical guidelines for health professionals into a more easily understandable and usable format for patients and the

	A, Zhou Q, Yang K, Norris SL; RIGHT working group. The reporting checklist for public versions of guidelines: RIGHT-PVG. Implement Sci. 2021 Jan 11;16(1):10. doi: 10.1186/s13012-020-01066-z.	public. PVGs from different groups and organizations vary considerably in terms of quality of their reporting. In order to address this issue, we aimed to develop a reporting checklist for developers of PVGs and other potential users.<br>Methods: First, we collected a list of potential items through reviewing a sample of PVGs, existing guidance for developing and reporting PVGs or other similar evidence-based patient tools, as well as qualitative studies on original studies of patients' needs about the content and/or reporting of information in PVGs or similar evidence-based patient tools. Second, we conducted a two-round Delphi consultation to determine the level of consensus on the items to be included in the final reporting checklist. Third, we invited two external reviewers to provide comments on the checklist.<br>Results: We generated the initial list of 45 reporting items based on a review of a sample of 30 PVGs, four PVG guidance documents, and 46 relevant studies. After the two-round Delphi consultation, we formed a checklist of 17 items grouped under 12 topics for reporting PVGs.<br>Conclusion: The RIGHT-PVG reporting checklist 20 san20er20up international consensus on the important criteria for reporting PVGs.
32.	Krnic Martinic M, Čivljak M, Marušić A, Sapunar D, Poklepović Peričić T, Buljan I, Tokalić R, Mališa S, Neuberg M, Ivanišević K, Aranza D, Skitarelić N, Zoranić S, Mikšić Š, Čavić D, Puljak L. Web-Based Educational Intervention to Improve Knowledge of Systematic Reviews Among Health Science Professionals: Randomized Controlled Trial. J Med © Res. 2022 Aug 25;24(8):e37000. doi: 10.2196/37000.	Background: Lack of knowledge of systematic reviews (SRs) could prevent individual health care professionals from using SRs as a source of information in their clinical practice or discourage them from participating in such research.<br>Objective: In this randomized controlled trial, we evaluated the effect of a short web-based educational intervention on short-term knowledge of SRs.<br>Methods: Eligible participants were 871 Master's students of university health sciences studies in Croatia; 589 (67.6%) students who agreed to participate in the trial were randomized using a computer program into 2 groups. Intervention group A (294/589, 49.9%) received a short web-based educational intervention about SR methodology, and intervention group B (295/589, 50.1%) was presented with the PRISMA (Preferred Reporting Items for Systematic Reviews and Meta-Analyses) checklist. The participants' knowledge of SRs was assessed before and after the intervention. The participants could not be blinded because of the nature of the intervention. The primary outcome was the difference in the percentage of correct answers about SR methodology per participant between the groups after the intervention, expressed as relative risk and 95% CI.<br>Results: Results from 162 and 165 participants in the educational intervention and PRISMA checklist groups, respectively, were available for analysis. Most of them (educational intervention group: 130/162, 80.2%; PRISMA checklist group: 131/165, 79.4%) were employed as health care professionals in addition to being health sciences students. After

		the intervention, the educational intervention group had 23% (relative risk percentage) more correct answers in the postintervention questionnaire than the PRISMA checklist group (relative risk=1.23, 95% CI 1.17-1.29).<br>Conclusions: A short web-based educational intervention about SRs 21 san effective tool for short-term improvement of knowledge of SRs among health care studies students, most of whom were also employed as health care professionals. Further studies are needed to explore the long-term effects of the tested education.
	**Infectious Diseases**	
33.	Jerončić A, Nonković D, Vrbatović A, Hrabar J, Bušelić I, Martínez-Sernández V, Lojo Rocamonde SA, Ubeira FM, Jaman S, Jeličić EČ, Amati M, Gomez Morales MA, Lukšić B, Mladineo I. Anisakis Sensitization in the Croatian fish processing workers: Behavioral instead of occupational risk factors? PloS Negl Trop Dis. 2020 Jan 27;14(1):e0008038. doi: 10.1371/journal.pntd.0008038.	We undertook the first study systematically evaluating the risk of Anisakis-sensitization in Croatian fish-processing workers and potential genetic susceptibility to anisakiasis. AntiAnisakis IgE seroprevalence and risk factors for 600 employees of Croatian fish processing facilities and 466 blood donor controls, were assessed by indirect ELISA targeted with: recombinant Ani s 1 and Ani s 7 allergens, an Anisakis crude extract, the commercial ImmunoCAP kit, and questionnaires. Genetic susceptibility to anisakiasis was evaluated by genotypisation of human leukocytes alleles (HLA). Anti-Anisakis seropositive and a fraction of negative subjects were also assessed by ELISA and Western Blot (WB) for IgG seroprevalence to Trichinella spp. Overall, the observed anti-Anisakis seroprevalence inferred by indirect ELISA was significantly higher in fish processing workers (1.8%, 95% CI 0.9-3.3%) compared to the controls (0%, 0-0.8%). Seven out of 11 Ani s 1 and Ani s 7-positives and none of selected 65 negative sera, tested positive on whole-Anisakis extract (ImmunoCAP), whereas Anisakis crude extract ELISA detected 3.9% (2.4-6.0%) seropositives in fish processing workers, three (14%) of which showed IgE reactivity to milk proteins. The highest risk associated with Anisakis-sensitization among workers was fishing in the free time, rather than any of attributes related to the occupational exposure. Although no association was observed between anti-Anisakis seropositivity and wearing gloves or protective goggles, the majority of workers (92%) wore protective gloves, minimizing the risk for Anisakis sensitization via skin contact. Six HLA alleles within DRB1 gene were significantly associated with seropositivity under dominant, allelic or recessive models. All sera confirmed negative for anti-Trichinella spp. IgG. The study exhaustively covered almost all marine fish processing workers in Croatia, reflecting real-time Anisakis sensitization status within the industry, already under the influence of wide array of allergens.
34.	Nair H, Brooks WA, Katz M, Roca A, Berkley JA, Madhi SA, Simmerman JM, Gordon A, Sato M, Howie S, Krishnan A, Ope M, Lindblade KA, Carosone-Link P,	Background The global burden of disease attributable to seasonal influenza virus in children is unknown. We aimed to estimate the global incidence of and mortality from lower respiratory infections associated with influenza in children younger than 5 years.

	Lucero M, Ochieng W, Kamimoto L, Dueger E, Bhat N, Vong S, Theodoratou E, Chittaganpitch M, Chimah O, Balmaseda A, Buchy P, Harris E, Evans V, Katayose M, Gaur B, O'Callaghan-Gordo C, Goswami D, Arvelo W, Venter M, Briese T, Tokarz R, Widdowson MA, Mounts AW, Breiman RF, Feikin DR, Klugman KP, Olsen SJ, Gessner BD, Wright PF, Rudan I, Broor S, Simões EA, Campbell H. Global burden of respiratory infections due to seasonal influenza in young children: a systematic review and meta-analysis. Lancet. 2011 Dec 3;378(9807):1917-30. doi: 10.1016/S0140-6736(11)61051-9.	Methods We estimated the incidence of influenza episodes, influenza-associated acute lower respiratory infections (ALRI), and influenza-associated severe ALRI in children younger than 5 years, stratified by age, with data from a systematic review of studies published between Jan 1, 1995, and Oct 31, 2010, and 16 unpublished population-based studies. We applied these incidence estimates to global population estimates for 2008 to calculate estimates for that year. We estimated possible bounds for influenza-associated ALRI mortality by combining incidence estimates with case fatality ratios from hospital-based reports and identifying studies with population-based data for influenza seasonality and monthly ALRI mortality.<br>Findings We identified 43 suitable studies, with data for around 8 million children. We estimated that, in 2008, 90 million (95% CI 49-162 million) new cases of influenza (data from nine studies), 20 million (13-32 million) cases of influenza-associated ALRI (13% of all cases of paediatric ALRI; data from six studies), and 1 million (1-2 million) cases of influenza-associated severe ALRI (7% of cases of all severe paediatric ALRI; data from 39 studies) occurred worldwide in children younger than 5 years. We estimated there were 28 000-111 500 deaths in children younger than 5 years attributable to influenza-associated ALRI in 2008, with 99% of these deaths occurring in developing countries. Incidence and mortality varied substantially from year to year in any one setting.<br>Interpretation Influenza is a common pathogen identified in children with ALRI and results in a substantial burden on health services worldwide. Sufficient data to precisely estimate the role of influenza in childhood mortality from ALRI are not available.
35.	Li Y, Wang X, Blau DM, Caballero MT, Feikin DR, Gill CJ, Madhi SA, Omer SB, Simões EAF, Campbell H, Pariente AB, Bardach D, Bassat Q, Casalegno JS, Chakhunashvili G, Crawford N, Danilenko D, Do LAH, Echavarria M, Gentile A, Gordon A, Heikkinen T, Huang QS, Jullien S, Krishnan A, Lopez EL, Markić J, Mira-Iglesias A, Moore HC, Moyes J, Mwananyanda L, Nokes DJ, Noordeen F, Obodai E, Palani N, Romero C, Salimi V, Satav A, Seo E, Shchomak Z, Singleton R, Stolyarov K, Stoszek SK, von Gottberg A, Wurzel D, Yoshida LM, Yung CF, Zar HJ; Respiratory Virus Global Epidemiology Network; Nair H; RESCEU investigators. Global, regional, and national disease burden estimates of acute lower respiratory	Background Respiratory syncytial virus (RSV) is the most common cause of acute lower respiratory infection in young children. We previously estimated that in 2015, 33.1 million episodes of RSV-associated acute lower respiratory infection occurred in children aged 0-60 months, resulting in a total of 118 200 deaths worldwide. Since then, several community surveillance studies have been done to obtain a more precise estimation of RSV associated community deaths. We aimed to update RSV-associated acute lower respiratory infection morbidity and mortality at global, regional, and national levels in children aged 0-60 months for 2019, with focus on overall mortality and narrower infant age groups that are targeted by RSV prophylactics in development.<br>Methods In this systematic analysis, we expanded our global RSV disease burden dataset by obtaining new data from an updated search for papers published between Jan 1, 2017, and Dec 31, 2020, from MEDLINE, Embase, Global Health, CINAHL, Web of Science, LILACS, OpenGrey, CNKI, Wanfang, and ChongqingVIP. We also included unpublished data from RSV GEN collaborators. Eligible studies reported data for children aged 0-60 months with

infections due to respiratory syncytial virus in children younger than 5 years in 2019: a systematic analysis. Lancet. 2022 May 28;399(10340):2047-2064. doi: 10.1016/S0140-6736(22)00478-0.	RSV as primary infection with acute lower respiratory infection in community settings, or acute lower respiratory infection necessitating hospital admission; reported data for at least 12 consecutive months, except for in-hospital case fatality ratio (CFR) or for where RSV seasonality is well-defined; and reported incidence rate, hospital admission rate, RSV positive proportion in acute lower respiratory infection hospital admission, or in-hospital CFR. Studies were excluded if case definition was not clearly defined or not consistently applied, RSV infection was not laboratory confirmed or based on serology alone, 23 san the report included fewer than 50 cases of acute lower respiratory infection. We applied a generalised linear mixed-effects model (GLMM) to estimate RSV-associated acute lower respiratory infection incidence, hospital admission, and in-hospital mortality both globally and regionally (by country development status and by World Bank Income Classification) in 2019. We estimated country-level RSV-associated acute lower respiratory infection incidence through a risk-factor based model. We developed new models (through GLMM) that incorporated the latest RSV community mortality data for estimating overall RSV mortality. This review was registered in PROSPERO (CRD42021252400).<br>Findings In addition to 317 studies included in our previous review, we identified and included 113 new eligible studies and unpublished data from 51 studies, for a total of 481 studies. We estimated that globally in 2019, there were 33.0 million RSV-associated acute lower respiratory infection episodes (uncertainty range [UR] 25.4-44.6 million), 3.6 million RSV-associated acute lower respiratory infection hospital admissions (2.9-4.6 million), 26 300 RSV-associated acute lower respiratory infection in-hospital deaths (15100-49 100), and 101 400 RSV-attributable overall deaths (84 500-125 200) in children aged 0-60 months. In infants aged 0-6 months, we estimated that there were 6.6 million RSV-associated acute lower respiratory infection episodes (4.6-9.7 million), 1.4 million RSV-associated acute lower respiratory infection hospital admissions (1.0-2.0 million), 13 300 RSV-associated acute lower respiratory infection in-hospital deaths (6800-28 100), and 45 700 RSV-attributable overall deaths (38 400-55 900). 2.0% of deaths in children aged 0-60 months (UR 1.6-2.4) and 3.6% of deaths in children aged 28 days to 6 months (3.0-4.4) were attributable to RSV. More than 95% of RSV-associated acute lower respiratory infection episodes and more than 97% of RSV-attributable deaths across all age bands were in low-income and middle-income countries (LMICs).<br>Interpretation RSV contributes substantially to morbidity and mortality burden globally in children aged 0-60 months, especially during the first 6 months of life and in LMICs. We highlight the striking overall mortality burden of RSV disease worldwide, with one in every 50 deaths in children aged 0-60 months and one in every 28 deaths in children aged 28

		days to 6 months attributable to RSV. For every RSV-associated acute lower respiratory infection in-hospital death, we estimate approximately three more deaths attributable to RSV in the community. RSV passive immunisation programmes targeting protection during the first 6 months of life could have a substantial effect on reducing RSV disease burden, although more data are needed to understand the implications of the potential age-shifts in peak RSV burden to older age when these are implemented. Copyright © 2022 The Author(s). Published by Elsevier Ltd. This 24 san Open Access article under the CC BY 4.0 license.
36.	Hrabar J, Trumbić Ž, Bočina I, Bušelić I, Vrbatović A, Mladineo I. Interplay between proinflammatory cytokines, miRNA, and tissue lesions in Anisakis-infected Sprague-Dawley rats. PloS Negl Trop Dis. 2019 May 15;13(5):e0007397. doi: 10.1371/journal.pntd.0007397.	Background Anisakiasis 24 san emerging public health problem, caused by Anisakis spp. nematode larvae. Anisakiasis presents as variable and unspecific gastrointestinal and/or allergic clinical symptoms, which accounts for the high rate of misdiagnosed cases. Methodology/Principal findings The aim of this study was to characterize the early cellular (6-72 h p.i.) and molecular (6 h p.i.) immune response and general underlying regulatory mechanism in Anisakis infected rats. Each Sprague-Dawley rat was infected with 10 Anisakis spp. larvae by gastric intubation. Tissues with visible lesions were processed for: i) classic histopathology (HE), immunofluorescence (CD3, iNOS, S100A8/A9), and transmission electron microscopy (TEM); ii) target genes (Il1b, Il6, Il18, Ccl3, Icam1, Mmp9) and microRNA (Rat Immunopathology MIRN-104ZF plate, Quiagen) expression analysis; and iii) global DNA methylation. Histopathology revealed that Anisakis larval migration caused moderate to extensive hemorrhages in submucosal and epimysial/perimysial connective tissue. In stomach and muscle, moderate to abundant mixed inflammatory infiltrate was present, dominated by neutrophils and macrophages, while only mild infiltration was seen in intestine. Lesions were characterized by the presence of CD3(+), iNOS(+), and S100A8/A9(+) cells. The greatest number of iNOS(+) and S100A8/A9(+) cells was seen in muscle. Il6, Il1b, and Ccl3 showed particularly strong expression in stomach and visceral adipose tissues, but the order of expression differed between tissues. In total, three miRNAs were differentially expressed, two in stomach (miRNA-451 and miRNA-223) and two in intestine (miRNA-451 and miRNA-672). No changes in global DNA methylation were observed in infected tissues relative to controls.<br>Conclusions/Significance Anisakis infection induces strong immune responses in infected rats with marked induction of specific proinflammatory cytokines and miRNA expression. Deciphering the functional role of these cytokines and miRNAs will help in understanding the anisakiasis pathology and controversies surrounding Anisakis infection in humans. Author summary Anisakiasis is a zoonotic disease (infection transmitted between animals and humans) contracted by consumption of raw or undercooked seafood contaminated

		with Anisakis spp. nematode larvae. Anisakiasis usually presents with variable and unspecific gastrointestinal and/or allergic symptoms, which accounts for the high rate of misdiagnosed cases. Due to changes in dietary habits, such as eating raw or undercooked seafood, anisakiasis is considered an emerging public health problem. Despite the increase in number of reported cases worldwide, mechanisms of immune response to this unspecific human pathogen are poorly known. We have shown that in experimentally infected rats, Anisakis larvae cause severe hemorrhages and necrotic changes of affected tissues in the early phase of infections. Neutrophils and macrophages were abundantly present in tissue lesions, while eosinophils, hallmark of helminth infections, were scarcely present. We have also demonstrated particularly strong expression of several inflammatory genes. Moreover, we give for the first-time insight into putative regulatory mechanism mediated via a distinct class of RNA molecules. Our study may provide new opportunities for better understanding of cellular and molecular response to Anisakis spp., aiming at development of more specific therapeutics and alleviation of pathologies associated with Anisakis spp. infection.
37.	Dobec M, Golubic D, Punda-Polic V, Kaeppeli F, Sievers M. Rickettsia helvetica in Dermacentor reticulatus ticks. Emerg Infect Dis. 2009 Jan;15(1):98-100. doi: 10.3201/eid1501.080815.	We report on the molecular evidence that Dermacentor reticulatus ticks in Croatia are infected with Rickettsia helvetica (10%) or Rickettsia slovaca (2%) or co-infected with both species (1%). These findings expand the knowledge of the geographic distribution of R. helvetica and D. reticulatus ticks.
	**Medicine, General & Internal**	
38.	Punda-Polić V, Bradarić N, Grgić D. A 9-year-old with fever and severe muscle pains. Lancet. 1997 Jun 7;349(9066):1666. doi: 10.1016/S0140-6736(97)03316-3.	No abstract available, case report
39.	Chen Y, Yang K, Marušic A, Qaseem A, Meerpohl JJ, Flottorp S, Akl EA, Schünemann HJ, Chan ES, Falck-Ytter Y, Ahmed F, Barber S, Chen C, Zhang M, Xu B, Tian J, Song F, Shang H, Tang K, Wang Q, Norris SL; RIGHT (Reporting Items for Practice Guidelines in Healthcare) Working Group. A Reporting Tool for Practice Guidelines in Health Care: The RIGHT Statement. Ann Intern Med. 2017 Jan 17;166(2):128-132. doi: 10.7326/M16-1565.	The quality of reporting practice guidelines is often poor, and there is no widely accepted guidance or standards for such reporting in health care. The international RIGHT (Reporting Items for practice Guidelines in HealThcare) Working Group was established to address this gap. The group followed an existing framework for developing guidelines for health research reporting and the EQUATOR (Enhancing the QUAlity and Transparency Of health Research) Network approach. It developed a checklist and an explanation and elaboration statement. The RIGHT checklist includes 22 items that are considered essential for good reporting of practice guidelines: basic information (items 1 to 4), background (items 5 to 9), evidence (items 10 to 12), recommendations (items 13 to 15), review and quality assur-ance (items 16 and 17), funding and declaration and management of interests (items 18 and 19), and other information (items 20 to 22). The RIGHT checklist

		can assist developers in reporting guidelines, support journal editors and peer reviewers when considering guideline reports, and help health care practitioners understand and implement a guideline.
40.	Walley J, Lawn JE, Tinker A, de Francisco A, Chopra M, Rudan I, Bhutta ZA, Black RE; Lancet Alma-Ata Working Group. Primary health care: making Alma-Ata a reality. Lancet. 2008 Sep 13;372(9642):1001-7. doi: 10.1016/S0140-6736(08)61409-9.	The principles agreed at Alma-Ata 30 years ago apply just as much now as they did then. „Health for all" by the year 2000 was not achieved, and the Millennium Development Goals (MDGs) for 2015 will not be met in most low-income countries without substantial acceleration of primary health care. Factors have included insufficient political prioritisation of health, structural adjustment policies, poor governance, population growth, inadequate health systems, and scarce research and assessment on primary health care. We propose the following priorities for revitalising primary health care. Health-service infrastructure, including human resources and essential drugs, needs strengthening, and user fees should be removed for primary health-care services to improve use. A continuum of care for maternal, newborn, and child health services, including family planning, is needed. Evidence-based, integrated packages of community and primary curative and preventive care should be adapted to country contexts, assessed, and scaled up. Community participation and community health workers linked to strengthened primary-care facilities and first-referral services are needed. Furthermore, intersectoral action linking health and development is necessary, including that for better water, sanitation, nutrition, food security, and HIV control. Chronic diseases, mental health, and child development should be addressed. Progress should be measured and accountability assured. We prioritise research questions and suggest actions and measures for stakeholders both locally and globally, which are required to revitalise primary health care.
41.	Stevens GA, Alkema L, Black RE, Boerma JT, Collins GS, Ezzati M, Grove JT, Hogan DR, Hogan MC, Horton R, Lawn JE, Marušić A, Mathers CD, Murray CJ, Rudan I, Salomon JA, Simpson PJ, Vos T, Welch V; (The GATHER Working Group). Guidelines for Accurate and Transparent Health Estimates Reporting: the GATHER statement. Lancet. 2016 Dec 10;388(10062):e19-e23. doi: 10.1016/S0140-6736(16)30388-9	Measurements of health indicators are rarely available for every population and period of interest, and available data may not be comparable. The Guidelines for Accurate and Transparent Health Estimates Reporting (GATHER) defi ne best reporting practices for studies that calculate health estimates for multiple populations (in time or space) using multiple information sources. Health estimates that fall within the scope of GATHER include all quantitative population- level estimates (including global, regional, national, or subnational estimates) of health indicators, including indicators of health status, incidence and prevalence of diseases, injuries, and disability and functioning; and indicators of health determinants, including health behaviours and health exposures. GATHER comprises a checklist of 18 items that are essential for best reporting practice. A more detailed explanation and elaboration document, describing the interpretation and rationale of each reporting item along with examples of good reporting, is available on the GATHER website.

42.	Stevens GA, Alkema L, Black RE, Boerma JT, Collins GS, Ezzati M, Grove JT, Hogan DR, Hogan MC, Horton R, Lawn JE, Marušić A, Mathers CD, Murray CJ, Rudan I, Salomon JA, Simpson PJ, Vos T, Welch V; GATHER Working Group. Guidelines for Accurate and Transparent Health Estimates Reporting: the GATHER statement. PloS Med. 2016 Jun 28;13(6):e1002056. doi: 10.1371/journal.pmed.1002056. Erratum in: PloS Med. 2016 Aug 9;13(8):e1002116. doi: 10.1371/journal.pmed.1002116.	Measurements of health indicators are rarely available for every population and period of interest, and available data may not be comparable. The Guidelines for Accurate and Transparent Health Estimates Reporting (GATHER) define best reporting practices for studies that calculate health estimates for multiple populations (in time or space) using multiple information sources. Health estimates that fall within the scope of GATHER include all quantitative population-level estimates (including global, regional, national, or subnational estimates) of health indicators, including indicators of health status, incidence and prevalence of diseases, injuries, and disability and functioning; and indicators of health determinants, including health behaviours and health exposures. GATHER comprises a checklist of 18 items that are essential for best reporting practice. A more detailed explanation and elaboration document, describing the interpretation and rationale of each reporting item along with examples of good reporting, is available on the GATHER website (gather-statement.org).
43.	Tomlinson M, Swartz L, Officer A, Chan KY, Rudan I, Saxena S. Research priorities for health of people with disabilities: an expert opinion exercise. Lancet. 2009 Nov 28;374(9704):1857-62. doi: 10.1016/S0140-6736(09)61910-3.	International evidence shows that people with disabilities have many unmet health and rehabilitation needs, face barriers in accessing mainstream health-care services, and consequently have poor health. Inadequate specific information is available about the prevalence and patterns of health conditions of people with disabilities, effective interventions, and policy-relevant research about what works to improve health and functioning of people with disabilities. In view of the urgency of the issues at stake and scarcity of resources, research contributing to improvement of health of people with disabilities needs to be prioritised. We invited 82 stakeholders to list and score research options, with the priority-setting method of the Child Health and Nutrition Research Initiative. 83 research questions were assessed for answerability, applicability, sensitivity support within the context, and equity. The leading research priority was identification of barriers that people with disabilities have in accessing health services at different levels, and finding the best possible strategies to integrate their needs into primary health-care systems and ensure local delivery. Results showed that addressing specific impairments is secondary to ensuring that health systems provide adequately for all people with disabilities. Our findings are a call for urgent attention to the issue of access to appropriate health care for people with disabilities, especially in low-income and middle-income countries.
44.	Punda-Polić V, Sardelić S, Bradarić N. Visceral leishmaniasis in southern Croatia. Lancet. 1998 Jan 17;351(9097):188. doi: 10.1016/S0140-6736(05)78208-8.	No abstract available

	**Medicine, Research & Experiment**	
45.	Popovic D, Vucic D, Dikic I. Ubiquitination in disease pathogenesis and treatment. Nat Med. 2014 Nov;20(11):1242-53. doi: 10.1038/nm.3739.	Ubiquitination is crucial for a plethora of physiological processes, including cell survival and differentiation and innate and adaptive immunity. In recent years, considerable progress has been made in the understanding of the molecular action of ubiquitin in signaling pathways and how alterations in the ubiquitin system lead to the development of distinct human diseases. Here we describe the role of ubiquitination in the onset and progression of cancer, metabolic syndromes, neurodegenerative diseases, autoimmunity, inflammatory disorders, infection and muscle dystrophies. Moreover, we indicate how current knowledge could be exploited for the development of new clinical therapies.
	**Nursing**	
46.	Bošković L, Gašparić M, Petrić Miše B, Petković M, Gugić D, Ban M, Jazvić M, Dabelić N, Belac Lovasić I, Vrdoljak E. Optimisation of breast cancer patients' follow-up – potential way to improve cancer care in transitional countries. Eur J Cancer Care (Engl). 2017 Jul;26(4). doi: 10.1111/ecc.12514.	The aim of this analysis was to evaluate adherence of Croatian oncologists to follow-up criteria as suggested by the current national and international guidelines for women with breast cancer receiving adjuvant endocrine therapy. The use of clinical and diagnostic methods was documented in this prospective, non-interventional, multicenter study. A total of 438 post-menopausal patients receiving adjuvant endocrine treatment with non-steroidal aromatase inhibitors were included. Average annual frequency for each clinical and diagnostic method was calculated. Median adjuvant endocrine treatment duration before study recruitment was 10.5 months (interquartile 4.7-26.6). Patients were followed up for an average 23.5 +/- 4.9 months. Average number of oncological visits was 5.3. Mammograms were performed at mean annual frequency of 0.7, chest radiographs at 0.5, abdominal ultrasounds at 0.9, breast ultrasounds at 1.2, complete blood counts and chemistry panels at 1.7, carcinoembryonic antigen at 0.8, cancer antigen 15-3 at 1.6, gynaecological examination at 0.3, and densitometry at mean annual frequency of 0.3. In conclusion, among post-menopausal women with breast cancer receiving adjuvant endocrine therapy in this study, more unnecessary and unproven follow-up procedures were done compared to the guidelines' recommendations.
47.	Bokan I, Buljan I, Marušić M, Malički M, Marušić A. Predictors of academic success and aspirations in secondary nursing education: A cross-sectional study in Croatia. Nurse Educ Today. 2020 Feb 19;88:104370. doi: 10.1016/j.nedt.2020.104370.	Background: Academic success of students in nursing education 28 san issue causing concern in many parts of the world because of the shortage of nurses in the labour market, versatility of the educational programs and status of the students during studies and after graduation.<br>Objectives: To determine the predictors of academic success and educational aspirations of secondary nursing school students to enrol in higher education programs.<br>Participants and methods: This cross-sectional study included 312 students (92.6% response rate) from the Health School in Split, Croatia, enrolled in nursing program in 2015/2016. Besides sociodemographic data and grade point average (GPA), we collected

		data using 9 instruments: performance self-efficacy, academic self-efficacy, perseverance, attitudes towards science, self-respect, motivation, dispositional hope, future time perspective, and perceived personal incompetence.<br>Results: In a linear regression analysis, performance self-efficacy (beta = 0.38) and future time orientation (beta = 0.19) proved to be significant predictors of current GPA, explaining 20% of the variance. In logistic regression, participants' aspiration to enrol in higher education rather than get employed after secondary school graduation was predicted by higher extrinsic motivation (odds ratio (OR) = 1.11, 95% confidence interval (CI) = 1.06 to 1.17) and higher performance self-efficacy (OR = 3.06, 95% CI = 1.62 to 5.77); explaining approximately 26% of the variance in the results.<br>Conclusion: Performance self-efficacy and future time orientation were the main predictors of academic success of Croatian nursing students at the secondary education level. Higher performance self-efficacy and extrinsic motivation were associated with aspirations to continue university education.
48.	Knezevic B, Milosevic M, Golubic R, Belosevic L, Russo A, Mustajbegovic J. Work-related stress and work ability among Croatian university hospital midwives. Midwifery. 2011 Apr;27(2):146-53. doi: 10.1016/j.midw.2009.04.002.	Objective: to explore the sources and levels of stress at work and work ability among Croatian midwives.<br>Background: midwives are subjected to multiple stressors. Among health-care professionals, psychological distress for a prolonged period of time has negative effects on the worker's health, work ability and quality of patient care. 'Work ability' is a term describing a worker's resources related to physical, mental and social demands at work. As a measure of work ability in midwifery, the Work Ability © (WAI) is considered to be a very predictive instrument; midwives with a poor WAI score usually leave their current job within five years.<br>Setting: university hospitals in Zagreb, Croatia.<br>Design: cross-sectional design survey.<br>Participants: 300 health-care workers (105 qualified midwives and 195 paediatric nurses) were invited to complete the questionnaire. The total response rate was 53% (1581300). The sample included 14.7% of all hospital-based midwives in Zagreb hospitals.<br>Methods: the Occupational Stress Assessment Questionnaire (OSAQ) for health-care workers and the WAI questionnaire.<br>Findings: over three-quarters of the midwives (46160, 76.7%) believed that their job was stressful, and considered that insufficient work resources caused the most stress. More than half of the midwives associated an insufficient number of coworkers, unexpected situations, inadequate income, night work, incurable patients and poor organisation at work with a high level of stress. The perceived specific stressors differed between

		midwives and paediatric nurses in the same hospital. Insufficient work resources and poor organisation at work were more common stressors among midwives than paediatric nurses ($p<0.05$). Midwives and nurses differed significantly with respect to age ($p = 0.002$). Midwives were younger and had spent fewer years working in their current workplace compared with paediatric nurses ($p<0.001$). Also, midwives had a lower level of education than paediatric nurses ($p = 0.044$). The mean WAI score for midwives was 40.0 [95% confidence interval (CI) 38.4-41.41, compared with 37.5 (95% CI 36.4-38.8) for paediatric nurses, both indicating good work ability. After adjusting for age, the difference in WAI score between the groups of workers was not significant.<br>Conclusions: Croatian midwives experienced work-related stress due to: insufficient work resourses, insufficient number of coworkers, poor organisation at work, communication with superiors and emotional work. Midwives' work ability in relation to the demands of their job was good. These results confirmed that the WAI score decreases significantly with age.<br>Implications for practice: hospital management needs to improve organisational factors and resources, as well as midwives' education and position in the health-care system. © 2009 Elsevier Ltd. All rights reserved.
	**Obstetrics & Gynecology**	
49.	Sunj M, Canic T, Baldani DP, Tandara M, Jeroncic A, Palada I. Does unilateral laparoscopic diathermy adjusted to ovarian volume increase the chances of ovulation in women with polycystic ovary syndrome? Hum Reprod. 2013 Sep;28(9):2417-24. doi: 10.1093/humrep/det273.	Does unilateral volume-adjusted laparoscopic diathermy increase the chances of ovulation in women with polycystic ovary syndrome (PCOS)?<br>Although unilateral laparoscopic ovarian drilling (ULOD) using adjusted thermal doses was more efficient than bilateral laparoscopic ovarian drilling (BLOD) using fixed doses, the chances of ovulation were improved in patients irrespective of the technique used.<br>The adjustment of the thermal dose to ovarian volume in BLOD increases ovulation and pregnancy rates compared with fixed-dose treatment, but BLOD causes the formation of adhesions, particularly on the left ovary, and increases the risk of damage to ovarian tissue. In contrast, ULOD with a fixed thermal dose minimizes the risk of ovarian tissue damage, and can increase the activity in both right and left ovaries, although this varies in humans and in other species.<br>This prospective, longitudinal, study, between September 2009 and January 2013, included 96 infertile women with PCOS who were unresponsive to clomiphene citrate treatment and had underwent either ULOD or BLOD. After surgery, the groups were followed up for 6 months to assess ovulatory response.<br>Patients were assigned to two groups; one group underwent laparoscopic ovarian drilling of the right ovary alone, while both ovaries were treated in the second group. The ULOD

		group (n 49) received thermal doses adjusted to the volume of the right ovary (60 J/cm(3)). The BLOD group (n 47) received fixed doses of 600 J per ovary, regardless of its volume. The two treatment groups were matched by the number of participants, age and baseline parameters.<br>The ovulation rate during the first menstrual cycle after LOD was significantly higher in the ULOD group than in the BLOD group [73 versus 49; absolute risk reduction (ARR), 0.25; 95 confidence interval (CI), 0.44 to 0.03; P 0.014]. Treatment with ULOD on the right ovary significantly increased the chances of ovulation in patients with a larger right ovary compared with those who had a smaller right ovary (100 versus 36; ARR, 0.64; 95 CI, 0.84 to 0.37; P 0.004). Interestingly, the chances of ovulation were also significantly higher in patients in the BLOD group who had a larger right ovary compared with those who had a smaller right ovary (88 versus 33; ARR, 0.55; 95 CI, 0.73 to 0.28; P 0.002). The pregnancy rate was also significantly higher in patients with a larger right ovary compared with those with a smaller right ovary, regardless of the treatment group.<br>The 6-month follow-up was too short to demonstrate any long-term differences in the ovulation rates. Future research should therefore extend the follow-up beyond 6 months. Another limitation is that ULOD was used to treat only the right ovary. Future studies should investigate whether ULOD treatment of the larger ovary, whether left or right, would significantly increase the ovulation rate.<br>This study represents an advance in the determination of the optimal laparoscopic treatment for women with PCOS, as it was shown that improved results can be achieved using less thermal energy in volume-adjusted ULOD.
50.	Strinić T, Eterović D. Oral contraceptives improve lung mechanics. Fertil Steril. 2003 May;79(5):1070-3. doi: 10.1016/s0015-0282(02)04961-0.	Objective: To determine whether oral contraceptives affect lung mechanics.<br>Design: Open-label study. Setting: Academic medical center.<br>Patient(s): Thirty-six healthy nonsmoking women.<br>Intervention(s): Administration of an oral contraceptive containing ethinyl estradiol, 35 mug, and norgestimat, 250 mug for 6 months.<br>Main Outcome Measure(s): Forced vital capacity; forced expiratory volume in I second; peak expiratory flow; and flow at large, medium, and small lung volumes.<br>Result(s): At 6 months, all forced expiratory flow and volume had increased significantly (from 6.5% to 15%). Flows at small lung volumes especially increased.<br>Conclusion(s): Combination oral contraceptives have a measurable effect on lung mechanics. © 2003 by American Society for Reproductive Medicine.
	**Oncology**	

51.	von Minckwitz G, Puglisi F, Cortes J, Vrdoljak E, Marschner N, Zielinski C, Villanueva C, Romieu G, Lang I, Ciruelos E, De Laurentiis M, Veyret C, de Ducla S, Freudensprung U, Srock S, Gligorov J. Bevacizumab plus chemotherapy versus chemotherapy alone as second-line treatment for patients with HER2-negative locally recurrent or metastatic breast cancer after first-line treatment with bevacizumab plus chemotherapy (TANIA): an open-label, randomised phase 3 trial. Lancet Oncol. 2014 Oct;15(11):1269-78. doi: 10.1016/S1470-2045(14)70439-5.	Background Combining bevacizumab with first-line or second-line chemotherapy improves progression-free survival in HER2-negative locally recurrent or metastatic breast cancer. We assessed the efficacy and safety of further bevacizumab therapy in patients with locally recurrent or metastatic breast cancer whose disease had progressed after treatment with bevacizumab plus chemotherapy.<br>Methods In this open-label, randomised, phase 3 trial, we recruited patients who had HER2-negative locally recurrent or metastatic breast cancer that had progressed after receiving 12 weeks or more of first-line bevacizumab plus chemotherapy from 118 centres in 12 countries. Patients were randomly assigned (1:1) by use of a central interactive voice response system using a block randomisation schedule (block size four) stratified by hormone receptor status, first-line progression-free survival, selected chemotherapy, and lactate dehydrogenase concentration, to receive second-line single-agent chemotherapy either alone or with bevacizumab (15 mg/kg every 3 weeks or 10 mg/kg every 2 weeks). Second-line therapy was continued until disease progression, unacceptable toxicity, or consent withdrawal. At progression, patients randomly assigned to chemotherapy alone received third-line chemotherapy without bevacizumab; those randomly assigned to bevacizumab continued bevacizumab with third-line chemotherapy. The primary endpoint was progression-free survival from randomisation to second-line progression or death in the intention-to-treat population. This trial is ongoing, and registered with ClinicalTrials.gov, number NCT01250379.<br>Findings Between Feb 17, 2011, and April 3, 2013, 494 patients were randomly assigned to treatment (247 in each group). The median duration of follow-up at the time of this prespecified primary progression-free survival analysis was 15.9 months (IQR 9.1-21.7) in the chemotherapy-alone group and 16.1 months (10.6-22.7) in the combination group. Progression-free survival was significantly longer for those patients treated with bevacizumab plus chemotherapy than for those with chemotherapy alone (median: 6.3 months [95% CI 5.4-7.2] vs 4.2 months [3.9-4.7], respectively, stratified hazard ratio [HR] 0.75 [95% CI 0.61-0.93], two-sided stratified log-rank p=0.0068). The most common grade 3 or more adverse events were hypertension (33 [13%] of 245 patients receiving bevacizumab plus chemotherapy vs 17 [7%] of 238 patients receiving chemotherapy alone), neutropenia (29 [12%] vs 20 [8%]), and hand-foot syndrome (27 [11%] vs 25 [11%]). Grade 3 proteinuria occurred in 17 (7%) of 245 patients receiving combination therapy and one (<1%) of 238 patients receiving chemotherapy alone. Serious adverse events were reported in 61 (25%) of 245 patients receiving bevacizumab plus chemotherapy versus 44 (18%) of 238 patients receiving chemotherapy alone.

		Interpretation These results suggest that continued VEGF inhibition with further bevacizumab is a valid treatment option for patients with locally recurrent or metastatic HER2-negative breast cancer whose disease was stabilised or responded to first-line bevacizumab with chemotherapy.
52.	Vrdoljak E, Marschner N, Zielinski C, Gligorov J, Cortes J, Puglisi F, Aapro M, Fallowfield L, Fontana A, Inbar M, Kahan Z, Welt A, Lévy C, Brain E, Pivot X, Putzu C, González Martín A, de Ducla S, Easton V, von Minckwitz G. Final results of the TANIA randomised phase III trial of bevacizumab after progression on first-line bevacizumab therapy for HER2-negative locally recurrent/metastatic breast cancer. Ann Oncol. 2016 Nov;27(11):2046-2052. doi: 10.1093/annonc/mdw316.	The randomised phase III TANIA trial demonstrated that continuing bevacizumab with second-line chemotherapy for locally recurrent/metastatic breast cancer (LR/mBC) after progression on first-line bevacizumab-containing therapy significantly improved progression-free survival (PFS) compared with chemotherapy alone [hazard ratio (HR) = 0.75, 95% confidence interval (CI) 0.61-0.93]. We report final results from the TANIA trial, including overall survival (OS) and health-related quality of life (HRQoL).<br>Patients with HER2-negative LR/mBC that had progressed on 33 san33er first-line bevacizumab plus chemotherapy were randomised to receive standard second-line chemotherapy either alone or with bevacizumab. At second progression, patients initially randomised to bevacizumab continued bevacizumab with their third-line chemotherapy, but those randomised to chemotherapy alone were not allowed to cross over to receive third-line bevacizumab. The primary end point was second-line PFS; secondary end points included third-line PFS, combined second- and third-line PFS, OS, HRQoL and safety.<br>Of the 494 patients randomised, 483 received second-line therapy; 234 patients (47% of the randomised population) continued to third-line study treatment. The median duration of follow-up at the final analysis was 32.1 months in the chemotherapy-alone arm and 30.9 months in the bevacizumab plus chemotherapy arm. There was no statistically significant difference between treatment arms in third-line PFS (HR = 0.79, 95% CI 0.59-1.06), combined second- and third-line PFS (HR = 0.85, 95% CI 0.68-1.05) or OS (HR = 0.96, 95% CI 0.76-1.21). Third-line safety results showed increased incidences of proteinuria and hypertension with bevacizumab, consistent with safety results for the second-line treatment phase. No differences in HRQoL were detected.<br>In this trial, continuing bevacizumab beyond first and second progression of LR/mBC improved second-line PFS, but no improvement in longer term efficacy was observed. The second-line PFS benefit appears to be achieved without detrimentally affecting quality of life.
53.	Grivennikov S, Karin E, Terzic J, Mucida D, Yu GY, Vallabhapurapu S, Scheller J, Rose-John S, Cheroutre H, Eckmann L, Karin M. IL-6 and Stat3 are required for survival of intestinal epithelial cells and development of colitis-associated cancer. Cancer	Colitis-associated cancer (CAC) is the most serious complication of inflammatory bowel disease. Proinflammatory cytokines have been suggested to regulate preneoplastic growth during CAC tumorigenesis. Interleukin 6 (IL-6) is a multifunctional NF-kappa B-regulated cytokine that acts on epithelial and immune cells. Using genetic tools, we now demonstrate that IL-6 is a critical tumor promoter during early CAC tumorigenesis. In

	Cell. 2009 Feb 3;15(2):103-13. doi: 10.1016/j.ccr.2009.01.001. Erratum in: Cancer Cell. 2009 Mar 3;15(3):241.	addition to enhancing proliferation of tumor-initiating cells, IL-6 produced by lamina propria myeloid cells protects normal and premalignant intestinal epithelial cells (IECs) from apoptosis. The proliferative and survival effects of IL-6 are largely mediated by the transcription factor Stat3, whose IEC-specific ablation has profound impact on CAC tumorigenesis. Thus, the NF-kappa B-IL-6-Stat3 cascade 34 san important regulator of the proliferation and survival of tumor-initiating IECs.
54.	Sparano JA, Vrdoljak E, Rixe O, Xu B, Manikhas A, Medina C, Da Costa SC, Ro J, Rubio G, Rondinon M, Perez Manga G, Peck R, Poulart V, Conte P. Randomized phase III trial of ixabepilone plus capecitabine versus capecitabine in patients with metastatic breast cancer previously treated with an anthracycline and a taxane. J Clin Oncol. 2010 Jul 10;28(20):3256-63. doi: 10.1200/JCO.2009.24.4244.	Purpose We sought to determine whether the combination of ixabepilone plus capecitabine improved overall survival (OS) compared with capecitabine alone in patients with metastatic breast cancer (MBC) previously treated with anthracyclines and taxanes.<br>Patients and Methods A total of 1,221 patients with MBC previously treated with anthracycline and taxanes were randomly assigned to ixabepilone (40 mg/m(2) intravenously on day 1) plus capecitabine (2,000 mg/m(2) orally on days 1 through 14) or capecitabine alone (2,500 mg/m(2) on the same schedule) given every 21 days. The trial was powered to detect a 20% reduction in the hazard ratio (HR) for death.<br>Results There was no significant difference in OS between the combination and capecitabine monotherapy arm, the primary end point (median, 16.4 v 15.6 months; HR = 0.9; 95% CI, 078 to 1.03; P = .1162). The arms were well balanced with the exception of a higher prevalence of impaired performance status (Karnofsky performance status 70% to 80%) in the combination arm (32% v 25%). In a secondary Cox regression analysis adjusted for performance status and other prognostic factors, OS was improved for the combination (HR = 0.85; 95% CI, 0.75 to 0.98; P = .0231). In 79% of patients with measurable disease, the combination significantly improved progression-free survival (PFS; median, 6.2 v 4.2 months; HR = 0.79; P = .0005) and response rate (43% v 29%; P < .0001). Grade 3 to 4 neuropathy occurred in 24% treated with the combination, but was reversible.<br>Conclusion This study confirmed a previous trial demonstrating improved PFS and response for the ixabepilone-capecitabine combination compared with capecitabine alone, although this did not result in improved survival.
55.	Gore ME, Szczylik C, Porta C, Bracarda S, Bjarnason GA, Oudard S, Hariharan S, Lee SH, Haanen J, Castellano D, Vrdoljak E, Schöffski P, Mainwaring P, Nieto A, Yuan J, Bukowski R. Safety and efficacy of sunitinib for metastatic renal-cell carcinoma: an expanded-access trial. Lancet Oncol. 2009 Aug;10(8):757-63. doi: 10.1016/S1470-2045(09)70162-7.	Background Results from clinical trials have established sunitinib as a standard of care for first-line treatment of advanced or metastatic renal-cell carcinoma (RCC); however, many patients, particularly those with a poorer prognosis, do not meet inclusion criteria and little is known about the activity of sunitinib in these subgroups. The primary objective of this trial was to provide sunitinib on a compassionate-use basis to trial-ineligible patients with RCC from countries where regulatory approval had not been granted.<br>Methods Previously treated and treatment-naive patients at least 18 years of age with metastatic RCC were eligible. All patients received open-label sunitinib 50 mg orally once

		daily on schedule 4-2 (4 weeks on treatment, 2 weeks off). Safety was assessed regularly, tumour measurements done per local practice, and survival data collected where possible. Analyses were done in the modified intention-to-treat (ITT) population, which consisted of all patients who received at least one dose of sunitinib. This study is registered with ClinicalTrials.gov, NCT00130897.<br>Findings As of December, 2007, 4564 patients were enrolled in 52 countries. 4371 patients were included in the modified ITT population. This population included 321 (7%) patients with brain metastases, 582 (13%) with Eastern Cooperative Oncology Group (ECOG) performance status of 2 or higher, 588 (13%) non-clear-cell RCC, and 1418 (32%) aged 65 years or more. Patients received a median of five treatment cycles (range 1-25). Reasons for discontinuation included lack of efficacy (n=1168 [27%]) and adverse events (n=362 [8%]). The most common treatment-related adverse events were diarrhoea (n=1936 [44%]) and fatigue (n=1606 [37%]). The most common grade 3-4 adverse events were fatigue (n=344 [8%]) and thrombocytopenia (n=338 [8%]) with incidences of grade 3-4 adverse events similar across subgroups. In 3464 evaluable patients, the objective response rate (ORR) was 17% (n=603), with subgroup ORR as follows: brain metastases (26 of 213 [12%]), ECOG performance status 2 or higher (29 of 319 [9%]), non-clear-cell RCC (48 of 437 [11%]) and age 65 years or more (176 of 1056 [17%]). Median progression-free survival was 10-9 months (95% CI 10.3-11.2) and overall survival was 18.4 months (17.4-19-2).<br>Interpretation In a broad population of patients with metastatic RCC, the safety profile of sunitinib 50 mg once-daily (initial dose) on schedule 4-2 was manageable and efficacy results were encouraging, particularly in subgroups associated with poor prognosis who are not usually entered into clinical trials.<br>Funding Pfizer Inc.
56.	Coleman RE, Banks LM, Girgis SI, Kilburn LS, Vrdoljak E, Fox J, Cawthorn SJ, Patel A, Snowdon CF, Hall E, Bliss JM, Coombes RC; Intergroup Exemestane Study group. Skeletal effects of exemestane on bone-mineral density, bone biomarkers, and fracture incidence in postmenopausal women with early breast cancer participating in the Intergroup Exemestane Study (IES): a randomised controlled study. Lancet Oncol. 2007 Feb;8(2):119-27. doi: 10.1016/S1470-2045(07)70003-7.	Background Tamoxifen preserves bone in postmenopausal women, but non-steroidal aromatase inhibitors accelerate bone loss and increase fracture risk. We aimed to study the effect on bone health in a subgroup of women included in the Intergroup Exemestane Study (IES), a large randomised trial that compared the switch to the steroidal aromatase inhibitor exemestane with continuation of tamoxifen in the adjuvant treatment of postmenopausal breast cancer.<br>Methods Results were analysed from 206 evaluable patients from the IES, in which postmenopausal women with histologically confirmed and completely resected unilateral breast cancer (that was oestrogen-receptor positive or of unknown status), who were disease-free after 2-3 years of treatment with tamoxifen were randomised to continue oral

		tamoxifen 20 mg/day or switch to oral exemestane 25 mg/day to complete a total of 5 years of adjuvant endocrine therapy. The primary endpoint was change in bone-mineral density (BMD) assessed by dual energy X-ray absorptiometry. Changes in biochemical markers of bone turnover were also analysed in this substudy, and the incidence of fractures in the entire study reported. The IES is registered on the Current Controlled Trials website http://www.controlled-trials.com/ISRCTN11883920.<br>Findings Within 6 months of switching to exemestane, BMD was lowered by 0.051 g/cm(3) (2.7%; 95% CI 2.0-3.4; p < 0.0001) at the lumbar spine and 0.025 g/cm(3) (1.4%; 0.8-1.9; p < 0.0001) at the hip compared with baseline. BMD decreases were only 1.0% (0.4-1.7; p=0.002) and 0.8% (0.3-1.4; p=0.003) in year 2 at the lumbar spine and hip, respectively. No patient with BMD in the normal range at trial entry developed osteoporosis. Bone resorption and formation markers increased at all time points in women receiving exemestane (p < 0.001). With a median follow-up in all IES participants (n=4274) of 58 months, 162 (7%) and 115 (5%) patients in the exemestane and tamoxifen groups, respectively, had fractures (odds ratio 1.45 [1.13-1.87]; p=0.003).<br>Interpretation These results indicate that the increase in survival shown previously with the IES switch strategy is achieved at the expense of some detriment to skeletal health, so the risk-benefit ratio to women needs to be individually assessed.
57.	Gianni L, Dafni U, Gelber RD, Azambuja E, Muehlbauer S, Goldhirsch A, Untch M, Smith I, Baselga J, Jackisch C, Cameron D, Mano M, Pedrini JL, Veronesi A, Mendiola C, Pluzanska A, Semiglazov V, Vrdoljak E, Eckart MJ, Shen Z, Skiadopoulos G, Procter M, Pritchard KI, Piccart-Gebhart MJ, Bell R; Herceptin Adjuvant (HERA) Trial Study Team. Treatment with trastuzumab for 1 year after adjuvant chemotherapy in patients with HER2-positive early breast cancer: a 4-year follow-up of a randomised controlled trial. Lancet Oncol. 2011 Mar;12(3):236-44. doi: 10.1016/S1470-2045(11)70033-X.	Background Treatment with adjuvant trastuzumab for 1 year improves disease-free survival and overall survival in patients with human epidermal growth factor receptor 2 (HER2)-positive early breast cancer. We aimed to assess disease-free survival and overall survival after a median follow-up of 4 years for patients enrolled on the Herceptin Adjuvant (HERA) trial.<br>Methods The HERA trial 36 san international, multicentre, randomised, open-label, phase 3 trial comparing treatment with trastuzumab for 1 and 2 years with observation after standard neoadjuvant, adjuvant chemotherapy, or both in patients with HER2-positive early breast cancer. The primary endpoint was disease-free survival. After a positive first interim analysis at a median follow-up of 1 year for the comparison of treatment with trastuzumab for 1 year with observation, event-free patients in the observation group were allowed to cross over to receive trastuzumab. We report trial outcomes for the 1-year trastuzumab and observation groups at a median follow-up of 48.4 months (IQR 42.0-56.5) and assess the effect of the extensive crossover to trastuzumab. Our analysis was by intention-to-treat. The HERA trial is registered with the European Clinical Trials Database, number 2005-002385-11.<br>Findings The HERA trial population comprised 1698 patients randomly assigned to the

		observation group and 1703 to the 1-year trastuzumab group. Intention-to-treat analysis of disease-free survival showed a significant benefit in favour of patients in the 1-year trastuzumab group (4-year disease-free survival 78.6%) compared with the observation group (4-year disease-free survival 72.2%; hazard ratio [HR] 0.76; 95% CI 0.66-0.87; p<0.0001). Intention-to-treat analysis of overall survival showed no significant difference in the risk of death (4-year overall survival 89.3% vs 87.7%, respectively; HR 0.85; 95% CI 0.70-1.04; p=0.11). Overall, 885 patients (52%) of the 1698 patients in the observation group crossed over to receive trastuzumab, and began treatment at median 22.8 months (range 4.5-52.7) from randomisation. In a non-randomised comparison, patients in the selective-crossover cohort had fewer disease-free survival events than patients remaining in the observation group (adjusted HR 0.68; 95% CI 0.51-0.90; p=0.0077). Higher incidences of grade 3-4 and fatal adverse events were noted on 1-year trastuzumab than in the observation group. The most common grade 3 or 4 adverse events, each in less than 1% of patients, were congestive cardiac failure, hypertension, arthralgia, back pain, central-line infection, hot flush, headache, and diarrhoea.<br>Interpretation Treatment with adjuvant trastuzumab for 1 year after chemotherapy is associated with significant clinical benefit at 4-year median follow-up. The substantial selective crossover of patients in the observation group to trastuzumab was associated with improved outcomes for this cohort.
	**Ophthalmology**	
58.	Miyake M, Yamashiro K, Tabara Y, Suda K, Morooka S, Nakanishi H, Khor CC, Chen P, Qiao F, Nakata I, Akagi-Kurashige Y, Gotoh N, Tsujikawa A, Meguro A, Kusuhara S, Polasek O, Hayward C, Wright AF, Campbell H, Richardson AJ, Schache M, Takeuchi M, Mackey DA, Hewitt AW, Cuellar G, Shi Y, Huang L, Yang Z, Leung KH, Kao PYP, Yap MKH, Yip SP, Moriyama M, Ohno-Matsui K, Mizuki N, MacGregor S, Vitart V, Aung T, Saw SM, Tai ES, Wong TY, Cheng CY, Baird PN, Yamada R, Matsuda F; Nagahama Study Group; Yoshimura N. Identification of myopia-associated WNT7B polymorphisms provides insights into the mechanism underlying the development of myopia. Nat Commun. 2015 Mar 31;6:6689. doi: 10.1038/ncomms7689.	Myopia can cause severe visual impairment. Here, we report a two-stage genome-wide association study for three myopia-related traits in 9,804 Japanese individuals, which was extended with trans-ethnic replication in 2,674 Chinese and 2,690 Caucasian individuals. We identify WNT7B as a novel susceptibility gene for axial length (rs10453441, P-meta = 3.9 x 10(-13)) and corneal curvature (P-meta = 2.9 x 10(-40)) and confirm the previously reported association between GJD2 and myopia. WNT7B significantly associates with extreme myopia in a case-control study with 1,478 Asian patients and 4,689 controls (odds ratio (OR)(meta) = 1.13, P-meta = 0.011). We also find in a mouse model of myopia downregulation of WNT7B expression in the cornea and upregulation in the retina, suggesting its possible role in the development of myopia.

	**Orthopedics**	
59.	Smoljanovic T, Bojanic I, Hannafin JA, Hren D, Delimar D, Pecina M. Traumatic and overuse injuries among international elite junior rowers. Am J Sports Med. 2009 Jun;37(6):1193-9. doi: 10.1177/0363546508331205.	Background: Junior rowers have competed internationally for over 4 decades, and there are no epidemiological data available on traumatic and overuse injury in this population.<br>Objective: To define the types of musculoskeletal problems present in international elite-level junior rowers and to determine whether gender, physical stature, rowing discipline, and training programs affect the incidence of reported injuries.<br>Study Design: Descriptive epidemiology study.<br>Methods: Injury data were obtained from a total of 398 rowers (42% female, 58% male) who completed a 4-page questionnaire on injury incidence while participating at the Junior World Rowing Championships in Beijing, People's Republic of China, in August 2007.<br>Results: Overall, 290 (73.8%) reported injuries involved overuse, and 103 (26.2%) were related to a single traumatic event. Female rowers were injured more frequently than male rowers (110.2 vs 90.5 injuries per 100 rowers). In both genders, the most common injury site was the low back followed by the knee and the forearm/wrist. The severity of reported injuries was incidental in 65.1%, minor in 21.4%, moderate in 10.4%, and major in 3.1% of cases. The rowers with traumatic injuries had less rowing experience than the uninjured rowers (median [C] +/- interquartile range [Q] = 3 +/- 3 years vs 4 +/- 3 years; P = .043, Mann-Whitney test). Sweep rowers who changed rowing side during the current season had significantly more acute-onset low back injuries (P = .012, chi(2) test) than those who did not change rowing side during the same period. The incidence of traumatic injuries was significantly lower in rowers who regularly performed more than 10 minutes of posttraining stretching (P = .030, chi(2) test). Athletes who ran more than once a week had more overuse knee injuries than those who ran once or less per week (P = .033, chi(2) test).<br>Conclusion: Elite junior rowers attending the World Rowing Championships reported predominantly overuse injuries of low severity during the current rowing season. Low back injuries were the most frequent complaint of elite-level junior rowers.
	**Othorhinolaryngology**	
60.	Rudic M, Keogh I, Wagner R, Wilkinson E, Kiros N, Ferrary E, Sterkers O, Bozorg Grayeli A, Zarkovic K, Zarkovic N. The pathophysiology of otosclerosis: Review of current research. Hear Res. 2015 Dec;330(Pt A):51-6. doi: 10.1016/j.heares.2015.07.014.	Otosclerosis is a complex disease of the human otic capsule with highest incidence in adult Caucasians. So far, many possible etiological factors like genetics, HLA, autoimmunity, viruses, inflammation, and hormones have been investigated but still the development of the disease remains unclear. Currently, the surgical replacement of stapes (stapedotomy) remains the best possible treatment option. In this review, we analyze different etiological factors studied so far in otosclerosis pathophysiology and discuss most recent findings and

		possible new research pathways. This article is part of a Special Issue entitled . INDEKS 2015 Elsevier B.V. All rights reserved.
	**Pathology**	
61.	Starczynski J, Simmons W, Flavell JR, Byrd PJ, Stewart GS, Kullar HS, Groom A, Crocker J, Moss PA, Reynolds GM, Glavina-Durdov M, Taylor AM, Fegan C, Stankovic T, Murray PG. Variations in ATM protein expression during normal lymphoid differentiation and among B-cell-derived neoplasias. Am J Pathol. 2003 Aug;163(2):423-32. doi: 10.1016/S0002-9440(10)63672-3.	The ataxia telangiectasia mutated (ATM) protein plays a central role in the cellular response to DNA double-strand breaks (DSBs). Developmentally programmed. DSBs are restricted to cellular subsets within lymphoid tissues and we asked whether ATM expression is differentially regulated during lymphoid differentiation. We showed that immature B cells in bone marrow and immature T cells of the thymic cortex were negative or weakly ATM-positive. T cells of thymic medulla and peripheral tissues strongly expressed ATM. High levels of ATM were present in the B lymphocytes of the mantle zone and in plasma cells, while the majority of germinal center B cells were negative or weakly labeled. Therefore, ATM expression appears to be down-regulated at those stages of lymphoid development where physiological DNA DSBs occur. In B-chronic lymphocytic leukemia and mantle cell lymphoma we observed two categories: ATM-negative tumors, most likely reflecting the presence of ATM mutation, and tumors with abundant ATM expression. Most follicular center-cell lymphomas and diffuse large B-cell lymphomas, which rarely show inactivation of the ATM gene, were negative or weakly ATM-positive. Tumor cells from most cases of Hodgkin's disease were ATM-negative. Therefore, unless ATM inactivation occurs, ATM expression in lymphoid tumors is likely to reflect their cellular origin. As a result, immunostaining to identify lymphoid neoplasias with ATM inactivation might only be feasible for tumors derived from the stages where ATM is constitutively highly expressed.
	**Pediatrics**	
62.	Nair H, Nokes DJ, Gessner BD, Dherani M, Madhi SA, Singleton RJ, O'Brien KL, Roca A, Wright PF, Bruce N, Chandran A, Theodoratou E, Sutanto A, Sedyaningsih ER, Ngama M, Munywoki PK, Kartasasmita C, Simões EA, Rudan I, Weber MW, Campbell H. Global burden of acute lower respiratory infections due to respiratory syncytial virus in young children: a systematic review and meta-analysis. Lancet. 2010 May 1;375(9725):1545-55. doi: 10.1016/S0140-6736(10)60206-1.	Background The global burden of disease attributable to respiratory syncytial virus (RSV) remains unknown. We aimed to estimate the global incidence of and mortality from episodes of acute lower respiratory infection (ALRI) due to RSV in children younger than 5 years in 2005.<br>Methods We estimated the incidence of RSV-associated ALRI in children younger than 5 years, stratified by age, using data from a systematic review of studies published between January, 1995, and June, 2009, and ten unpublished population-based studies. We estimated possible boundaries for RSV-associated ALRI mortality by combining case fatality ratios with incidence estimates from hospital-based reports from published and unpublished studies and identifying studies with population-based data for RSV seasonality and monthly ALRI mortality.<br>Findings In 2005, an estimated 33.8 (95% CI 19.3-46-2) million new episodes of RSV-

		associated ALRI occurred worldwide in children younger than 5 years (22% of ALRI episodes), with at least 3.4 (2.8-4.3) million episodes representing severe RSV-associated ALRI necessitating hospital admission. We estimated that 66 000-199 000 children younger than 5 years died from RSV-associated ALRI in 2005, with 99% of these deaths occurring in developing countries. Incidence and mortality can vary substantially from year to year in any one setting.<br>Interpretation Globally, RSV is the most common cause of childhood ALRI and a major cause of admission to hospital as a result of severe ALRI. Mortality data suggest that RSV 40 san important cause of death in childhood from ALRI, after pneumococcal pneumonia and Haemophilus influenzae type b. The development of novel prevention and treatment strategies should be accelerated as a priority.
63.	Stupar D, Stevanovic D, Vostanis P, Atilola O, Moreira P, Dodig-Curkovic K, Franic T, Doric A, Davidovic N, Avicenna M, Multazam IN, Nussbaum L, Thabet AA, Ubalde D, Petrov P, Deljkovic A, Monteiro AL, Ribas A, Jovanovic M, Joana O, Knez R. Posttraumatic stress disorder symptoms among trauma-exposed adolescents from low- and middle-income countries. Child Adolesc Psychiatry Ment Health. 2021 Jun 5;15(1):26. doi: 10.1186/s13034-021-00378-2.	Background Exposure to traumatic events in childhood is associated with the development and maintenance of various psychiatric disorders, but most frequently with posttraumatic stress disorder (PTSD). The aim of this study was to evaluate the types of traumatic events experienced and the presence and predictors of PTSD symptoms among adolescents from the general population from ten low- and middle-income countries (LMICs). Methods Data were simultaneously collected from 3370 trauma-exposed adolescents (mean age = 15.41 [SD = 1.65] years, range 12-18; 1465 (43.5%) males and 1905 (56.5%) females) in Brazil, Bulgaria, Croatia, Indonesia, Montenegro, Nigeria, the Palestinian Territories, the Philippines, Romania, and Serbia, with Portugal, a high-income country, as a reference point. The UCLA PTSD Reaction Indeks for the DSM-5 (PTSD-RI-5) was used for the assessment of traumatic events and PTSD symptoms. Results The most frequently reported traumatic events were death of a close person (69.7%), witnessing violence other than domestic (40.5%), being in a natural disaster (34.4%) and witnessing violent death or serious injury of a close person (33.9%). In total, 28.5% adolescents endorsed two to three DSM-5 PTSD criteria symptoms. The rates of adolescents with symptoms from all four DSM-5 criteria for PTSD were 6.2-8.1% in Indonesia, Serbia, Bulgaria, and Montenegro, and 9.2-10.5% in Philippines, Croatia and Brazil. From Portugal, 10.7% adolescents fall into this category, while 13.2% and 15.3% for the Palestinian Territories and Nigeria, respectively. A logistic regression model showed that younger age, experiencing war, being forced to have indeks, and greater severity of symptoms (persistent avoidance, negative alterations in cognitions and mood, and alterations in arousal and reactivity) were significant predictors of fulfilling full PTSD criteria. Conclusions Nearly every third adolescent living in LMICs might have some PTSD symptoms after experiencing a traumatic event, while nearly one in ten might have sufficient symptoms for full DSM-5 PTSD diagnosis. The findings can inform

		the generation of PTSD burden estimates, allocation of health resources, and designing and implementing psychosocial interventions for PTSD in LMICs.
64.	Fitzpatrick EM, Hamel C, Stevens A, Pratt M, Moher D, Doucet SP, Neuss D, Bernstein A, Na E. Sign Language and Spoken Language for Children With Hearing Loss: A Systematic Review. Pediatrics. 2016 Jan;137(1). doi: 10.1542/peds.2015-1974.	CONTEXT: Permanent hearing loss affects 1 to 3 per 1000 children and interferes with typical communication development. Early detection through newborn hearing screening and hearing technology provide most children with the option of spoken language acquisition. However, no consensus exists on optimal interventions for spoken language development.<br>OBJECTIVE: To conduct a systematic review of the effectiveness of early sign and oral language intervention compared with oral language intervention only for children with permanent hearing loss.<br>DATA SOURCES: An a priori protocol was developed. Electronic databases (eg, Medline, Embase, CINAHL) from 1995 to June 2013 and gray literature sources were searched. Studies in English and French were included.<br>STUDY SELECTION: Two reviewers screened potentially relevant articles.<br>DATA EXTRACTION: Outcomes of interest were measures of auditory, vocabulary, language, and speech production skills. All data collection and risk of bias assessments were completed and then verified by a second person. Grades of Recommendation, Assessment, Development, and Evaluation (GRADE) was used to judge the strength of evidence.<br>RESULTS: Eleven cohort studies metinclusion criteria, of which 8 included only children with severe to profound hearing loss with cochlear implants. Language development was the most frequently reported outcome. Other reported outcomes included speech and speech perception.<br>LIMITATIONS: Several measures and metrics were reported across studies, and descriptions of interventions were sometimes unclear.<br>CONCLUSIONS: Very limited, and hence insufficient, high-quality evidence exists to determine whethersign language in combination with oral language is more effective than oral language therapy alone. More research is needed to supplement the evidence base
	**Peripheral Vascular Disease**	
65.	O'Donnell MJ, Chin SL, Rangarajan S, Xavier D, Liu L, Zhang H, Rao-Melacini P, Zhang X, Pais P, Agapay S, Lopez-Jaramillo P, Damasceno A, Langhorne P, McQueen MJ, Rosengren A, Dehghan M, Hankey GJ, Dans AL, Elsayed A, Avezum A, Mondo C, Diener HC, Ryglewicz D, Czlonkowska A, Pogosova N, Weimar C,	Background Stroke is a leading cause of death and disability, especially in low-income and middle-income countries. We sought to quantify the importance of potentially modifiable risk factors for stroke in different regions of the world, and in key populations and primary pathological subtypes of stroke.<br>Methods We completed a standardised international case-control study in 32 countries in Asia, America, Europe, Australia, the Middle East, and Africa. Cases were patients with

Iqbal R, Diaz R, Yusoff K, Yusufali A, Oguz A, Wang X, Penaherrera E, Lanas F, Ogah OS, Ogunniyi A, Iversen HK, Malaga G, Rumboldt Z, Oveisgharan S, Al Hussain F, Magazi D, Nilanont Y, Ferguson J, Pare G, Yusuf S; INTERSTROKE investigators. Global and regional effects of potentially modifiable risk factors associated with acute stroke in 32 countries (INTERSTROKE): a case-control study. Lancet. 2016 Aug 20;388(10046):761-75. doi: 10.1016/S0140-6736(16)30506-2.	acute first stroke (within 5 days of symptom onset and 72 h of hospital admission). Controls were hospital-based or community-based individuals with no history of stroke, and were matched with cases, recruited in a 1: 1 ratio, for age and indeks. All participants completed a clinical assessment and were requested to provide blood and urine samples. Odds ratios (OR) and their population attributable risks (PARs) were calculated, with 99% confidence intervals.<br>Findings Between Jan 11, 2007, and Aug 8, 2015, 26 919 participants were recruited from 32 countries (13 447 cases [10 388 with ischaemic stroke and 3059 intracerebral haemorrhage] and 13 472 controls). Previous history of hypertension or blood pressure of 140/90 mm Hg or higher (OR 2.98, 99% CI 2.72-3.28; PAR 47.9%, 99% CI 45.1-50.6), regular physical activity (0.60, 0.52-0.70; 35.8%, 27.7-44.7), apolipoprotein (Apo) B/ApoA1 ratio (1.84, 1.65-2.06 for highest vs lowest tertile; 26.8%, 22.2-31.9 for top two tertiles vs lowest tertile), diet (0.60, 0.53-0.67 for highest vs lowest tertile of modifi ed Alternative Healthy Eating Indeks [mAHEI]; 23.2%, 18.2-28.9 for lowest two tertiles vs highest tertile of mAHEI), waist-to-hip ratio (1.44, 1.27-1.64 for highest vs lowest tertile; 18.6%, 13.3-25.3 for top two tertiles vs lowest), psychosocial factors (2.20, 1.78-2.72; 17.4%, 13.1-22.6), current smoking (1.67, 1.49-1.87; 12.4%, 10.2-14.9), cardiac causes (3.17, 2.68-3.75; 9.1%, 8.0-10.2), alcohol consumption (2.09, 1.64-2.67 for high or heavy episodic intake vs 42 san42 r former drinker; 5.8%, 3.4-9.7 for current alcohol drinker vs 42 san42 r former drinker), and diabetes mellitus (1.16, 1.05-1.30; 3.9%, 1.9-7.6) were associated with all stroke. Collectively, these risk factors accounted for 90.7% of the PAR for all stroke worldwide (91.5% for ischaemic stroke, 87.1% for intracerebral haemorrhage), and were consistent across regions (ranging from 82.7% in Africa to 97.4% in southeast Asia), indeks (90.6% in men and in women), and age groups (92.2% in patients aged <= 55 years, 90.0% in patients aged > 55 years). We observed regional variations in the importance of individual risk factors, which were related to variations in the magnitude of Ors (rather than direction, which we observed for diet) and differences in prevalence of risk factors among regions. Hypertension was more associated with intracerebral haemorrhage than with ischaemic stroke, whereas current smoking, diabetes, apolipoproteins, and cardiac causes were more associated with ischaemic stroke (p<0.0001).<br>Interpretation Ten potentially modifiable risk factors are collectively associated with about 90% of the PAR of stroke in each major region of the world, among ethnic groups, in men and women, and in all ages. However, we found important regional variations in the relative importance of most individual risk factors for stroke, which could contribute to

		worldwide variations in frequency and case-mix of stroke. Our findings support developing both global and region-specific programmes to prevent stroke.
66.	Smith NL, Chen MH, Dehghan A, Strachan DP, Basu S, Soranzo N, Hayward C, Rudan I, Sabater-Lleal M, Bis JC, de Maat MP, Rumley A, Kong X, Yang Q, Williams FM, Vitart V, Campbell H, Mälarstig A, Wiggins KL, Van Duijn CM, McArdle WL, Pankow JS, Johnson AD, Silveira A, McKnight B, Uitterlinden AG; Wellcome Trust Case Control Consortium;; Aleksic N, Meigs JB, Peters A, Koenig W, Cushman M, Kathiresan S, Rotter JI, Bovill EG, Hofman A, Boerwinkle E, Tofler GH, Peden JF, Psaty BM, Leebeek F, Folsom AR, Larson MG, Spector TD, Wright AF, Wilson JF, Hamsten A, Lumley T, Witteman JC, Tang W, O'Donnell CJ. Novel associations of multiple genetic loci with plasma levels of factor VII, factor VIII, and von Willebrand factor: The CHARGE (Cohorts for Heart and Aging Research in Genome Epidemiology) Consortium. Circulation. 2010 Mar 30;121(12):1382-92. doi: 10.1161/CIRCULATIONAHA.109.869156. Epub 2010 Mar 15. Erratum in: Circulation.2010 Jul 20;122(3):e399.	Background-Plasma levels of coagulation factors VII (FVII), VIII (FVIII), and von Willebrand factor (vWF) influence risk of hemorrhage and thrombosis. We conducted genome-wide association studies to identify new loci associated with plasma levels.<br>Methods and Results-The setting of the study included 5 community-based studies for discovery comprising 23 608 European-ancestry participants: Atherosclerosis Risk In Communities Study, Cardiovascular Health Study, British 1958 Birth Cohort, Framingham Heart Study, and Rotterdam Study. All subjects had genome-wide single-nucleotide polymorphism (SNP) scans and at least 1 phenotype measured: FVII activity/antigen, FVIII activity, and vWF antigen. Each study used its genotype data to impute to HapMap SNPs and independently conducted association analyses of hemostasis measures using an additive genetic model. Study findings were combined by meta-analysis. Replication was conducted in 7604 participants not in the discovery cohort. For FVII, 305 SNPs exceeded the genome-wide significance threshold of 5.0X10(-8) and comprised 5 loci on 5 chromosomes: 2p23 (smallest P value 6.2X10(-24)), 4q25 (3.6X10(-12)), 11q12 (2.0X10(-10)), 13q34 (9.0X10(-259)), and 20q11.2 (5.7X10(-37)). Loci were within or near genes, including 4 new candidate genes and F7 (13q34). For vWF, 400 SNPs exceeded the threshold and marked 8 loci on 6 chromosomes: 6q24 (1.2X10(-22)), 8p21 (1.3X10(-16)), 9q34 (<5.0X10(-324)), 12p13 (1.7X10(-32)), 12q23 (7.3X10(-10)), 12q24.3 (3.8X10(-11)), 14q32 (2.3X10(-10)), and 19p13.2 (1.3X10(-9)). All loci were within genes, including 6 new candidate genes, as well as ABO (9q34) and VWF (12p13). For FVIII, 5 loci were identified and overlapped vWF findings. Nine of the 10 new findings were replicated.<br>Conclusions-New genetic associations were discovered outside previously known biological pathways and may point to novel prevention and treatment targets of hemostasis disorders. (Circulation. 2010; 121: 1382-1392.)
67.	Peng J, Vongpatanasin W, Sacharidou A, Kifer D, Yuhanna IS, Banerjee S, Tanigaki K, Polasek O, Chu H, Sundgren NC, Rohatgi A, Chambliss KL, Lauc G, Mineo C, Shaul PW. Supplementation With the Sialic Acid Precursor N-Acetyl-D-Mannosamine Breaks the Link Between Obesity and Hypertension. Circulation. 2019 Dec 10;140(24):2005-2018. doi: 10.1161/CIRCULATIONAHA.119.043490.	BACKGROUND: Obesity-related hypertension is a common disorder, and attempts to combat the underlying obesity are often unsuccessful. We previously revealed that mice globally deficient in the inhibitory immunoglobulin G (IgG) receptor Fc gamma RIIB are protected from obesity-induced hypertension. However, how Fc gamma RIIB participates is unknown. Studies were designed to determine if alterations in IgG contribute to the pathogenesis of obesity-induced hypertension.<br>METHODS: Involvement of IgG was studied using IgG mu heavy chain-null mice deficient in mature B cells and by IgG transfer. Participation of Fc gamma RIIB was interrogated in mice with global or endothelial cell-specific deletion of the receptor. Obesity was induced by

		high-fat diet (HFD), and blood pressure (BP) was measured by radiotelemetry or tail cuff. The relative sialylation of the Fc glycan on mouse IgG, which influences IgG activation of Fc receptors, was evaluated by Sambucus nigra lectin blotting. Effects of IgG on endothelial NO synthase were assessed in human aortic endothelial cells. IgG Fc glycan sialylation was interrogated in 3442 human participants by mass spectrometry, and the relationship between sialylation and BP was evaluated. Effects of normalizing IgG sialylation were determined in HFD-fed mice administered the sialic acid precursor N-acetyl-D-mannosamine (ManNAc).<br>RESULTS: Mice deficient in B cells were protected from obesity-induced hypertension. Compared with IgG from control chow-fed mice, IgG from HFD-fed mice was hyposialylated, and it raised BP when transferred to recipients lacking IgG; the hypertensive response was absent if recipients were Fc gamma RIIB-deficient. Neuraminidase-treated IgG lacking the Fc glycan terminal sialic acid also raised BP. In cultured endothelial cells, via Fc gamma RIIB, IgG from HFD-fed mice and neuraminidase-treated IgG inhibited vascular endothelial growth factor activation of endothelial NO synthase by altering endothelial NO synthase phosphorylation. In humans, obesity was associated with lower IgG sialylation, and systolic BP was inversely related to IgG sialylation. Mice deficient in Fc gamma RIIB in endothelium were protected from obesity-induced hypertension. Furthermore, in HFD-fed mice, ManNAc normalized IgG sialylation and prevented obesity-induced hypertension.<br>CONCLUSIONS: Hyposialylated IgG and Fc gamma RIIB in endothelium are critically involved in obesity-induced hypertension in mice, and supportive evidence was obtained in humans. Interventions targeting these mechanisms, such as ManNAc supplementation, may provide novel means to break the link between obesity and hypertension.
	**Pharmacology & Pharmacy**	
68.	Borovac JA, D'Amario D, Vergallo R, Porto I, Bisignani A, Galli M, Annibali G, Montone RA, Leone AM, Niccoli G, Crea F. Neoatherosclerosis after drug-eluting stent implantation: a novel clinical and therapeutic challenge. Eur Heart J Cardiovasc Pharmacother. 2019 Apr 1;5(2):105-116. doi: 10.1093/ehjcvp/pvy036.	The recognition that obstructive disease of the epicardial coronary arteries, causing ischaemic heart disease, can be treated with a percutaneous coronary intervention (PCI) has been a major discovery in cardiology in the last 40 years contributing, in particular, to the reduction of mortality associated to acute myocardial infarction (AMI). However, even in the era of drug-eluting stent (DES) implantation, a sizable proportion of patients who undergo PCI may develop late or very late post-implantation complications, that occur in the form of restenosis, neoatherosclerosis, and/or in-stent thrombosis. Such complications are clinically relevant since they can cause AMI and negatively impact on the outcome. The underlying pathophysiological mechanisms are complex but related to inhibition of neointimal proliferation by DES that, on the hand, reduces the rate of in-stent restenosis,

		but, on the other hand, causes dysfunctional vessel healing, persistent inflammation, platelet activation, and adverse immunological responses. Multiple approaches have been developed or are under evaluation to target DES-related complications including pharmacotherapy, procedure-related imaging methods, novel stent designs, and drug-delivery methods. The aim of this review is to provide an update on the latest preclinical, translational, and clinical pharmacotherapeutic developments in this setting that target novel cellular mechanisms and pathways that might contribute to neoatherosclerosis. Due to the importance of secondary prevention in the reduction of DES-associated complications, this review also provides a short overview of pharmacological agents that are established or currently being investigated in this regard.
	**Psychiatry**	
69.	Allebrandt KV, Amin N, Müller-Myhsok B, Esko T, Teder-Laving M, Azevedo RV, Hayward C, van Mill J, Vogelzangs N, Green EW, Melville SA, Lichtner P, Wichmann HE, Oostra BA, Janssens AC, Campbell H, Wilson JF, Hicks AA, Pramstaller PP, Dogas Z, Rudan I, Merrow M, Penninx B, Kyriacou CP, Metspalu A, van Duijn CM, Meitinger T, Roenneberg T. A K(ATP) channel gene effect on sleep duration: from genome-wide association studies to function in Drosophila. Mol Psychiatry. 2013 Jan;18(1):122-32. doi: 10.1038/mp.2011.142.	Humans sleep approximately a third of their lifetime. The observation that individuals with either long or short sleep duration show associations with metabolic syndrome and psychiatric disorders suggests that the length of sleep is adaptive. Although sleep duration can be influenced by photoperiod (season) and phase of entrainment (chronotype), human familial sleep disorders indicate that there is a strong genetic modulation of sleep. Therefore, we conducted high-density genome-wide association studies for sleep duration in seven European populations (N=4251). We identified an intronic variant (rs11046205; P=3.99 x 10(-8)) in the ABCC9 gene that explains approximate to 5% of the variation in sleep duration. An influence of season and chronotype on sleep duration was solely observed in the replication sample (N=5949). Meta-analysis of the associations found in a subgroup of the replication sample, chosen for season of entry and chronotype, together with the discovery results showed genome-wide significance. RNA interference knockdown experiments of the conserved ABCC9 homologue in Drosophila neurons renders flies sleepless during the first 3 h of the night. ABCC9 encodes an ATP-sensitive potassium channel subunit (SUR2), serving as a sensor of intracellular energy metabolism. Molecular Psychiatry (2013) 18, 122-132; doi:10.1038/mp.2011.142; published online 22 November 2011
70.	Signorini G, Singh SP, Boricevic-Marsanic V, Dieleman G, Dodig-Ćurković K, Franic T, Gerritsen SE, Griffin J, Maras A, McNicholas F, O'Hara L, Purper-Ouakil D, Paul M, Santosh P, Schulze U, Street C, Tremmery S, Tuomainen H, Verhulst F, Warwick J, de Girolamo G; MILESTONE Consortium. Architecture and functioning of child and adolescent mental	The WHO Child and Adolescent Mental Health Atlas, published in 2005, reported that child and adolescent mental health services (CAMHS) in Europe differed substantially in their architecture and functioning. We assessed the characteristics of national CAMHS across the European Union (EU), including legal aspects of adolescent care. Using an online mapping survey aimed at expert(s) in each country, we obtained data for all 28 countries in the EU. The characteristics and activities of CAMHS (ie, availability of services, inpatient beds, and clinicians and organisations, and delivery of specific CAMHS services and

	health services: a 28-country survey in Europe. Lancet Psychiatry. 2017 Sep;4(9):715-724. doi: 10.1016/S2215-0366(17)30127-X. Epub 2017 Jun 6. Erratum in: Lancet Psychiatry. 2017 Dec;4(12):e29. doi: 10.1016/S2215-0366(17)30452-2. Erratum in: Lancet Psychiatry. 2018 May;5(5):e10. doi: 10.1016/S2215-0366(18)30099-3. Erratum in: Lancet Psychiatry. 2019 Jul;6(7):e16. doi: 10.1016/S2215-0366(19)30240-8.	treatments) varied considerably between countries, as did funding sources and user access. Neurodevelopmental disorders were the most frequent diagnostic group (up to 81%) for people seen at CAMHS (data available from only 13 [46%] countries). 20 (70%) countries reported having an official national child and adolescent mental health policy, covering young people until their official age of transition to adulthood. The heterogeneity in resource allocation did not seem to match epidemiological burden. Substantial improvements in the planning, monitoring, and delivery of mental health services for children and adolescents are needed.
71.	Hinney A, Kesselmeier M, Jall S, Volckmar AL, Föcker M, Antel J; GCAN; WTCCC3; Heid IM, Winkler TW; GIANT; Grant SF; EGG; Guo Y, Bergen AW, Kaye W, Berrettini W, Hakonarson H; Price Foundation Collaborative Group; Children's Hospital of Philadelphia/Price Foundation; Herpertz-Dahlmann B, de Zwaan M, Herzog W, Ehrlich S, Zipfel S, Egberts KM, Adan R, Brandys M, van Elburg A, Boraska Perica V, Franklin CS, Tschöp MH, Zeggini E, Bulik CM, Collier D, Scherag A, Müller TD, Hebebrand J. Evidence for three genetic loci involved in both anorexia nervosa risk and variation of body mass indeks. Mol Psychiatry. 2017 Feb;22(2):192-201. doi: 10.1038/mp.2016.71. Epub 2016 May 17. Erratum in: Mol Psychiatry. 2017 Feb;22(2):321-322. doi: 10.1038/mp.2016.126.	The maintenance of normal body weight is disrupted in patients with anorexia nervosa (AN) for prolonged periods of time. Prior to the onset of AN, premorbid body mass indeks (BMI) spans the entire range from underweight to obese. After recovery, patients have reduced rates of overweight and obesity. As such, loci involved in body weight regulation may also be relevant for AN and vice versa. Our primary analysis comprised a cross-trait analysis of the 1000 single-nucleotide polymorphisms (SNPs) with the lowest Pvalues in a genome-wide association meta-analysis (GWAMA) of AN (GCAN) for evidence of association in the largest published GWAMA for BMI (GIANT). Subsequently we performed indeks-stratified analyses for these 1000 SNPs. Functional ex vivo studies on four genes ensued. Lastly, a look-up of GWAMA-derived BMI-related loci was performed in the AN GWAMA. We detected significant associations (P-values <5x10(-5), Bonferroni-corrected P<0.05) for nine SNP alleles at three independent loci. Interestingly, all AN susceptibility alleles were consistently associated with increased BMI. None of the genes (chr. 10: CTBP2, chr. 19: CCNE1, chr. 2: CARF and NBEAL1; the latter is a region with high linkage disequilibrium) nearest to these SNPs has previously been associated with AN or obesity. Indeks-stratified analyses revealed that the strongest BMI signal originated predominantly from females (chr. 10 rs1561589; P-overall: 2.47 x 10(-06)/P-females: 3.45 x 10(-07)/P-males: 0.043). Functional ex vivo studies in mice revealed reduced hypothalamic expression of Ctbp2 and Nbeal1 after fasting. Hypothalamic expression of Ctbp2 was increased in diet-induced obese (DIO) mice as compared with age-matched lean controls. We observed no evidence for associations for the look-up of BMI-related loci in the AN GWAMA. A cross-trait analysis of AN and BMI loci revealed variants at three chromosomal loci with potential joint impact. The chromosome 10 locus is particularly promising given that the association with obesity was primarily driven by females. In addition, the detected altered hypothalamic expression patterns of Ctbp2 and Nbeal1 as a result of fasting and DIO implicate these genes in weight regulation.

72.	Gerritsen SE, van Bodegom LS, Overbeek MM, Maras A, Verhulst FC, Wolke D, Rizopoulos D, de Girolamo G, Franić T, Madan J, McNicholas F, Paul M, Purper-Ouakil D, Santosh PJ, Schulze UME, Singh SP, Street C, Tremmery S, Tuomainen H, Dieleman GC; MILESTONE consortium. Leaving child and adolescent mental health services in the MILESTONE cohort: a longitudinal cohort study on young people's mental health indicators, care pathways, and outcomes in Europe. Lancet Psychiatry. 2022 Dec;9(12):944-956. doi: 10.1016/S2215-0366(22)00310-8.	Background The configuration of having separate mental health services by age, namely child and adolescent mental health services (CAMHS) and adult mental health services (AMHS), might be a barrier to continuity of care that adversely affects young people's mental health. However, no studies have investigated whether discontinuity of care in the transition period affects mental health. We aimed to discern the type of care young people receive after reaching the upper age limit of their CAMHS and examine differences in outcomes at 24-month follow-up between young people receiving different types of care.Methods To assess mental health in young people from 39 CAMHS in eight European countries (Belgium, Croatia, France, Germany, Italy, Ireland, the Netherlands, and the UK), we did a longitudinal cohort study. Eligible young people were CAMHS users up to 1 year younger than the upper age limit of their CAMHS 47 san to 3 months older, if they were still in CAMHS. Information on mental health service use, mental health problems (ie, using the Health of the Nation Outcome Scale for Children and Adolescents, Youth Self-Report and Adult Self-Report, DSM-5, and ICD-10), and sociodemographic characteristics were collected using self-reported, parent-reported, and clinician -reported interviews and questionnaires. Mixed models were applied to assess relationships between baseline characteristics, mental health service use, and outcomes.Findings The MILESTONE cohort included 763 young people. The participants were 60middot0% female (n=458) and 40middot0% male (n=305), 90middot3% White (n=578), and had a mean age of 17middot5 years (range 15middot2-19middot6 years). Over the 24-month follow-up period, 48 young people (6middot3%) actively withdrew from the study. For young people, the higher their scores on the Health of the Nation Outcome Scale for Children and Adolescents (p=0middot0009) and Youth Self -Report and Adult Self-Report (p=0middot046), and who had a clinical classification of severe mental illness (p=0middot0033), had suicidal thoughts or behaviours or self-harm (p=0middot034), used psychotropic medication (p=0middot0014), and had a self -reported or parent-reported need for continued treatment (p < 0middot0001) at baseline, were more likely to transition to AMHS or stay in CAMHS than to have care end. Overall, over the 24-month follow-up period, the mental health of young people improved, but 24middot4% of young people reported an increase in problems calculated using the reliable change indeks, of whom 5middot3% had a clinically relevant increase in problems. At 24-month follow-up, no differences in change in mental health problems since baseline were found between young people who used different types of care (CAMHS, AMHS, or no care).Interpretation Although approximately half of young people reaching the upper age limit of their CAMHS stop using mental health services, this was not associated with a deterioration in their mental health. Young people

		with the most severe mental health problems are more likely to receive continued care. If replicated, our findings suggest investments in improving transitional care for all CAMHS users might not be cost-effective in times of rising health-care costs, but might be better targeted at a subgroup of young people with increasing mental health problems who do not receive continued treatment.Funding European Commission's 7th Framework Programme.
	**Public, Environmental & Occupational**	
73.	McKeigue PM, Campbell H, Wild S, Vitart V, Hayward C, Rudan I, Wright AF, Wilson JF. Bayesian methods for instrumental variable analysis with genetic instruments ('Mendelian randomization'): example with urate transporter SLC2A9 as an instrumental variable for effect of urate levels on metabolic syndrome. Int J Epidemiol. 2010 Jun;39(3):907-18. doi: 10.1093/ije/dyp397.	The 'Mendelian randomization' approach uses genotype as an instrumental variable to distinguish between causal and non-causal explanations of biomarker-disease associations. Classical methods for instrumental variable analysis are limited to linear or probit models without latent variables or missing data, rely on asymptotic approximations that are not valid for weak instruments and focus on estimation rather than hypothesis testing. We describe a Bayesian approach that overcomes these limitations, using the JAGS program to compute the log-likelihood ratio (lod score) between causal and non-causal explanations of a biomarker-disease association. To demonstrate the approach, we examined the relationship of plasma urate levels to metabolic syndrome in the ORCADES study of a Scottish population isolate, using genotype at six single-nucleotide polymorphisms in the urate transporter gene SLC2A9 as an instrumental variable. In models that allow for intra-individual variability in urate levels, the lod score favouring a non-causal over a causal explanation was 2.34. In models that do not allow for intra-individual variability, the weight of evidence against a causal explanation was weaker (lod score 1.38). We demonstrate the ability to test one of the key assumptions of instrumental variable analysis-that the effects of the instrument on outcome are mediated only through the intermediate variable-by constructing a test for residual effects of genotype on outcome, similar to the tests of 'overidentifying restrictions' developed for classical instrumental variable analysis. The Bayesian approach described here is flexible enough to deal with any instrumental variable problem, and does not rely on asymptotic approximations that may not be valid for weak instruments. The approach can easily be extended to combine information from different study designs. Statistical power calculations show that instrumental variable analysis with genetic instruments will typically require combining information from moderately large cohort and cross-sectional studies of biomarkers with information from very large genetic case-control studies.
74.	Rudan I, Chan KY, Zhang JS, Theodoratou E, Feng XL, Salomon JA, Lawn JE, Cousens S, Black RE, Guo Y, Campbell H; WHO/UNICEF's Child Health	Background Previous estimates of the global burden of disease for children have not included much information from China, leading to a large gap in data. We identified the main causes of deaths in neonates (<1 month), postneonatal infants (1-11 months), and

	Epidemiology Reference Group (CHERG). Causes of deaths in children younger than 5 years in China in 2008. Lancet. 2010 Mar 27;375(9720):1083-9. doi: 10.1016/S0140-6736(10)60060-8. Erratum in: Lancet. 2010 May 15;375(9727):1694.	children (<5 years) in China using information that was available to the public.<br>Methods The Child Health Epidemiology Reference Group in collaboration with colleagues from Peking University systematically searched Chinese databases that were available to the public. Information was obtained from the Chinese Ministry of Health and Bureau of Statistics websites, Chinese National Knowledge Infrastructure database, and Chinese Health Statistics yearbooks for 1990-2008. We also obtained information from 206 high-quality community-based longitudinal studies of different causes of deaths in children (<5 years) that were written in the Chinese language. A statistical model was developed to estimate the total number of deaths in children according to provinces, age groups, and main causes.<br>Findings During 1990-2008, the mortality rates in neonates, postneonatal infants, and children were reduced by 70% (from 34.0 to 10.2 per 1000 livebirths), 72% (from 53.5 to 14.9 per 1000 livebirths), and 71% (from 64.6 to 18.5 per 1000 livebirths), respectively, meeting the targets set in the Millennium Development Goal 4. The leading causes of deaths in 2008 were pneumonia, birth asphyxia, and preterm birth complications, each accounting for 15-17% of all deaths. Congenital abnormalities and accidents increased in importance during this period, contributing to 11% and 10% of child deaths, respectively. Sudden infant death syndrome contributed to 5% of deaths in children.<br>Interpretation Publically available Chinese databases contain much important information that has been underused in the estimation of global and regional burden of disease. On the basis of trends, preterm birth complications are expected to become the leading cause of child mortality in China, whereas deaths from congenital abnormalities, accidents, and sudden infant death syndrome are predicted to continue increasing in importance in the long term.
75.	Bućan K, Matas A, Lovrić JM, Batistić D, Pleština Borjan I, Puljak L, Bućan I. Epidemiology of ocular trauma in children requiring hospital admission: a 16-year retrospective cohort study. J Glob Health. 2017 Jun;7(1):010415. doi: 10.7189/jogh.07.010415.	Background To study the epidemiology of ocular trauma requiring hospital admission in children under 18 years in age.<br>Methods This retrospective cohort study included pediatric patients with ocular injuries at the Ophthalmology Department of the Clinical Hospital Centre, Split, Croatia, from 2000 to 2015, classified according to the Birmingham Eye Trauma Terminology.<br>Results There were 353 children hospitalized, 82% of boys (mean age 11 years) and 18% of girls (mean age 10 years). The majority of traumas occurred in the outside environment (70%, n = 249), followed by occurrences at home (17%, n = 60), and at a school/nursery (8%, n = 28). Final visual acuity was 6/18 or better in 286 (96%) patients with closed globe injury and in 26 (49%) patients with open globe injury. Severe impairment of vision was found in 12 (4.4%) patients in the closed globe injury group and 26 (49%) patients in the

		open globe injury group. A statistically significant difference was found between final visual acuity and initial visual acuity in all patients (x(2) = 12.8; P<0.001).<br>Conclusion The majority of pediatric eye injuries are happening in the outside environment and are preventable. Implementation of well-established safety precautions would greatly reduce this source of visual disability in children.
76.	Song P, Wang J, Bucan K, Theodoratou E, Rudan I, Chan KY. National and subnational prevalence and burden of glaucoma in China: A systematic analysis. J Glob Health. 2017 Dec;7(2):020705. doi: 10.7189/jogh.07.020705.	Background Glaucoma, the second leading cause of blindness, affects approximately 64.3 million individuals worldwide. In China, demographic ageing is in rapid progress. Yet detailed and up-to-date estimates of the scale of glaucoma are rare. We aimed to quantify and understand the prevalence and burden of glaucoma in China from 1990 to 2015, with projections until 2050.<br>Methods For this systematic review and meta-analysis, we searched China National Knowledge Infrastructure (CNKI), Wanfang, Chinese Biomedicine Literature Database (CBM-SinoMed), PubMed, Embase and Medline using comprehensive search strategies to identify all relevant articles that have reported the prevalence of glaucoma in the general Chinese population. We used a multilevel mixed-effect meta-regression to estimate the prevalence rates of primary open-angle glaucoma (POAG) and primary angle-closure glaucoma (PACG), and a random-effects meta-analysis to pool the overall prevalence of secondary glaucoma. United Nations population data were used to estimate and project the number of people with glaucoma from 1990 to 2050. Univariable and multivariable meta-regressions were conducted to assess the association between the prevalence of POAG and PACG and relevant demographic and geographic factors. The national burden of POAG and PACG in the years 2000 and 2010 were distributed to six geographic regions accordingly.<br>Results From 1990 to 2015, the prevalence of all glaucoma ranged from 2.59% (95% CI = 1.96-3.49) to 2.58% (95% CI = 1.94-3.47). For different subtypes of glaucoma, the overall prevalence of POAG ranged from 1.03% (95% CI = 0.67-1.58) in 1990 to 1.02% (95% CI = 0.67-1.57) in 2015, PACG from 1.41% (95% CI = 1.18-1.68) to 1.40% (95% CI = 1.17-1.68). The overall prevalence of secondary glaucoma was 0.15% (95% CI = 0.10-0.23) during this period. The number of people with all glaucoma in China was 5.92 million (95% CI = 4.47-7.97) in 1990, and 13.12 million (95% CI = 9.88-17.68) in 2015. This increasing trend was also witnessed in different subtypes of glaucoma. The number of people affected by POAG increased from 2.35 million (95% CI = 1.54-3.60) in 1990 to 5.22 million (95% CI = 3.40-7.98) in 2015, PACG from 3.22 million (95% CI = 2.70-3.84) to 7.14 million (95% CI = 5.97-8.53), and secondary glaucoma from 0.34 million (95% CI = 0.23-0.53) to 0.76 million (95% CI = 0.51-1.17). In 2015, more than half (54.42%) of the glaucoma cases were PACG,

		followed by POAG (39.79%) and secondary glaucoma (5.79%). By 2050, the number of all glaucoma cases in China will be 25.16 million (95% CI = 18.96-33.86). In the multivariable meta-regressions, the odds ratio (OR) for each decade's increase in age was 1.43 (95% CI = 1.33-1.55) for POAG, and 1.65 (95% CI = 1.51-1.80) for PACG; males were more likely to have POAG (OR 1.36, 95% CI = 1.17-1.59), but less likely to have PACG (OR 0.53, 95% CI = 0.46-0.60) compared with females. After adjustment of age and gender, people living in urban areas were more likely to have POAG compared with those in rural areas (OR 1.54, 95% CI = 1.02-2.35). People in Northeast China were at a higher risk (OR 1.77, 95% CI = 1.07-2.94) of having PACG than people in East China. Among the six regions, East China owed the most POAG and PACG cases, whereas Northwest China owed the least. Conclusions This systematic review and meta-analysis suggests a substantial burden of glaucoma in China, with great variances among the different age groups, genders, settings and geographic regions. With the dramatic ageing trend in the next three decades, the prevalence and burden of glaucoma will continue to increase. More elaborate epidemiological studies are needed to optimise public health strategies for mitigating this important health problem.
77.	GAPPS Expert Group on Community Based Strategies and Constraints; George A, Young M, Bang A, Chan KY, Rudan I, Victora CG, Chopra M, Rubens C. Setting implementation research priorities to reduce preterm births and stillbirths at the community level. PloS Med. 2011 Jan 4;8(1):e1000380. doi: 10.1371/journal.pmed.1000380.	Asha George and colleagues from the GAPPS group report the implementation research priorities to address prematurity and stillbirths at the community level that resulted from their recent expert consensus exercise.
78.	Lawn JE, Bahl R, Bergstrom S, Bhutta ZA, Darmstadt GL, Ellis M, English M, Kurinczuk JJ, Lee AC, Merialdi M, Mohamed M, Osrin D, Pattinson R, Paul V, Ramji S, Saugstad OD, Sibley L, Singhal N, Wall SN, Woods D, Wyatt J, Chan KY, Rudan I. Setting research priorities to reduce almost one million deaths from birth asphyxia by 2015. PloS Med. 2011 Jan 11;8(1):e1000389. doi: 10.1371/journal.pmed.1000389.	Joy Lawn and colleagues used a systematic process developed by the Child Health Nutrition Research Initiative (CHNRI) to define and rank research options to reduce mortality from intrapartum-related neonatal deaths (birth asphyxia) by the year 2015.
79.	Fontaine O, Kosek M, Bhatnagar S, Boschi-Pinto C, Chan KY, Duggan C, Martinez H, Ribeiro H, Rollins NC, Salam MA, Santosham M, Snyder JD, Tsai AC,	Olivier Fontaine and colleagues applied a priority-setting methodology to identify research priorities aimed at reducing global diarrhea mortality by 2015.

	Vargas B, Rudan I. Setting research priorities to reduce global mortality from childhood diarrhoea by 2015. PloS Med. 2009 Mar 10;6(3):e41. doi: 10.1371/journal.pmed.1000041.	
80.	Rudan I, El Arifeen S, Bhutta ZA, Black RE, Brooks A, Chan KY, Chopra M, Duke T, Marsh D, Pio A, Simoes EA, Tamburlini G, Theodoratou E, Weber MW, Whitney CG, Campbell H, Qazi SA; WHO/CHNRI Expert Group on Childhood Pneumonia. Setting research priorities to reduce global mortality from childhood pneumonia by 2015. PloS Med. 2011 Sep;8(9):e1001099. doi: 10.1371/journal.pmed.1001099.	Igor Rudan and colleagues report the results of their consensus building exercise that identified health research priorities to help reduce child mortality from pneumonia.
81.	Pattinson R, Kerber K, Buchmann E, Friberg IK, Belizan M, Lansky S, Weissman E, Mathai M, Rudan I, Walker N, Lawn JE; Lancet's Stillbirths Series steering committee. Stillbirths: how can health systems deliver for mothers and babies? Lancet. 2011 May 7;377(9777):1610-23. doi: 10.1016/S0140-6736(10)62306-9.	The causes of stillbirths are inseparable from the causes of maternal and neonatal deaths. This report focuses on prevention of stillbirths by scale-up of care for mothers and babies at the health-system level, with consideration for effects and cost. In countries with high mortality rates, emergency obstetric care has the greatest effect on maternal and neonatal deaths, and on stillbirths. Syphilis detection and treatment is of moderate effect but of lower cost and is highly feasible. Advanced antenatal care, including induction for post-term pregnancies, and detection and management of hypertensive disease, fetal growth restriction, and gestational diabetes, will further reduce mortality, but at higher cost. These interventions are best packaged and provided through linked service delivery methods tailored to suit existing health-care systems. If 99% coverage is reached in 68 priority countries by 2015, up to 1.1 million (45%) third-trimester stillbirths, 201000 (54%) maternal deaths, and 1.4 million (43%) neonatal deaths could be saved per year at an additional total cost of US$10.9 billion or $2.32 per person, which is in the range of $0.96-2.32 for other ingredients-based intervention packages with only recurrent costs.
82.	Theodoratou E, Al-Jilaihawi S, Woodward F, Ferguson J, Jhass A, Balliet M, Kolcic I, Sadruddin S, Duke T, Rudan I, Campbell H. The effect of case management on childhood pneumonia mortality in developing countries. Int J Epidemiol. 2010 Apr;39 Suppl 1(Suppl 1):i155-71. doi: 10.1093/ije/dyq032.	Background With the aim of populating the Lives Saved Tool (LiST) with parameters of effectiveness of existing interventions, we conducted a systematic review of the literature assessing the effect of pneumonia case management on mortality from childhood pneumonia.<br>Methods This review covered the following interventions: community case management with antibiotic treatment, and hospital treatment with antibiotics, oxygen, zinc and vitamin A. Pneumonia mortality outcomes were sought where available but data were also recorded on secondary outcomes. We summarized results from randomized controlled

		trials (RCTs), cluster RCTs, quasi-experimental studies and observational studies across outcome measures using standard meta-analysis methods and used a set of standardized rules developed for the purpose of populating the LiST with required parameters, which dealt with the issues of comparability of the studies in a uniform way across a spectrum of childhood conditions.<br>Results We estimate that community case management of pneumonia could result in a 70% reduction in mortality from pneumonia in 0-5-year-old children. In contrast treatment of pneumonia episodes with zinc and vitamin A is ineffective in reducing pneumonia mortality. There is insufficient evidence to make a quantitative estimate of the effect of hospital case management on pneumonia mortality based on the published data.<br>Conclusion The available evidence reinforces the effectiveness of community and hospital case management with World Health Organization-recommended antibiotics and the lack of effect of zinc and vitamin A supportive treatment for children with pneumonia. Evidence from one trial demonstrates the effectiveness of oxygen therapy but further research is required to give higher quality evidence so that an effect estimate can be incorporated into the LIST model. We identified no trials that separately evaluated the effectiveness of other supportive care interventions. The summary estimates of effect on pneumonia mortality will inform the LiST model.
83.	Theodoratou E, Johnson S, Jhass A, Madhi SA, Clark A, Boschi-Pinto C, Bhopal S, Rudan I, Campbell H. The effect of Haemophilus influenzae type b and pneumococcal conjugate vaccines on childhood pneumonia incidence, severe morbidity and mortality. Int J Epidemiol. 2010 Apr;39 Suppl 1(Suppl 1):i172-85. doi: 10.1093/ije/dyq033.	Background With the aim of populating the Lives Saved Tool (LiST) with parameters of effectiveness of existing interventions, we conducted a systematic review of the literature assessing the effect of Haemophilus influenzae type b (Hib) and pneumococcal (PC) conjugate vaccines on incidence, severe morbidity and mortality from childhood pneumonia.<br>Methods We summarized cluster randomized controlled trials (cRCTs) and case control studies of Hib conjugate vaccines and RCTs of 9- and 11-valent PC conjugate vaccines conducted in developing countries across outcome measures using standard meta-analysis methods. We used a set of standardized rules developed for the purpose of populating the LiST tool with required parameters to promote comparability across reviews of interventions against the major causes of childhood mortality. The estimates could be adjusted further to account for factors such as PC vaccine serotype content, PC serotype distribution and human immunodeficiency virus prevalence but this was not included as part of the LIST model approach.<br>Results The available evidence from published data points to a summary effect of the Hib conjugate vaccine on clinical pneumonia of 4%, on clinical severe pneumonia of 6% and on radiologically confirmed pneumonia of 18%. Respective effectiveness estimates for PC

		vaccines (all valent) on clinical pneumonia is 7%, clinical severe pneumonia is 7% and radiologically confirmed pneumonia is 26%.<br>Conclusions The findings indicated that radiologically confirmed pneumonia, as a severe morbidity proxy for mortality, provided better estimates for the LiST model of effect of interventions on mortality reduction than did other outcomes evaluated. The LiST model will use this to estimate the pneumonia mortality reduction which might be observed when scaling up Hib and PC conjugate vaccination in the context of an overall package of child health interventions.
84.	Culić V, Eterović D, Mirić D, Giunio L, Lukin A, Fabijanić D. Triggering of ventricular tachycardia by meteorologic and emotional stress: protective effect of beta-blockers and anxiolytics in men and elderly. Am J Epidemiol. 2004 Dec 1;160(11):1047-58. doi: 10.1093/aje/kwh335.	A circadian pattern with a morning peak and the triggering role of emotional stress have been suggested for ventricular arrhythmias. After controlling for participant baseline characteristics and medication used, the authors studied the association of emotional upset, physical activity, and meteorologic parameters with occurrence of ventricular tachycardia (VT) in 457 Croatian participants aged 11-88 years consecutively assigned to undergo continuous 24-hour Holter monitoring. In 2001, multivariate analysis of possible VT precipitators was performed separately for men, women, those aged <65 years, and those aged >64 years. A U-shaped pattern of wind speed (either very weak or very strong), rising relative air moisture, falling atmospheric pressure, and emotional upset were independent predictors of VT episodes in all participant subgroups. Positive association of VT with higher atmospheric temperature or pressure was observed in women and elderly. After adjustment for external triggers, a circadian variation in VT episodes persisted in women ($p = 0.01$) and those aged <65 years ($p < 0.0001$) only. A protective effect of beta-blockers and anxiolytics was especially apparent for men and elderly, as well as an adverse effect of digitalis in women. Results suggest that meteorologic and emotional stress could be considered external triggers of VT, with age- and indeks-dependent susceptibility.
85.	AMANHI (Alliance for Maternal and Newborn Health Improvement) Bio–banking Study group); Baqui AH, Khanam R, Rahman MS, Ahmed A, Rahman HH, Moin MI, Ahmed S, Jehan F, Nisar I, Hussain A, Ilyas M, Hotwani A, Sajid M, Qureshi S, Zaidi A, Sazawal S, Ali SM, Deb S, Juma MH, Dhingra U, Dutta A, Ame SM, Hayward C, Rudan I, Zangenberg M, Russell D, Yoshida S, Polašek O, Manu A, Bahl R. Understanding biological mechanisms underlying adverse birth outcomes in developing countries: protocol for a prospective cohort (AMANHI bio-banking) study. J	Objectives The AMANHI study aims to seek for biomarkers as predictors of important pregnancy-related outcomes, and establish a biobank in developing countries for future research as new methods and technologies become available.<br>Methods AMANHI is using harmonised protocols to enrol 3000 women in early pregnancies (8-19 weeks of gestation) for population-based follow-up in pregnancy up to 42 days postpartum in Bangladesh, Pakistan and Tanzania, with collection taking place between August 2014 and June 2016. Urine pregnancy tests will be used to confirm reported or suspected pregnancies for screening ultrasound by trained sonographers to accurately date the pregnancy. Trained study field workers will collect very detailed phenotypic and epidemiological data from the pregnant woman and her family at scheduled home visits during pregnancy (enrolment, 24-28 weeks, 32-36 weeks & 38+

	Glob Health. 2017 Dec;7(2):021202. doi: 10.7189/jogh.07.021202.	weeks) and postpartum (days 0-6 or 42-60). Trained phlebotomists will collect maternal and umbilical blood samples, centrifuge and obtain aliquots of serum, plasma and the buffy coat for storage. They will also measure HbA1C and collect a dried spot sample of whole blood. Maternal urine samples will also be collected and stored, alongside placenta, umbilical cord tissue and membrane samples, which will both be frozen and prepared for histology examination. Maternal and newborn stool (for microbiota) as well as paternal and newborn saliva samples (for DNA extraction) will also be collected. All samples will be stored at -80 degrees C in the biobank in each of the three sites. These samples will be linked to numerous epidemiological and phenotypic data with unique study identification numbers.<br>Importance of the study AMANHI biobank proves that biobanking is feasible to implement in LMICs, but recognises that biobank creation is only the first step in addressing current global challenges.
	**Radiology, Nuclear Medicine & Imaging**	
86.	Vrdoljak E, Prskalo T, Omrcen T, Situm K, Boraska T, Frleta Ilić N, Janković S, Hamm W. Concomitant chemobrachyradiotherapy with ifosfamide and cisplatin followed by consolidation chemotherapy in locally advanced squamous cell carcinoma of the uterine cervix: results of a phase II study. Int J Radiat Oncol Biol Phys. 2005 Mar 1;61(3):824-9. doi: 10.1016/j.ijrobp.2004.06.248.	Purpose: To evaluate the efficacy and toxicity of ifosfamide and cisplatin administered concomitantly with low-dose-rate brachytherapy followed by consolidation chemotherapy in the treatment of locally advanced squamous cell cervical carcinoma (LASCC).<br>Methods and Materials: Forty-four patients with biopsy-proven LASCC were enrolled. FIGO Stages 1132 bulky to IVA were entered into this study. Patients were assigned to receive external radiotherapy (50 Gy in 25 fractions); then ifosfamide 2 g/m(2) Plus cisplatin 75 mg/m(2) was applied during two low-dose-rate brachytherapy applications, and 4 cycles of consolidation chemotherapy with the same drug combination were given after completion of radiotherapy. The planned dose to point A was 85 Gy.<br>Results: All patients received both courses of concomitant chemobrachytherapy and at least 1 cycle of consolidation chemotherapy. The average duration of radiation was 45.1 days. The clinical complete response rate was 100%. Grade 3 and 4 leukopenia occurred in 25 % and 11% of the cycles, respectively. After a median follow-up of 34 months (range, 20-54 months), the recurrence-free and the overall survival rates were 84% and 91%, respectively. Major delayed local complications occurred in 7 cases (16%).<br>Conclusions: These results indicate that concomitant chemobrachyradiotherapy with ifosfamide and cisplatin is a feasible combination for patients with LASCC of the cervix uteri. A randomized trial is planned. INDEKS 2005 Elsevier Inc.
87.	Eterovic D, Antunovic Z, Markovic V, Grosev D. Planning of 131I therapy for graves disease based on the radiation dose to thyroid follicular cells. J Nucl	We evaluated the effects on the absorbed dose to thyroid follicular cells of self-absorption of I-131 radiation (specifically, beta-rays) in the follicular colloid. Methods: Thyroid follicles were modeled as colloid-filled spheres, containing a uniform concentration of I-131 and

	Med. 2008 Dec;49(12):2026-30. doi: 10.2967/jnumed.108.053934.	surrounded by a concentric monolayer of cells. Assuming close packing of identical follicles, we used Monte Carlo simulation to assess the absorbed dose to follicular cells. Results: Because of beta-ray self-absorption in colloidal spheres with radii larger than 50 mu m, the absorbed dose to follicular cells is less than the average thyroid absorbed dose. Conclusion: For the same thyroid mass, radioiodine thyroid uptake, and effective half-life, patients with follicles with colloidal sphere radii of 100, 200, 300, and 400 mu m should be administered 9%, 15%, 21 %, and 30% more I-131, respectively, than patients with colloidal sphere radii of less than 50 mu m, to yield the same absorbed dose to follicular cells.
	**Respiratory System**	
88.	Obeidat M, Hao K, Bossé Y, Nickle DC, Nie Y, Postma DS, Laviolette M, Sandford AJ, Daley DD, Hogg JC, Elliott WM, Fishbane N, Timens W, Hysi PG, Kaprio J, Wilson JF, Hui J, Rawal R, Schulz H, Stubbe B, Hayward C, Polasek O, Järvelin MR, Zhao JH, Jarvis D, Kähönen M, Franceschini N, North KE, Loth DW, Brusselle GG, Smith AV, Gudnason V, Bartz TM, Wilk JB, O'Connor GT, Cassano PA, Tang W, Wain LV, Soler Artigas M, Gharib SA, Strachan DP, Sin DD, Tobin MD, London SJ, Hall IP, Paré PD. Molecular mechanisms underlying variations in lung function: a systems genetics analysis. Lancet Respir Med. 2015 Oct;3(10):782-95. doi: 10.1016/S2213-2600(15)00380-X. Epub 2015 Sep 21. Erratum in: Lancet Respir Med. 2015 Dec;3(12):e44. doi: 10.1016/S2213-2600(15)00478-6.	Background Lung function measures reflect the physiological state of the lung, and are essential to the diagnosis of chronic obstructive pulmonary disease (COPD). The SpiroMeta-CHARGE consortium undertook the largest genome-wide association study (GWAS) so far (n=48 201) for forced expiratory volume in 1 s (FEV1) and the ratio of FEV1 to forced vital capacity (FEV1/FVC) in the general population. The lung expression quantitative trait loci (eQTLs) study mapped the genetic architecture of gene expression in lung tissue from 1111 individuals. We used a systems genetics approach to identify single nucleotide polymorphisms (SNPs) associated with lung function that act as eQTLs and change the level of expression of their target genes in lung tissue; termed eSNPs.<br>Methods The SpiroMeta-CHARGE GWAS results were integrated with lung eQTLs to map eSNPs and the genes and pathways underlying the associations in lung tissue. For comparison, a similar analysis was done in peripheral blood. The lung mRNA expression levels of the eSNP-regulated genes were tested for associations with lung function measures in 727 individuals. Additional analyses identified the pleiotropic effects of eSNPs from the published GWAS catalogue, and mapped enrichment in regulatory regions from the ENCODE project. Finally, the Connectivity Map database was used to identify potential therapeutics in silico that could reverse the COPD lung tissue gene signature.<br>Findings SNPs associated with lung function measures were more likely to be eQTLs and vice versa. The integration mapped the specific genes underlying the GWAS signals in lung tissue. The eSNP-regulated genes were enriched for developmental and inflammatory pathways; by comparison, SNPs associated with lung function that were eQTLs in blood, but not in lung, were only involved in inflammatory pathways. Lung function eSNPs were enriched for regulatory elements and were over-represented among genes showing differential expression during fetal lung development. An mRNA gene expression signature for COPD was identified in lung tissue and compared with the Connectivity Map. This in-silico drug repurposing approach suggested several compounds that reverse the COPD

		gene expression signature, including a nicotine receptor antagonist. These findings represent novel therapeutic pathways for COPD.<br>Interpretation The system genetics approach identified lung tissue genes driving the variation in lung function and susceptibility to COPD. The identification of these genes and the pathways in which they are enriched is essential to understand the pathophysiology of airway obstruction and to identify novel therapeutic targets and biomarkers for COPD, including drugs that reverse the COPD gene signature in silico.
	**Sport Sciences**	
89.	Marinovic J, Ljubkovic M, Obad A, Breskovic T, Salamunic I, Denoble PJ, Dujic Z. Assessment of extravascular lung water and cardiac function in trimix SCUBA diving. Med Sci Sports Exerc. 2010 Jun;42(6):1054-61. doi: 10.1249/MSS.0b013e3181c5b8a8.	MARINOVIC J., M. LJUBKOVIC, A. OBAD, T. BRESKOVIC, I. SALAMUNIC, P. DENOBLE, and Z. DUJIC. Assessment of Extravascular Lung Water and Cardiac Function in Trimix SCUBA Diving. Med. Sci. Sports Exerc., Vol. 42, No. 6, pp. 1054-1061, 2010. An increasing number of recreational self-contained underwater breathing apparatus (SCUBA) divers use trimix of oxygen, helium, and nitrogen for dives deeper than 60 m of sea water. Although it was seldom linked to the development of pulmonary edema, whether SCUBA diving affects the extravascular lung water (EVLW) accumulation is largely unexplored. Methods: Seven divers performed six dives on consecutive days using compressed gas mixture of oxygen, helium, and nitrogen (trimix), with diving depths ranging from 55 to 80 m. The echocardiographic parameters (bubble grade, lung comets, mean pulmonary arterial pressure (PAP), and left ventricular function) and the blood levels of the N-terminal part of pro-brain natriuretic peptide (NT-proBNP) were assessed before and after each dive. Results: Venous gas bubbling was detected after each dive with mean probability of decompression sickness ranging from 1.77% to 3.12%. After each dive, several ultrasonographically detected lung comets rose significantly, which was paralleled by increased pulmonary artery pressure (PAP) and decreased left ventricular contractility (reduced ejection fraction at higher end-systolic and end-diastolic volumes) as well as the elevated NT-proBNP. The number of ultrasound lung comets and mean PAP did not return to baseline values after each dive. Conclusions: This is the first report that asymptomatic SCUBA dives are associated with accumulation of EVLW with concomitant increase in PAP, diminished left ventricular contractility, and increased release of NT-proBNP, suggesting a significant cardiopulmonary strain. EVLW and PAP did not return to baseline during repetitive dives, indicating possible cumulative effect with increasing the risk for pulmonary edema.
90.	Batinic T, Utz W, Breskovic T, Jordan J, Schulz-Menger J, Jankovic S, Dujic Z, Tank J. Cardiac magnetic resonance imaging during pulmonary	BATINIC, T., W. UTZ, T. BRESKOVIC, J. JORDAN, J. SCHULZ-MENGER, S. JANKOVIC, Z. DUJIC, and J. TANK. Cardiac Magnetic Resonance Imaging during Pulmonary Hyperinflation in Apnea Divers. Med. Sci. Sports Exerc., Vol. 43, No. 11, pp. 2095-2101, 2011. Purpose:

	hyperinflation in apnea divers. Med Sci Sports Exerc. 2011 Nov;43(11):2095-101. doi: 10.1249/MSS.0b013e31821ff294.	Apnea divers hyperinflate the lung by taking a deep breath followed by glossopharyngeal insufflation. The maneuver can lead to symptomatic arterial hypotension. We tested the hypotheses that glossopharyngeal insufflation interferes with cardiac function further reducing cardiac output (CO) using cardiac magnetic resonance imaging (MRI) to fully sample both cardiac chambers. Methods: Eleven dive athletes (10 men, 1 woman; age = 26 +/- 5 yr, body mass indeks = 23.5 +/- 1.7 kg.m(-2)) underwent cardiac MRI during breath holding at functional residual capacity (baseline), at total lung capacity (apnea), and with submaximal glossopharyngeal insufflation. Lung volumes were estimated from anatomic images. Short-axis cine MR images were acquired to study biventricular function. Dynamic changes were followed by long-axis cine MRI. Results: Left and right ventricular end-diastolic volumes (LVEDV, RVEDV) decreased during apnea with and without glossopharyngeal insufflation (baseline: LVEDV = 198 +/- 19 mL, RVEDV = 225 +/- 30 mL; apnea: LVEDV = 125 +/- 38 mL, RVEDV = 148 +/- 37 mL, P < 0.001; glossopharyngeal insufflation: LVEDV = 108 +/- 26 mL, RVEDV = 136 +/- 29 mL, P < 0.001 vs baseline). CO decreased during apnea (left = -29 +/- 4 %, right = -29 +/- 4 %) decreasing further with glossopharyngeal insufflation (left = -38% +/- 4%, right = -39% +/- 4%, P < 0.05). HR increased 16 +/- 4 bpm with apnea and 17 +/- 5 bpm with glossopharyngeal insufflation (P < 0.01). Ejection fraction moderately decreased (apnea: left = -5% +/- 2%, right = -7% +/- 2%, glossopharyngeal insufflation: left = -6% +/- 2%, right = -10% +/- 2%, P < 0.01). With continued apnea with and without glossopharyngeal insufflation, LVEDV and CO increased over time by a similar but small amount (P < 0.01). Conclusions: The major finding of our study was that submaximal glossopharyngeal insufflation decreased CO further albeit by a small amount compared to maximal inspiratory apnea. The response was not associated with severe biventricular dysfunction.
91.	Dujic Z, Ivancev V, Heusser K, Dzamonja G, Palada I, Valic Z, Tank J, Obad A, Bakovic D, Diedrich A, Joyner MJ, Jordan J. Central chemoreflex sensitivity and sympathetic neural outflow in elite breath-hold divers. J Appl Physiol (1985). 2008 Jan;104(1):205-11. doi: 10.1152/japplphysiol.00844.2007.	Repeated hypoxemia in obstructive sleep apnea patients increases sympathetic activity, thereby promoting arterial hypertension. Elite breath-holding divers are exposed to similar apneic episodes and hypoxemia. We hypothesized that trained divers would have increased resting sympathetic activity and blood pressure, as well as an excessive sympathetic nervous system response to hypercapnia. We recruited 11 experienced divers and 9 control subjects. During the diving season preceding the study, divers participated in 7.3 +/- 1.2 diving fish-catching competitions and 76.4 +/- 14.6 apnea training sessions with the last apnea 3-5 days before testing. We monitored beat-by-beat blood pressure, heart rate, femoral artery blood flow, respiration, end-tidal CO2, and muscle sympathetic nerve activity (MSNA). After a baseline period, subjects began to rebreathe a hyperoxic gas mixture to raise end-tidal CO2 to 60 Torr. Baseline MSNA frequency was 31 +/- 11

		bursts/min in divers and 33 +/- 13 bursts/min in control subjects. Total MSNA activity was 1.8 +/- 1.5 AU/min in divers and 1.8 +/- 1.3 AU/min in control subjects. Arterial oxygen saturation did not change during rebreathing, whereas end-tidal CO2 increased continuously. The slope of the hypercapnic ventilatory and MSNA response was similar in both groups. We conclude that repeated bouts of hypoxemia in elite, healthy breath-holding divers do not lead to sustained sympathetic activation or arterial hypertension. Repeated episodes of hypoxemia may not be sufficient to drive an increase in resting sympathetic activity in the absence of additional comorbidities.
92.	Grgic J, Schoenfeld BJ, Skrepnik M, Davies TB, Mikulic P. Effects of Rest Interval Duration in Resistance Training on Measures of Muscular Strength: A Systematic Review. Sports Med. 2018 Jan;48(1):137-151. doi: 10.1007/s40279-017-0788-x.	ackground Rest interval (RI) duration 59 san important resistance-training variable underlying gain in muscular strength. Recommendations for optimal RI duration for gains in muscular strength are largely inferred from studies examining the acute resistance training effects, and the generalizability of such findings to chronic adaptations is uncertain.<br>Objective The goals of this systematic literature review are: (i) to aggregate findings and interpret the studies that assessed chronic muscular strength adaptations to resistance training interventions involving different RI durations, and (ii) to provide evidence-based recommendations for exercise practitioners and athletes.<br>Methods The review was performed according to the PRISMA guidelines with a literature search encompassing five databases. Methodological quality of the studies was evaluated using a modified version of the Downs and Black checklist.<br>Results Twenty-three studies comprising a total of 491 participants (413 males and 78 females) were found to meet the inclusion criteria. All studies were classified as being of good to moderate methodological quality; none of the studies were of poor methodological quality.<br>Conclusion The current literature shows that robust gains in muscular strength can be achieved even with short Ris (<60 s). However, it seems that longer duration Ris (>2 min) are required to maximize strength gains in resistance-trained individuals. With regard to untrained individuals, it seems that short to moderate Ris (60-120 s) are sufficient for maximizing muscular strength gains.
93.	Marinovic J, Ljubkovic M, Obad A, Bakovic D, Breskovic T, Dujic Z. Effects of successive air and trimix dives on human cardiovascular function. Med Sci Sports Exerc. 2009 Dec;41(12):2207-12. doi: 10.1249/MSS.0b013e3181aa04cc.	MARINOVIC, J., M. LYUBKOVIC, A. OBAD, D. BAKOVIC, T. BRESKOVIC, and Z. DUJIC. Med. Sci. Sports Exerc., Vol. 41, No. 12, pp. 2207-2212, 2009. Introduction: The use of trimix (a mixture of oxygen, helium, and nitrogen) has significantly increased among the diver population. However, data indicating how trimix dives at most common depths affect the cardiovascular function are sparse. The purpose of this study was to investigate the cardiovascular effects of trimix dives and compare them with air dives and to determine

		whether the repetition of dives in successive days affects their extent. Methods: Nine professional divers performed four dives in consecutive days where the dive depth was progressively increased to the maximum of 55 m. Divers used air in the first dive, nitrox 25 in the second, and trimix 20/30 in the third and fourth dives. Echocardiography was performed before and after each dive. Results: After each dive, a significantly decreased left ventricular ejection fraction and fractional shortening and an increased end-systolic volume without a change in end-diastolic volume were found, indicating a depressed systolic function of the left side of the heart. Assessment of the ratio between pulmonary artery acceleration time and right ventricular ejection time (used as an indicator of pulmonary artery pressure (PAP)) revealed an increase in PAP after all the dives. No physiologically relevant cumulative effects of the multiple dives or signs of acclimatization were found. Conclusions: The current study shows that the cardiovascular effects of trimix dives do not differ from those of the dives with compressed air. However, it suggests that even a very safe and conservative trimix diving profile exerts significant cardiovascular effects.
94.	Dujić Z, Palada I, Obad A, Duplancić D, Brubakk AO, Valic Z. Exercise-induced intrapulmonary shunting of venous gas emboli does not occur after open-sea diving. J Appl Physiol (1985). 2005 Sep;99(3):944-9. doi: 10.1152/japplphysiol.01431.2004.	Paradoxical arterializations of venous gas emboli can lead to neurological damage after diving with compressed air. Recently, significant exercise-induced intrapulmonary anatomical shunts have been reported in healthy humans that result in widening of alveolar-to-arterial oxygen gradient. The aim of this study was to examine whether intrapulmonary shunts' can be found following strenuous exercise after diving and, if so, whether exercise should be avoided during that period. Eleven healthy, military male divers performed an open-sea dive to 30 in breathing air, remaining at pressure for 30 min. During the bottom phase of the dive, subjects performed mild exercise at similar to 30% of their maximal oxygen uptake. The ascent rate was 9 m/min. Each diver performed graded upright cycle ergometry up to 80% of the maximal oxygen uptake 40 min after the dive. Monitoring of venous gas emboli was performed in both the right and left heart with an ultrasonic scanner every 20 min for 60 min after reaching the surface pressure during supine rest and following two coughs. The diving profile used in this study produced significant amounts of venous bubbles. No evidence of intrapulmonary shunting was found in any subject during either supine resting posture or any exercise grade. Also, short strenuous exercise after the dive did not result in delayed-onset decompression sickness in any subject, but studies with a greater number of participants are needed to confirm whether divers should be allowed to exercise after diving.
95.	Ljubkovic M, Marinovic J, Obad A, Breskovic T, Gaustad SE, Dujic Z. High incidence of venous and	SCUBA diving is associated with generation of gas emboli due to gas release from the supersaturated tissues during decompression. Gas emboli arise mostly on the venous side

	arterial gas emboli at rest after trimix diving without protocol violations. J Appl Physiol (1985). 2010 Dec;109(6):1670-4. doi: 10.1152/japplphysiol.01369.2009.	of circulation, and they are usually eliminated as they pass through the lung vessels. Arterialization of venous gas emboli (VGE) is seldom reported, and it is potentially related to neurological damage and development of decompression sickness. The goal of the present study was to evaluate the generation of VGE in a group of divers using a mixture of compressed oxygen, helium, and nitrogen (trimix) and to probe for their potential appearance in arterial circulation. Seven experienced male divers performed three dives in consecutive days according to trimix diving and decompression protocols generated by V-planner, a software program based on the Varying Permeability Model. The occurrence of VGE was monitored ultrasonographically for up to 90 min after surfacing, and the images were graded on a scale from 0 to 5. The performed diving activities resulted in a substantial amount of VGE detected in the right cardiac chambers and their frequent passage to the arterial side, in 9 of 21 total dives (42%) and in 5 of 7 divers (71%). Concomitant measurement of mean pulmonary artery pressure revealed a nearly twofold augmentation, from 13.6 +/- 2.8, 19.2 +/- 9.2, and 14.7 +/- 3.3 mmHg assessed before the first, second, and the third dive, respectively, to 26.1 +/- 5.4, 27.5 +/- 7.3, and 27.4 +/- 5.9 mmHg detected after surfacing. No acute decompression-related disorders were identified. The observed high gas bubble loads and repeated microemboli in systemic circulation raise questions about the possibility of long-term adverse effects and warrant further investigation.
96.	Gaustad SE, Brubakk AO, Høydal M, Catalucci D, Condorelli G, Dujic Z, Marinovic J, Ljubkovic M, Møllerløkken A, Wisløff U. Immersion before dry simulated dive reduces cardiomyocyte function and increases mortality after decompression. J Appl Physiol (1985). 2010 Sep;109(3):752-7. doi: 10.1152/japplphysiol.01257.2009.	Gaustad SE, Brubakk AO, Hoydal M, Catalucci D, Condorelli G, Dujic Z, Marinovic J, Ljubkovic M, Mollerlokken A, Wisloff U. Immersion before dry simulated dive reduces cardiomyocyte function and increases mortality after decompression. J Appl Physiol 109: 752-757, 2010. First published July 15, 2010; doi: 10.1152/japplphysiol.01257.2009.-Diving and decompression performed under immersed conditions have been shown to reduce cardiac function. The mechanisms for these changes are not known. The effect of immersion before a simulated hyperbaric dive on cardiomyocyte function was studied. Twenty-three rats were assigned to four groups: control, 1 h thermoneutral immersion, dry dive, and 1 h thermoneutral immersion before a dive (preimmersion dive). Rats exposed to a dive were compressed to 700 kPa, maintained for 45 min breathing air, and decompressed linearly to the surface at a rate of 50 kPa/min. Postdive, the animals were anesthetized and the right ventricle insonated for bubble detection using ultrasound. Isolation of cardiomyocytes from the left ventricle was performed and studied using an inverted fluorescence microscope with video-based sarcomere spacing. Compared with a dry dive, preimmersion dive significantly increased bubble production and decreased the survival time (bubble grade 1 vs. 5, and survival time 60 vs. 17 min, respectively).

		Preimmersion dive lead to 18% decreased cardiomyocyte shortening, 20% slower diastolic relengthening, and 22% higher calcium amplitudes compared with controls. The protein levels of the sarco-endoplasmic reticulum calcium ATPase (SERCA2a), Na+/Ca2+ exchanger (NCX), and phospholamban phosphorylation in the left ventricular tissue were significantly reduced after both dry and preimmersion dive compared with control and immersed animals. The data suggest that immersion before a dive results in impaired cardiomyocyte and Ca2+ handling and may be a cellular explanation to reduced cardiac function observed in humans after a dive.
97.	Dujic Z, Breskovic T. Impact of breath holding on cardiovascular respiratory and cerebrovascular health. Sports Med. 2012 Jun 1;42(6):459-72. doi: 10.2165/11599260-000000000-00000.	Human underwater breath-hold diving is a fascinating example of applied environmental physiology. In combination with swimming, it is one of the most popular forms of summer outdoor physical activities. 62 san performed by a variety of individuals ranging from elite breath-hold divers, underwater hockey and rugby players, synchronized and sprint swimmers, spear fishermen, sponge harvesters and up to recreational swimmers. Very few data currently exist concerning the influence of regular breath holding on possible health risks such as cerebrovascular, cardiovascular and respiratory diseases. A literature search of the PubMed electronic search engine using keywords 'breath-hold diving' and 'apnoea diving' was performed. This review focuses on recent advances in knowledge regarding possibly harmful physiological changes and/or potential health risks associated with breath-hold diving. Available evidence indicates that deep breath-hold dives can be very dangerous and can cause serious acute health problems such a collapse of the lungs, barotrauma at descent and ascent, pulmonary oedema and alveolar haemorrhage, cardiac arrest, blackouts, nitrogen narcosis, decompression sickness and death. Moreover, even shallow apnoea dives, which are far more frequent, can present a significant health risk. The state of affairs is disturbing as athletes, as well as recreational individuals, practice voluntary apnoea on a regular basis. Long-term health risks of frequent maximal breath holds are at present unknown, but should be addressed in future research. Clearly, further studies are needed to better understand the mechanisms related to the possible development or worsening of different clinical disorders in recreational or competitive breath holding and to determine the potential changes in training/competition regimens in order to prevent these adverse events.
98.	Baković D, Eterović D, Valic Z, Saratlija-Novaković Z, Palada I, Obad A, Dujić Z. Increased pulmonary vascular resistance and reduced stroke volume in association with CO2 retention and inferior vena cava dilatation. J Appl Physiol (1985). 2006	Increased pulmonary vascular resistance and reduced stroke volume in association with CO2 retention and inferior vena cava dilatation. J Appl Physiol 101: 866-872, 2006. First published May 25, 2006; doi: 10.1152/japplphysiol.00759.2005.-Changes in cardiovascular parameters elicited during a maximal breath hold are well described. However, the impact of consecutive maximal breath holds on central hemodynamics in the postapneic period is

	Sep;101(3):866-72. doi: 10.1152/japplphysiol.00759.2005.	unknown. Eight trained apnea divers and eight control subjects performed five successive maximal apneas, separated by a 2-min resting interval, with face immersion in cold water. Ultrasound examinations of inferior vena cava (IVC) and the heart were carried out at times 0, 10, 20, 40, and 60 min after the last apnea. The arterial oxygen saturation level and blood pressure, heart rate, and transcutaneous partial pressures of CO2 and O-2 were monitored continuously. At 20 min after breath holds, IVC diameter increased (27.6 and 16.8% for apnea divers and controls, respectively). Subsequently, pulmonary vascular resistance increased and cardiac output decreased both in apnea divers (62.8 and 21.4%, respectively) and the control group (74.6 and 17.8%, respectively). Cardiac output decrements were due to reductions in stroke volumes in the presence of reduced end-diastolic ventricular volumes. Transcutaneous partial pressure of CO2 increased in all participants during breath holding, returned to baseline between apneas, but remained slightly elevated during the postdive observation period (similar to 4.5%). Thus increased right ventricular afterload and decreased cardiac output were associated with CO2 retention and signs of peripheralization of blood volume. These results indicate that repeated apneas may cause prolonged hemodynamic changes after resumption of normal breathing, which may suggest what happens in sleep apnea syndrome.
99.	Dujic Z, Uglesic L, Breskovic T, Valic Z, Heusser K, Marinovic J, Ljubkovic M, Palada I. Involuntary breathing movements improve cerebral oxygenation during apnea struggle phase in elite divers. J Appl Physiol (1985). 2009 Dec;107(6):1840-6. doi: 10.1152/japplphysiol.00334.2009.	Involuntary breathing movements improve cerebral oxygenation during apnea struggle phase in elite divers. J Appl Physiol 107: 1840-1846, 2009; doi:10.1152/japplphysiol.00334.2009.-We investigated whether the involuntary breathing movements (IBM) during the struggle phase of breath holding, together with peripheral vasoconstriction and progressive hypercapnia, have a positive effect in maintaining cerebral blood volume. The central hemodynamics, arterial oxygen saturation, brain regional oxyhemoglobin (bHbO(2)), deoxyhemoglobin, and total hemoglobin changes and IBM were monitored during maximal dry breath holds in eight elite divers. The frequency of IBM increased (by similar to 100%), and their duration decreased (similar to 30%), toward the end of the struggle phase, whereas the amplitude was unchanged (compared with the beginning of the struggle phase). In all subjects, a consistent increase in brain regional deoxyhemoglobin and total hemoglobin was also found during struggle phase, whereas bHbO2 changed biphasically: it initially increased until the middle of the struggle phase, with the subsequent relative decline at the end of the breath hold. Mean arterial pressure was elevated during the struggle phase, although there was no further rise in the peripheral resistance, suggesting unchanged peripheral vasoconstriction and implying the beneficial influence of the IBM on the cardiac output recovery (primarily by restoration of the stroke volume). The IBM-induced short-lasting, sudden increases in mean arterial

		pressure were followed by similar oscillations in bHbO(2). These results suggest that an increase in the cerebral blood volume observed during the struggle phase of dry apnea is most likely caused by the IBM at the time of the hypercapnia-induced cerebral vasodilatation and peripheral vasoconstriction.
100.	Valic Z, Palada I, Dujic Z. Short-acting NO donor and decompression sickness in humans. J Appl Physiol (1985). 2007 Apr;102(4):1725; author reply 1726. doi: 10.1152/japplphysiol.01363.2006.	No abstract available
101.	Palada I, Eterovic D, Obad A, Bakovic D, Valic Z, Ivancev V, Lojpur M, Shoemaker JK, Dujic Z. Spleen and cardiovascular function during short apneas in divers. J Appl Physiol (1985). 2007 Dec;103(6):1958-63. doi: 10.1152/japplphysiol.00182.2007.	Spleen and cardiovascular function during short apneas in divers. J Appl Physiol 103: 1958-1963, 2007. First published October 18, 2007; doi:10.1152/japplphysiol.00182.2007.-We investigated the spleen volume changes as related to the cardiovascular responses during short-duration apneas at rest. We used dynamic ultrasound splenic imaging and noninvasive photoplethysmographic cardiovascular measurements before, during, and after 15-20 s apneas in seven trained divers. The role of baroreflex was studied by intravenous bolus of vasodilating drug trinitrosan during tidal breathing. The role of lung volume was studied by comparing the apneas at near-maximal lung volume with apneas after inhaling tidal volume, with and without cold forehead stimulation. In apneas at near maximal lung volume, a 20% reduction in splenic volume (P = 0.03) was observed as early as 3 s after the onset of breath holding. Around that time the heart rate increased, the mean arterial pressure abruptly decreased from 89.6 to 66.7 mmHg (P = 0.02), and cardiac output decreased, on account of reduction in stroke volume. Intravenous application of trinitrosan resulted in similar to 6-mmHg decrement in mean arterial pressure, while the splenic volume decreased for similar to 13%. In apneas at low lung volume, the early splenic contraction was also observed, 10% without and 12% with cold forehead stimulation, although the mean arterial pressure did not change or even increased, respectively. In conclusion, the spleen contraction is present at the beginning of apnea, accentuated by cold forehead stimulation. At large, but not small, lung volume, this initial contraction is probably facilitated by downloaded baroreflex in conditions of decreased blood pressure and cardiac output.
102.	Baković D, Valic Z, Eterović D, Vukovic I, Obad A, Marinović-Terzić I, Dujić Z. Spleen volume and blood flow response to repeated breath-hold apneas. J Appl Physiol (1985). 2003 Oct;95(4):1460-6. doi: 10.1152/japplphysiol.00221.2003.	The purpose of this study was 1) to answer whether the reduction in spleen size in breath-hold apnea 64 san active contraction or a passive collapse secondary to reduced splenic arterial blood flow and 2) to monitor the spleen response to repeated breath-hold apneas. Ten trained apnea divers and 10 intact and 7 splenectomized untrained persons repeated five maximal apneas (A1-A5) with face immersion in cold water, with 2 min interposed between successive attempts. Ultrasonic monitoring of the spleen and noninvasive

		cardiopulmonary measurements were performed before, between apneas, and at times 0, 10, 20, 40, and 60 min after the last apnea. Blood flows in splenic artery and splenic vein were not significantly affected by breath-hold apnea. The duration of apneas peaked after A3 (143, 127, and 74 s in apnea divers, intact, and splenectomized persons, respectively). A rapid decrease in spleen volume (similar to20% in both apnea divers and intact persons) was mainly completed throughout the first apnea. The spleen did not recover in size between apneas and only partly recovered 60 min after A5. The well-known physiological responses to apnea diving, i.e., bradycardia and increased blood pressure, were observed in A1 and remained unchanged throughout the following apneas. These results show rapid, probably active contraction of the spleen in response to breath-hold apnea in humans. Rapid spleen contraction and its slow recovery may contribute to prolongation of successive, briefly repeated apnea attempts.
103.	Heusser K, Dzamonja G, Breskovic T, Steinback CD, Diedrich A, Tank J, Jordan J, Dujic Z. Sympathetic and cardiovascular responses to glossopharyngeal insufflation in trained apnea divers. J Appl Physiol (1985). 2010 Dec;109(6):1728-35. doi: 10.1152/japplphysiol.00522.2010.	Glossopharyngeal insufflation (lung packing) is a common maneuver among experienced apnea divers by which additional air is pumped into the lungs. It has been shown that packing may compromise cardiovascular homeostasis. We tested the hypothesis that the packing-mediated increase in intrathoracic pressure enhances the baroreflex-mediated increase in muscle sympathetic nerve activity (MSNA) in response to an exaggerated drop in cardiac output (CO). We compared changes in hemodynamics and MSNA (peroneal microneurography) during maximal breath-holds without and with prior moderate packing (0.79 +/- 0.40 liters) in 14 trained divers (12 men, 2 women, 26.7 +/- 4.5 yr, body mass indeks 24.8 +/- 2.4 kg/m(2)). Packing did not change apnea time (3.8 +/- 1.0 vs. 3.8 +/- 1.2 min), hemoglobin oxygen desaturation (-17.6 +/- 12.3 vs. -18.7 +/- 12.8%), or the reduction in CO (1 min: -3.65 +/- 1.83 vs. -3.39 +/- 1.96 l/min; end of apnea: -2.44 +/- 1.33 vs. -2.16 +/- 1.44 l/min). On the other hand, packing dampened the early, i.e., 1-min increase in mean arterial pressure (MAP, 1 min: 9.2 +/- 8.3 vs. 2.4 +/- 11.0 mmHg, P < 0.01) and in total peripheral resistance (relative TPR, 1 min: 2.1 +/- 0.5 vs. 1.9 +/- 0.5, P < 0.05) but it augmented the concomitant rise in MSNA (1 min: 28.0 +/- 11.7 vs. 39.4 +/- 12.7 bursts/min, P < 0.001; 32.8 +/- 16.4 vs. 43.9 +/- 14.8 bursts/100 heart beats, P < 0.01; 3.3 +/- 2.1 vs. 4.8 +/- 3.2 au/min, P < 0.05). We conclude that the early sympathoactivation 1 min into apnea after moderate packing is due to mechanisms other than excessive reduction in CO. We speculate that lower MAP despite increased MSNA after packing might be explained by vasodilator substances released by the lungs. This idea should be addressed in future studies.
104.	Smoljanovic T, Bojanic I, Hannafin JA, Hren D, Delimar D, Pecina M. Traumatic and overuse injuries	Background: Junior rowers have competed internationally for over 4 decades, and there are no epidemiological data available on traumatic and overuse injury in this population.

	among international elite junior rowers. Am J Sports Med. 2009 Jun;37(6):1193-9. doi: 10.1177/0363546508331205.	Objective: To define the types of musculoskeletal problems present in international elite-level junior rowers and to determine whether gender, physical stature, rowing discipline, and training programs affect the incidence of reported injuries.<br>Study Design: Descriptive epidemiology study.<br>Methods: Injury data were obtained from a total of 398 rowers (42% female, 58% male) who completed a 4-page questionnaire on injury incidence while participating at the Junior World Rowing Championships in Beijing, People's Republic of China, in August 2007.<br>Results: Overall, 290 (73.8%) reported injuries involved overuse, and 103 (26.2%) were related to a single traumatic event. Female rowers were injured more frequently than male rowers (110.2 vs 90.5 injuries per 100 rowers). In both genders, the most common injury site was the low back followed by the knee and the forearm/wrist. The severity of reported injuries was incidental in 65.1%, minor in 21.4%, moderate in 10.4%, and major in 3.1% of cases. The rowers with traumatic injuries had less rowing experience than the uninjured rowers (median [C] +/- interquartile range [Q] = 3 +/- 3 years vs 4 +/- 3 years; P = .043, Mann-Whitney test). Sweep rowers who changed rowing side during the current season had significantly more acute-onset low back injuries (P = .012, chi(2) test) than those who did not change rowing side during the same period. The incidence of traumatic injuries was significantly lower in rowers who regularly performed more than 10 minutes of posttraining stretching (P = .030, chi(2) test). Athletes who ran more than once a week had more overuse knee injuries than those who ran once or less per week (P = .033, chi(2) test).<br>Conclusion: Elite junior rowers attending the World Rowing Championships reported predominantly overuse injuries of low severity during the current rowing season. Low back injuries were the most frequent complaint of elite-level junior rowers.
105.	Ljubkovic M, Dujic Z, Møllerløkken A, Bakovic D, Obad A, Breskovic T, Brubakk AO. Venous and arterial bubbles at rest after no-decompression air dives. Med Sci Sports Exerc. 2011 Jun;43(6):990-5. doi: 10.1249/MSS.0b013e31820618d3.	Venous and Arterial Bubbles at Rest after No-Decompression Air Dives. Med. Sci. Sports Exerc., Vol. 43, No. 6, pp. 990-995, 2011. Purpose: During SCUBA diving, breathing at increased pressure leads to a greater tissue gas uptake. During ascent, tissues may become supersaturated, and the gas is released in the form of bubbles that typically occur on the venous side of circulation. These venous gas emboli (VGE) are usually eliminated as they pass through the lungs, although their occasional presence in systemic circulation (arterialization) has been reported and it was assumed to be the main cause of the decompression sickness. The aims of the present study were to assess the appearance of VGE after air dives where no stops in coming to the surface are required and to assess their potential occurrence and frequency in the systemic circulation. Methods: Twelve male divers performed six dives with 3 d of rest between them following standard no-

		decompression dive procedures: 18/60, 18/70, 24/30, 24/40, 33/15, and 33/20 (the first value indicates depth in meters of sea water and the second value indicates bottom time in minutes). VGE monitoring was performed ultrasonographically every 20 min for 120 min after surfacing. Results: Diving profiles used in this study produced unexpectedly high amounts of gas bubbles, with most dives resulting in grade 4 (55/69 dives) on the bubble scale of 0-5 (no to maximal bubbles). Arterializations of gas bubbles were found in 5 (41.7%) of 12 divers and after 11 (16%) of 69 dives. These VGE crossovers were only observed when a large amount of bubbles was concomitantly present in the right valve of the heart. Conclusions: Our findings indicate high amounts of gas bubbles produced after no-decompression air dives based on standardized diving protocols. High bubble loads were frequently associated with the crossover of VGE to the systemic circulation. Despite these findings, no acute decompression-related pathology was detected.
	**Tropical Medicine**	
106.	Jerončić A, Nonković D, Vrbatović A, Hrabar J, Bušelić I, Martínez-Sernández V, Lojo Rocamonde SA, Ubeira FM, Jaman S, Jeličić EČ, Amati M, Gomez Morales MA, Lukšić B, Mladineo I. Anisakis Sensitization in the Croatian fish processing workers: Behavioral instead of occupational risk factors? PLoS Negl Trop Dis. 2020 Jan 27;14(1):e0008038. doi: 10.1371/journal.pntd.0008038.	We undertook the first study systematically evaluating the risk of Anisakis-sensitization in Croatian fish-processing workers and potential genetic susceptibility to anisakiasis. AntiAnisakis IgE seroprevalence and risk factors for 600 employees of Croatian fish processing facilities and 466 blood donor controls, were assessed by indirect ELISA targeted with: recombinant Ani s 1 and Ani s 7 allergens, an Anisakis crude extract, the commercial ImmunoCAP kit, and questionnaires. Genetic susceptibility to anisakiasis was evaluated by genotypisation of human leukocytes alleles (HLA). Anti-Anisakis seropositive and a fraction of negative subjects were also assessed by ELISA and Western Blot (WB) for IgG seroprevalence to Trichinella spp. Overall, the observed anti-Anisakis seroprevalence inferred by indirect ELISA was significantly higher in fish processing workers (1.8%, 95% CI 0.9-3.3%) compared to the controls (0%, 0-0.8%). Seven out of 11 Ani s 1 and Ani s 7-positives and none of selected 65 negative sera, tested positive on whole-Anisakis extract (ImmunoCAP), whereas Anisakis crude extract ELISA detected 3.9% (2.4-6.0%) seropositives in fish processing workers, three (14%) of which showed IgE reactivity to milk proteins. The highest risk associated with Anisakis-sensitization among workers was fishing in the free time, rather than any of attributes related to the occupational exposure. Although no association was observed between anti-Anisakis seropositivity and wearing gloves or protective goggles, the majority of workers (92%) wore protective gloves, minimizing the risk for Anisakis sensitization via skin contact. Six HLA alleles within DRB1 gene were significantly associated with seropositivity under dominant, allelic or recessive models. All sera confirmed negative for anti-Trichinella spp. IgG. The study exhaustively covered almost all marine fish processing workers in Croatia, reflecting real-time Anisakis

		sensitization status within the industry, already under the influence of wide array of allergens.
107.	Hrabar J, Trumbić Ž, Bočina I, Bušelić I, Vrbatović A, Mladineo I. Interplay between proinflammatory cytokines, miRNA, and tissue lesions in Anisakis-infected Sprague-Dawley rats. PLoS Negl Trop Dis. 2019 May 15;13(5):e0007397. doi: 10.1371/journal.pntd.0007397.	Background Anisakiasis is an emerging public health problem, caused by Anisakis spp. nematode larvae. Anisakiasis presents as variable and unspecific gastrointestinal and/or allergic clinical symptoms, which accounts for the high rate of misdiagnosed cases. Methodology/Principal findings The aim of this study was to characterize the early cellular (6-72 h p.i.) and molecular (6 h p.i.) immune response and general underlying regulatory mechanism in Anisakis infected rats. Each Sprague-Dawley rat was infected with 10 Anisakis spp. larvae by gastric intubation. Tissues with visible lesions were processed for: i) classic histopathology (HE), immunofluorescence (CD3, iNOS, S100A8/A9), and transmission electron microscopy (TEM); ii) target genes (Il1b, Il6, Il18, Ccl3, Icam1, Mmp9) and microRNA (Rat Immunopathology MIRN-104ZF plate, Quiagen) expression analysis; and iii) global DNA methylation. Histopathology revealed that Anisakis larval migration caused moderate to extensive hemorrhages in submucosal and epimysial/perimysial connective tissue. In stomach and muscle, moderate to abundant mixed inflammatory infiltrate was present, dominated by neutrophils and macrophages, while only mild infiltration was seen in intestine. Lesions were characterized by the presence of CD3(+), iNOS(+), and S100A8/A9(+) cells. The greatest number of iNOS(+) and S100A8/A9(+) cells was seen in muscle. Il6, Il1b, and Ccl3 showed particularly strong expression in stomach and visceral adipose tissues, but the order of expression differed between tissues. In total, three miRNAs were differentially expressed, two in stomach (miRNA-451 and miRNA-223) and two in intestine (miRNA-451 and miRNA-672). No changes in global DNA methylation were observed in infected tissues relative to controls.<br>Conclusions/Significance Anisakis infection induces strong immune responses in infected rats with marked induction of specific proinflammatory cytokines and miRNA expression. Deciphering the functional role of these cytokines and miRNAs will help in understanding the anisakiasis pathology and controversies surrounding Anisakis infection in humans. Author summary Anisakiasis is a zoonotic disease (infection transmitted between animals and humans) contracted by consumption of raw or undercooked seafood contaminated with Anisakis spp. nematode larvae. Anisakiasis usually presents with variable and unspecific gastrointestinal and/or allergic symptoms, which accounts for the high rate of misdiagnosed cases. Due to changes in dietary habits, such as eating raw or undercooked seafood, anisakiasis is considered an emerging public health problem. Despite the increase in number of reported cases worldwide, mechanisms of immune response to this unspecific human pathogen are poorly known. We have shown that in experimentally

		infected rats, Anisakis larvae cause severe hemorrhages and necrotic changes of affected tissues in the early phase of infections. Neutrophils and macrophages were abundantly present in tissue lesions, while eosinophils, hallmark of helminth infections, were scarcely present. We have also demonstrated particularly strong expression of several inflammatory genes. Moreover, we give for the first-time insight into putative regulatory mechanism mediated via a distinct class of RNA molecules. Our study may provide new opportunities for better understanding of cellular and molecular response to Anisakis spp., aiming at development of more specific therapeutics and alleviation of pathologies associated with Anisakis spp. infection.
	**Urology & Nephrology**	
108.	Wright AF, Rudan I, Hastie ND, Campbell H. A 'complexity' of urate transporters. Kidney Int. 2010 Sep;78(5):446-52. doi: 10.1038/ki.2010.206.	Genetic variation in the SLC2A9 gene is a new genetic risk factor for low fractional excretion of uric acid, hyperuricemia, and gout. Its gene product, GLUT9, was previously known as a type II glucose/fructose transporter but is now known to function as a high-capacity uric acid transporter that is expressed in kidney, liver, and several other tissues. Follow-up meta-analyses, including one with data from 28,141 individuals, implicated a total of nine additional loci influencing serum urate concentrations, including six other membrane transporters (SLC17A1, SLC17A3, SLC22A11, SLC22A12, SLC16A9, and ABCG2). Variants in these genes together account for about 5% of the variance in serum urate, two-thirds of which is due to SLC2A9. Using these variants in 'Mendelian randomization' analyses provides a powerful means of dissecting the role of urate in cardiovascular and metabolic diseases, where cause-and-effect influences are difficult to discern due to potential confounding. The results highlight the complex interplay of membrane transporters involved in urate metabolism. They also show how variants of weak effect identified by genome-wide association studies can still be important in identifying novel pathways, including a 'complexity' of new and potentially druggable targets for modifying urate transport. Kidney International (2010) 78, 446-452; doi:10.1038/ki.2010.206; published online 7 July 2010
109.	Kestenbaum B, Glazer NL, Köttgen A, Felix JF, Hwang SJ, Liu Y, Lohman K, Kritchevsky SB, Hausman DB, Petersen AK, Gieger C, Ried JS, Meitinger T, Strom TM, Wichmann HE, Campbell H, Hayward C, Rudan I, de Boer IH, Psaty BM, Rice KM, Chen YD, Li M, Arking DE, Boerwinkle E, Coresh J, Yang Q, Levy D, van Rooij FJ, Dehghan A, Rivadeneira F, Uitterlinden AG, Hofman A, van Duijn CM, Shlipak MG, Kao WH,	Phosphorus is an essential mineral that maintains cellular energy and mineralizes the skeleton. Because complex actions of ion transporters and regulatory hormones regulate serum phosphorus concentrations, genetic variation may determine interindividual variation in phosphorus metabolism. Here, we report a comprehensive genome-wide association study of serum phosphorus concentration. We evaluated 16,264 participants of European ancestry from the Cardiovascular Heath Study, Atherosclerosis Risk in Communities Study, Framingham Offspring Study, and the Rotterdam Study. We excluded participants with an estimated GFR <45 ml/min per 1.73 m2 to focus on phosphorus

	Witteman JC, Siscovick DS, Fox CS. Common genetic variants associate with serum phosphorus concentration. J Am Soc Nephrol. 2010 Jul;21(7):1223-32. doi: 10.1681/ASN.2009111104.	metabolism under normal conditions. We imputed genotypes to approximately 2.5 million single-nucleotide polymorphisms in the Hap Map and combined study-specific findings using meta-analysis. We tested top polynnorphisms from discovery cohorts in a 5444-person replication sample. Polymorphisms in seven loci with minor allele frequencies 0.08 to 0 49 associate with serum phosphorus concentration (P = 3.5 x 10-16 to 3.6 X 10-v). Three loci were near genes encoding the kidney-specific type Ila sodium phosphate co-transporter (SLC34A1), the calcium-sensing receptor (CASR), and fibroblast growth factor 23 (FGF23), proteins that contribute to phosphorus metabolism. We also identified genes encoding phosphatases, kinases, and phosphodiesterases that have yetundetermined roles in phosphorus homeostasis. In the replication sample, five of seven top polymorphisms associate with serum phosphorous concentrations (P < 0.05 for each). In conclusion, common genetic variants associate with serum phosphorus in the general population. Further study of the loci identified in this study may help elucidate mechanisms of phosphorus regulation.
110.	Olden M, Corre T, Hayward C, Toniolo D, Ulivi S, Gasparini P, Pistis G, Hwang SJ, Bergmann S, Campbell H, Cocca M, Gandin I, Girotto G, Glaudemans B, Hastie ND, Loffing J, Polasek O, Rampoldi L, Rudan I, Sala C, Traglia M, Vollenweider P, Vuckovic D, Youhanna S, Weber J, Wright AF, Kutalik Z, Bochud M, Fox CS, Devuyst O. Common variants in UMOD associate with urinary uromodulin levels: a meta-analysis. J Am Soc Nephrol. 2014 Aug;25(8):1869-82. doi: 10.1681/ASN.2013070781.	Uromodulin is expressed exclusively in the thick ascending limb and is the most abundant protein excreted in normal urine. Variants in UMOD, which encodes uromodulin, are associated with renal function, and urinary uromodulin levels may be a biomarker for kidney disease. However, the genetic factors regulating uromodulin excretion are unknown. We conducted a meta-analysis of urinary uromodulin levels to identify associated common genetic variants in the general population. We included 10,884 individuals of European descent from three genetic isolates and three urban cohorts. Each study measured uromodulin indexed to creatinine and conducted linear regression analysis of approximately 2.5 million single nucleotide polymorphisms using an additive model. We also tested whether variants in genes expressed in the thick ascending limb associate with uromodulin levels. rs12917707, located near UMOD and previously associated with renal function and CKD, had the strongest association with urinary uromodulin levels (P<0.001). In all cohorts, carriers of a G allele of this variant had higher uromodulin levels than noncarriers did (geometric means 10.24, 14.05, and 17.67 mu g/g creatinine for zero, one, or two copies of the G allele). rs12446492 in the adjacent gene PDILT (protein disulfide isomerase-like, testis expressed) also reached genome-wide significance (P<0.001). Regarding genes expressed in the thick ascending limb, variants in KCNJ1, SORL1, and CAB39 associated with urinary uromodulin levels. These data indicate that common variants in the UMOD promoter region may influence urinary uromodulin levels. They also provide insights into uromodulin biology and the association of UMOD variants with renal function.

111.	Westland R, Verbitsky M, Vukojevic K, Perry BJ, Fasel DA, Zwijnenburg PJ, Bökenkamp A, Gille JJ, Saraga-Babic M, Ghiggeri GM, D'Agati VD, Schreuder MF, Gharavi AG, van Wijk JA, Sanna-Cherchi S. Copy number variation analysis identifies novel CAKUT candidate genes in children with a solitary functioning kidney. Kidney Int. 2015 Dec;88(6):1402-1410. doi: 10.1038/ki.2015.239.	Copy number variations associate with different developmental phenotypes and represent a major cause of congenital anomalies of the kidney and urinary tract (CAKUT). Because rare pathogenic copy number variations are often large and contain multiple genes, identification of the underlying genetic drivers has proven to be difficult. Here we studied the role of rare copy number variations in 80 patients from the KIMONO study cohort for which pathogenic mutations in three genes commonly implicated in CAKUT were excluded. In total, 13 known or novel genomic imbalances in 11 of 80 patients were absent or extremely rare in 23,362 population controls. To identify the most likely genetic drivers for the CAKUT phenotype underlying these rare copy number variations, we used a systematic in silico approach based on frequency in a large data set of controls, annotation with publicly available databases for developmental diseases, tolerance and haploinsufficiency scores, and gene expression profile in the developing kidney and urinary tract. Five novel candidate genes for CAKUT were identified that showed specific expression in the human and mouse developing urinary tract. Among these genes, DLG1 and KIF12 are likely novel susceptibility genes for CAKUT in humans. Thus, there is a significant role of genomic imbalance in the determination of kidney developmental phenotypes. Additionally, we defined a systematic strategy to identify genetic drivers underlying rare copy number variations.
112.	Pattaro C, Aulchenko YS, Isaacs A, Vitart V, Hayward C, Franklin CS, Polasek O, Kolcic I, Biloglav Z, Campbell S, Hastie N, Lauc G, Meitinger T, Oostra BA, Gyllensten U, Wilson JF, Pichler I, Hicks AA, Campbell H, Wright AF, Rudan I, van Duijn CM, Riegler P, Marroni F, Pramstaller PP; EUROSPAN Consortium. Genome-wide linkage analysis of serum creatinine in three isolated European populations. Kidney Int. 2009 Aug;76(3):297-306. doi: 10.1038/ki.2009.135.	There is increasing evidence for a role of genetic predisposition in the etiology of kidney disease, but linkage scans have been poorly replicated. Here we performed a genome-wide linkage analysis of serum creatinine on 2859 individuals from isolated villages in South Tyrol (Italy), Rucphen (The Netherlands) and Vis Island (Croatia), populations that have been stable and permanently resident in their region. Linkage of serum creatinine levels to loci on chromosomes 7p14, 9p21, 11p15, 15q15-21, 16p13, and 18p11 was successfully replicated in at least one discovery population or in the pooled analysis. A novel locus was found on chromosome 10p11. Linkage to chromosome 22q13, independent of diabetes and hypertension, was detected over a region containing the non-muscle myosin heavy chain type II isoform A (MYH9) gene (LOD score = 3.52). In non-diabetic individuals, serum creatinine was associated with this gene in two of the three populations and in meta-analysis (SNP rs11089788, P-value = 0.0089). In populations sharing a homogeneous environment and genetic background, heritability of serum creatinine was higher than in outbred populations, with consequent detection of a larger number of loci than reported before. Our finding of a replicated association of serum creatinine with the MYH9 gene, recently linked to pathological renal conditions in African Americans, suggests that this

		gene may also influence kidney function in healthy Europeans. Kidney International (2009) 76, 297-306; doi:10.1038/ki.2009.135; published online 22 April 2009
113.	Corre T, Arjona FJ, Hayward C, Youhanna S, de Baaij JHF, Belge H, Nägele N, Debaix H, Blanchard MG, Traglia M, Harris SE, Ulivi S, Rueedi R, Lamparter D, Macé A, Sala C, Lenarduzzi S, Ponte B, Pruijm M, Ackermann D, Ehret G, Baptista D, Polasek O, Rudan I, Hurd TW, Hastie ND, Vitart V, Waeber G, Kutalik Z, Bergmann S, Vargas-Poussou R, Konrad M, Gasparini P, Deary IJ, Starr JM, Toniolo D, Vollenweider P, Hoenderop JGJ, Bindels RJM, Bochud M, Devuyst O. Genome-Wide Meta-Analysis Unravels Interactions between Magnesium Homeostasis and Metabolic Phenotypes. J Am Soc Nephrol. 2018 Jan;29(1):335-348. doi: 10.1681/ASN.2017030267.	Magnesium (Mg2+) homeostasis is critical for metabolism. However, the genetic determinants of the renal handling of Mg2+, which is crucial for Mg2+ homeostasis, and the potential influence on metabolic traits in the general population are unknown. We obtained plasma and urine parameters from 9099 individuals from seven cohorts, and conducted a genome-wide meta-analysis of Mg2+ homeostasis. We identified two loci associated with urinary magnesium (uMg), rs3824347 (P=4.4x10(-13)) near TRPM6, which encodes an epithelial Mg2+ channel, and rs35929 (P=2.1x10(-11)), a variant of ARL15, which encodes a GTP-binding protein. Together, these loci account for 2.3% of the variation in 24-hour uMg excretion. In human kidney cells, ARL15 regulated TRPM6-mediated currents. In zebrafish, dietary Mg2+ regulated the expression of the highly conserved ARL15 ortholog arl15b, and arl15b knockdown resulted in renal Mg2+ wasting and metabolic disturbances. Finally, ARL15 rs35929 modified the association of uMg with fasting insulin and fat mass in a general population. In conclusion, this combined observational and experimental approach uncovered a gene-environment interaction linking Mg2+ deficiency to insulin resistance and obesity.
