# Supplementary material for: Bibliometric and clinical trial registry analysis of the University of Split medical research from 1997 to 2022
Source: Croat Med J. 2025 Apr;66(2):153–63. doi: 10.3325/cmj.2025.66.153 (PMC12093127; doi:10.3325/cmj.2025.66.153)
Supplement: Supplementary Material 2 [file CroatMedJ_66_s015.pdf]

Supplementary Table 2. Publications from trials registered in the ClinicalTrials.gov registry from the University of Split and University Hospital of Split as sponsors

No.	ClinicalTrials.gov NCT number	NCT number stated in the abstract	Published article(s)
1.	NCT00631514	Yes	Pavlicević I, Kuzmanić M, Rumboldt M, Rumboldt Z. Interaction between antihypertensives and NSAIDs in primary care: a controlled trial. Can J Clin Pharmacol. 2008 Fall;15(3):e372-82.
2.	NCT01139463	Yes	Glavina T, Mrass D, Dodig T, Glavina G, Pranić S, Uglešić B. Blood lactate levels in patients receiving first- or second- generation antipsychotics. Croat Med J. 2011 Feb;52(1):41-7. doi: 10.3325/cmj.2011.52.41.
3.	NCT01833949	Yes	Sunj M, Canic T, Jeroncic A, Karelovic D, Tandara M, Juric S, Palada I. Anti-Müllerian hormone, testosterone and free androgen index following the dose-adjusted unilateral diathermy in women with polycystic ovary syndrome. Eur J Obstet Gynecol Reprod Biol. 2014 Aug;179:163-9. doi: 10.1016/j.ejogrb.2014.05.011.<br><br>Sunj M, Canic T, Baldani DP, Tandara M, Jeroncic A, Palada I. Does unilateral laparoscopic diathermy adjusted to ovarian volume increase the chances of ovulation in women with polycystic ovary syndrome? Hum Reprod. 2013 Sep;28(9):2417-24. doi: 10.1093/humrep/det273.
4.	NCT02064361	No	Madden D, Thom SR, Yang M, Bhopale VM, Ljubkovic M, Dujic Z. High intensity cycling before SCUBA diving reduces post-decompression microparticle production and neutrophil activation. Eur J Appl Physiol. 2014 Sep;114(9):1955-61. doi: 10.1007/s00421-014-2925-7.
5.	NCT02118207	No	Madden D, Barak O, Thom SR, Yang M, Bhopale VM, Ljubkovic M, Dujic Z. The impact of predive exercise on repetitive SCUBA diving. Clin Physiol Funct Imaging. 2016 May;36(3):197-205. doi: 10.1111/cpf.12213.
6.	NCT02918656	No	Buljan I, Malički M, Wager E, Puljak L, Hren D, Kellie F, West H, Alfirević Ž, Marušić A. No difference in knowledge obtained from infographic or plain language summary of a Cochrane systematic review: three randomized controlled trials. J Clin Epidemiol. 2018 May;97:86-94. doi: 10.1016/j.jclinepi.2017.12.003.
7.	NCT02980107		
8.	NCT03002610		
9.	NCT03203343	Yes	Jukić M, Pogorelić Z, Šupe-Domić D, Jerončić A. Comparison of inflammatory stress response between laparoscopic and open approach for pediatric inguinal hernia repair in children. Surg Endosc. 2019 Oct;33(10):3243-3250. doi: 10.1007/s00464-018-06611-y.

			Jukić M, Pogorelić Z, Šupe-Domić D, Jerončić A. Comparison of inflammatory stress response between laparoscopic and open approach for pediatric inguinal hernia repair in children. Surg Endosc. 2019 Oct;33(10):3243-3250. doi: 10.1007/s00464-018-06611-y. Epub 2018 Dec 3. PMID: 30511312.
10.	NCT03231813	Yes	Leskur D, Bukić J, Petrić A, Zekan L, Rušić D, Šešelja Perišin A, Petrić I, Stipić M, Puizina-Ivić N, Modun D. Anatomical site differences of sodium lauryl sulfate-induced irritation: randomized controlled trial. Br J Dermatol. 2019 Jul;181(1):175-185. doi: 10.1111/bjd.17633.
11.	NCT03442387	Yes	Buljan I, Tokalić R, Roguljić M, Zakarija-Grković I, Vrdoljak D, Milić P, Puljak L, Marušić A. Framing the numerical findings of Cochrane plain language summaries: two randomized controlled trials. BMC Med Res Methodol. 2020 May 6;20(1):101. doi: 10.1186/s12874-020-00990-4.
12.	NCT03554252		
13.	NCT04601558	Yes	Jurić Petričević S, Buljan I, Bjelanović D, Mrduljaš-Đujić N, Pekez T, Ćurković M, Vojvodić Ž, Pavličević I, Marušić M, Marušić A. Effectiveness of letters to patients with or without Cochrane blogshots on 10-year cardiovascular risk change among women in menopausal transition: 6-month three-arm randomized controlled trial. BMC Med. 2022 Oct 20;20(1):381. doi: 10.1186/s12916-022-02555-2.
14.	NCT03542201	Yes	Buljan I, Tokalić R, Roguljić M, Zakarija-Grković I, Vrdoljak D, Milić P, Puljak L, Marušić A. Comparison of blogshots with plain language summaries of Cochrane systematic reviews: a qualitative study and randomized trial. Trials. 2020 May 25;21(1):426. doi: 10.1186/s13063-020-04360-9.
15.	NCT03534791	Yes	Jakus D, Behmen D, Buljan I, Marušić A, Puljak L. Efficacy of reminders for increasing volunteer engagement in translating Cochrane plain language summaries: a pilot randomised controlled trial. BMJ Evid Based Med. 2021 Apr;26(2):49-50. doi: 10.1136/bmjebm-2020-111378.
16.	NCT04692415	Yes	Vrebalov Cindro P, Krnić M, Modun D, Vuković J, Tičinović Kurir T, Kardum G, Rušić D, Šešelja Perišin A, Bukić J. Comparison of the Impact of Insulin Degludec U100 and Insulin Glargine U300 on Glycemic Variability and Oxidative Stress in Insulin-Naive Patients With Type 2 Diabetes Mellitus: Pilot Study for a Randomized Trial. JMIR Form Res. 2022 Jul 8;6(7):e35655. doi: 10.2196/35655.<br><br>Cindro PV, Krnić M, Modun D, Smajić B, Vuković J. The differences between insulin glargine U300 and insulin degludec U100 in impact on the glycaemic variability, arterial stiffness and the lipid profiles in insulin naïve patients suffering from type two diabetes mellitus - outcomes from cross-over open-label randomized trial. BMC Endocr Disord. 2021 Apr 29;21(1):86. doi: 10.1186/s12902-021-00746-1.

17.	NCT03783819	Yes	Leskur D, Perišić I, Romac K, Šušak H, Šešelja Perišin A, Bukić J, Rušić D, Kladar N, Božin B, Modun D. Comparison of mechanical, chemical and physical human models of in vivo skin damage: Randomized controlled trial. Skin Res Technol. 2021 Mar;27(2):208-216. doi: 10.1111/srt.12932.
18.	NCT04587505	No	Kovač R, Juginović I, Delić N, Velat I, Vučemilović H, Vuković I, Kozomara V, Duplančić B. The Effect of Epidural Analgesia on Quality of Recovery (QoR) after Radical Prostatectomy. J Pers Med. 2022 Dec 27;13(1):51. doi: 10.3390/jpm13010051.
19.	NCT04226482	Yes	Mihanović J, Šikić NL, Mrklić I, Katušić Z, Karlo R, Jukić M, Jerončić A, Pogorelić Z. Comparison of new versus reused Harmonic scalpel performance in laparoscopic appendectomy in patients with acute appendicitis-a randomized clinical trial. Langenbecks Arch Surg. 2021 Feb;406(1):153-162. doi: 10.1007/s00423-020-02039-y.
20.	NCT04817033	No	Vuković I, Duplančić B, Benzon B, Đogaš Z, Kovač R, Pecotić R. Midazolam versus Dexmedetomidine in Patients at Risk of Obstructive Sleep Apnea during Urology Procedures: A Randomized Controlled Trial. J Clin Med. 2022 Oct 2;11(19):5849. doi: 10.3390/jcm11195849.
21.	NCT05346562	Yes	Dujic G, Kumric M, Vrdoljak J, Dujic Z, Bozic J. Chronic Effects of Oral Cannabidiol Delivery on 24-h Ambulatory Blood Pressure in Patients with Hypertension (HYPER-H21-4): A Randomized, Placebo-Controlled, and Crossover Study. Cannabis Cannabinoid Res. 2024 Aug;9(4):979-989. doi: 10.1089/can.2022.0320.
22.	NCT05747989	No	Sunjic Roguljic V, Roguljic L, Kovacic V, Jukic I. A Comparison of Tissue Adhesive Material and Suture as Wound-Closure Techniques following Carpal Tunnel Decompression: A Single-Center Randomized Control Trial. J Clin Med. 2023 Apr 14;12(8):2864. doi: 10.3390/jcm12082864.
23.	NCT05808855	No	Sunjic Roguljic V, Roguljic L, Kovacic V, Bilic I, Jukic I. The Influence of the Tissue Adhesive Material as a Surgical Wound-Closure Technique Following Carpal Tunnel Decompression on Neurological and Functional Outcomes: A Single-Center Randomized Controlled Trial. Cureus. 2024 Jan 31;16(1):e53312. doi: 10.7759/cureus.53312.
